# Evolutionary analysis of the kinesin light chain genes in the yellow fever mosquito *Aedes aegypti*: gene duplication as a source for novel early zygotic genes

**DOI:** 10.1186/1471-2148-10-206

**Published:** 2010-07-08

**Authors:** James K Biedler, Zhijian Tu

**Affiliations:** 1 Department of Biochemistry, Virginia Polytechnic Institute and State University, Fralin Biotech Center, Blacksburg, VA, 24061, USA

## Abstract

**Background:**

The maternal zygotic transition marks the time at which transcription from the zygotic genome is initiated and a subset of maternal RNAs are progressively degraded in the developing embryo. A number of early zygotic genes have been identified in *Drosophila melanogaster *and comparisons to sequenced mosquito genomes suggest that some of these early zygotic genes such as *bottleneck *are fast-evolving or subject to turnover in dipteran insects. One objective of this study is to identify early zygotic genes from the yellow fever mosquito *Aedes aegypti *to study their evolution. We are also interested in obtaining early zygotic promoters that will direct transgene expression in the early embryo as part of a *Medea *gene drive system.

**Results:**

Two novel early zygotic kinesin light chain genes we call *AaKLC2.1 *and *AaKLC2.2 *were identified by transcriptome sequencing of *Aedes aegypti *embryos at various time points. These two genes have 98% nucleotide and amino acid identity in their coding regions and show transcription confined to the early zygotic stage according to gene-specific RT-PCR analysis. These *AaKLC2 *genes have a paralogous gene (*AaKLC1*) in *Ae. aegypti*. Phylogenetic inference shows that an ortholog to the *AaKLC2 *genes is only found in the sequenced genome of *Culex quinquefasciatus*. In contrast, *AaKLC1 *gene orthologs are found in all three sequenced mosquito species including *Anopheles gambiae*. There is only one KLC gene in *D. melanogaster *and other sequenced holometabolous insects that appears to be similar to *AaKLC1*. Unlike *AaKLC2*, *AaKLC1 *is expressed in all life stages and tissues tested, which is consistent with the expression pattern of the *An. gambiae *and *D. melanogaster *KLC genes. Phylogenetic inference also suggests that *AaKLC2 *genes and their likely *C. quinquefasciatus *ortholog are fast-evolving genes relative to the highly conserved *AaKLC1-like *paralogs. Embryonic injection of a luciferase reporter under the control of a 1 kb fragment upstream of the *AaKLC2.1 *start codon shows promoter activity at least as early as 3 hours in the developing *Ae. aegypti *embryo. The *AaKLC2.1 *promoter activity reached ~1600 fold over the negative control at 5 hr after egg deposition.

**Conclusions:**

Transcriptome profiling by use of high throughput sequencing technologies has proven to be a valuable method for the identification and discovery of early and transient zygotic genes. The evolutionary investigation of the KLC gene family reveals that duplication is a source for the evolution of new genes that play a role in the dynamic process of early embryonic development. *AaKLC2.1 *may provide a promoter for early zygotic-specific transgene expression, which is a key component of the *Medea *gene drive system.

## Background

The early embryo is transcriptionally inactive and all RNAs present that are needed for embryonic development have been maternally deposited during oogenesis. The onset of zygotic transcription marks the beginning of the maternal-zygotic transition (MZT) whereby maternal RNAs are progressively degraded and zygotic transcription takes over, providing the necessary gene products for development [1-4]. Recent large-scale studies have tried to determine what genes or groups of genes are expressed during Drosophila development [1,2]. At least some of the early zygotic genes such as *bottleneck *[5] are fast-evolving or subject to turnover in dipteran insects because a homolog of the *Drosophila bottleneck *is not found in any of the sequenced mosquito genomes. To study the evolution of early zygotic genes through comparative analysis, we set out to identify early zygotic genes in the yellow fever mosquito *Aedes aegypti *by transcriptome profiling, taking advantage of large-scale Illumina sequencing. We have determined the MZT starting in *Ae. aegypti *within 2-3 hr after egg deposition, based on Illumina transcriptome profiling and RT-PCR. This time is just before or at the beginning of pole cell formation at 3 hr [6]. Early zygotic transcription at this time is developmentally consistent with reports from Drosophila that indicate transcription starting as early as cycle 8 [7], also just before the onset of pole cell formation. We focused on the purely zygotic genes that do not have a maternal contribution. Thus, we were searching for transcripts that were not present in the embryos 0-2 hr after egg deposition but started to appear 2-4 hr after egg deposition.

One of the early and pure zygotic genes identified was a kinesin light chain (KLC) gene that is the focus of this study. The three classes of cytoskeleton molecular motors include kinesin, dynein, and myosin, with kinesin and dynein using microtubules as a track for transport and myosin depending on actin filaments [8]. Kinesins are a diverse family of proteins as demonstrated by phylogenies based on the heavy chain [9,10] and are involved in a variety of cellular transport roles [11]. The conventional kinesin (kinesin-I) is a heterotetramer having two kinesin heavy chains characterized by ATPase-dependent motor activity and two kinesin light chains (KLCs). KLCs are accessory proteins that have a C-terminal tetratricopeptide repeat (TPR) domain comprising approximately 34 residues that binds cargo for transport [11]. Here we report the discovery of two novel embryonic-specific KLC genes we call *AaKLC2 *in *Aedes aegypti *(*Ae. aegypti*) and their likely ortholog in *C. quinquefasciatus*. We analyze these genes in comparison to other KLC genes in diverse organisms and suggest gene duplication as a source for the purely zygotic *AaKLC2 *genes, which are fast-evolving.

Another goal of this research was related to efforts to develop a gene drive system for mosquitoes, which may be used to spread pathogen-resistant genes into mosquito populations to control mosquito-borne infectious disease. A natural gene drive system comprised of maternal effect dominant embryonic arrest (*Medea*) factors was first discovered in the flour beetle *T. castaneum *[12]. In this system, *Medea *is comprised of a maternally expressed toxin that is passed on to all embryos of a *Medea*-bearing female, and a tightly linked zygotically expressed antidote that is only made in *Medea*-bearing embryos. As a result, only *Medea*-bearing offspring of a *Medea*-bearing female will survive, which leads to the fixation of the *Medea *allele in the population. Chen *et al*. 2007 successfully engineered and demonstrated a *Medea *system in *D. melanogaster *that employs the use of maternal-specific and zygotic-specific promoters to drive expression of toxin and antidote genes, respectively [13]. Developing a *Medea *system in mosquitoes requires the identification of early zygotic genes in the mosquito species of interest due to the difficulty of finding orthologs to *D. melanogaster *zygotic genes as mentioned above. We identified the zygotic-specific *AaKLC2.1 *and demonstrated strong promoter activity of its upstream sequence in *Ae. aegypti *early embryos.

## Results

### Identification of two early zygotic kinesin light chain genes *AaKLC2.1 *and *AaKLC2.2*

We looked at expression from *Ae. aegypti Liverpool *strain embryos at 4 time ranges of 0-2, 2-4, 4-8, and 8-12 hr. Over 16,000 *Ae. aegypti *annotated transcripts (vectorbase.org) were used in BLAST [14] vs. sequences obtained from Illumina. Of particular interest for the discovery of pure early zygotic genes were the transcripts having no hits in the 0-2 hr (presence indicates a maternal transcript), and hits present at 2-4 hr. Several transcripts were identified with these criteria, including two KLC genes *AaKLC2.1 *(AAEL011410-RA) and its paralog *AaKLC2.2 *(AAEL014967-RA). The nomenclature here does not correspond to mammalian KLC nomenclature because the focus is on insect KLC genes. *AaKLC2.1 *and *AaKLC2.2 *are 98% identical to each other in their coding regions and it was possible to design gene-specific primers to independently confirm their expression profiles by RT-PCR as described later. Shown in Table 1 is the Illumina result of three KLC genes in this study, one of which is not purely zygotic, but ubiquitously expressed (AAEL012472, see below).

**Table 1:** Kinesin Light Chain Gene Transcription Profile By Illumina Sequencing

Transcript ID/Query	CDS Length	0-2 hr	2-4 hr	4-8 hr	8-12 hr
AAEL011410-RA	1419	0 (0)	250 (366)	405 (728)	0 (0)

AAEL014967-RA	1419	0 (0)	240 (352)	436 (784)	1 (4)

AAEL012472-RA	1536	62 (193)	1103 (1493)	311 (516)	140 (516)

Notes: 0-2, 2-4, 4-8, and 8-12 hr indicate time ranges from which RNA was isolated from developing *Ae. aegypti *embryos. Raw values of the three transcripts in the four samples (number of hits from BLAST using an E-value cutoff of 1e^-7^) are given at left. Normalized values (in parentheses) are calculated as the raw values divided by the transcript (CDS) length, then divided by the total number of reads matching all annotated mRNA transcripts from each sample, and multiplied by 1e^10^. The total number of reads matching all annotated mRNA transcripts from each sample time point was: 0-2 hr (2087707); 2-4 hr (4810435); 4-8 hr (3920821); 8-12 hr (1767898). See Additional File 1 for a summary of the Illumina data. A FASTA file of all Illumina sequence hits that match the three kinesin light chain transcripts in all four samples is provided as Additional File 2.

### Structural and genomic analysis of *AaKLC2 *and other KLC genes

**Figure 1:**
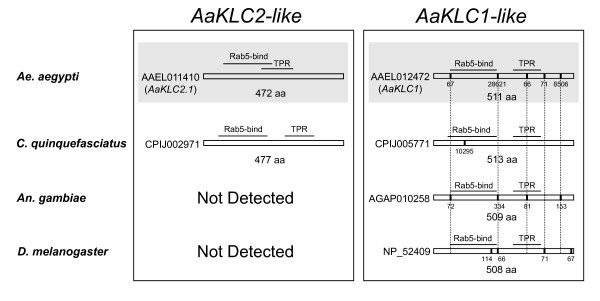
**Coding sequence structure and domains of kinesin light chain genes of mosquitoes and *D. melanogaster***. On the left are the *AaKLC2-like *genes and on the right are the *AaKLC1-like *genes. Vertical bars show location of introns and intron size is given below bars. Note conservation of gene structure (vertical lines show conservation of intron position). Intron sizes are shown as number of bases and are given below vertical bars. Rab5-bind, Rabaptin 5-binding domain. TPR, tetratricopeptide repeat domain. Shaded parts indicate those genes that have been analyzed by RT-PCR and Illumina transcriptome profiling (only *AaKLC2.1 *is shown). Predicted introns are taken from Vectorbase.org and Ensembl.org.

Structural analysis supports the categorization of two KLC gene groups, the *AaKLC1-like *and the *AaKLC2-like *genes (Figure 1). *AaKLC2.1 *is an intronless gene with a predicted open reading frame (ORF) of 1419 nucleotides (nt). *AaKLC2.2 *is also intronless and has 98% nt and aa identity to *AaKLC2.1 *in its coding region. The lack of introns in the *AaKLC2-like *genes is notable since it has been reported that 70% of Drosophila early zygotic genes do not have introns presumably for increased efficiency of transcription with fast-cycling nuclei [1]. A likely ortholog of the *AaKLC2 *genes was identified in *C. quinquefasciatus*, which is an intronless gene having a similar ORF length (gene ID: CPIJ002971). However, no similar genes were found in the genome sequences of *An. gambiae *[15], *An. stephensi *(8× coverage genome assembly, Tu unpublished), *D. melanogaster*, or other insects. Thus from genome analysis, it appears that *AaKLC2-like *genes are restricted to mosquitoes of the subfamily Culicinae. The expression of the *C. quinquefasciatus *gene will need to be verified by RT-PCR or other methods to determine if it is indeed an early and transient zygotic gene. This is why we have not called it *CqKLC2*.

Unlike the *AaKLC2-like *genes, *AaKLC1-like *genes have longer ORFs and at least one intron (Figure 1, right column). Interestingly, these *AaKLC1-like *genes have conservation of intron position in some cases. For example, 4 introns have conserved positions between *Ae. aegypti *and *An. gambiae*, and one intron position is conserved in *Ae. aegypti*, *An. gambiae*, and *D. melanogaster*. In addition, one intron position and length is conserved between *Ae. aegypti *and *D. melanogaster*.

**Figure 2:**
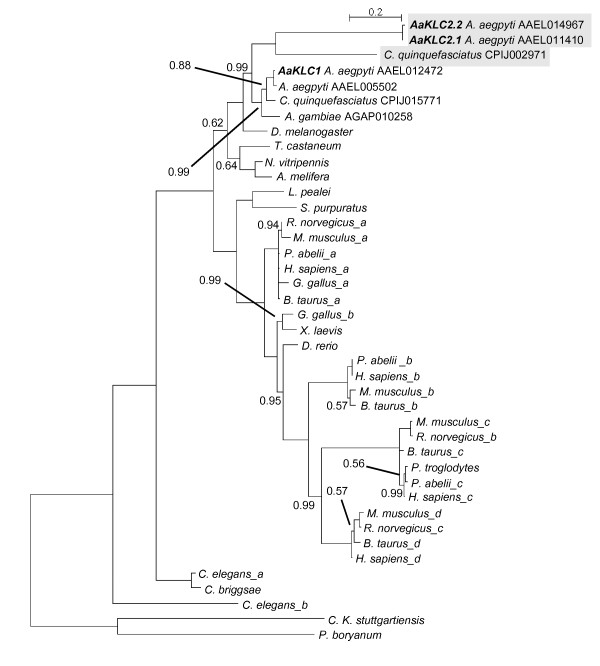
**Kinesin light chain phylogeny**. Phylogenetic inference performed using MrBayes v3.1.2 with aa sequences conceptually translated from kinesin light chain genes of mosquitoes, other holometabolous insects, and divergent taxa. Clade credibility values are shown at each node only when they are not equal to 1.0. Accession numbers are given for mosquito sequences. Letters after species names indicate multiple KLC sequences for a species and are not associated with KLC nomenclature. See Additional File 3 for sequences and accession numbers, and Additional File 4 for alignment in FASTA format. Tree is rooted using kinesin light chain sequences from two bacterial species. Shaded sequences designate zygotic (*AaKLC2.1 *and *AaKLC2.2*) and potential zygotic (*C. quinquefasciatus *CPIJ00CP2971) genes. Bold letters highlight *Ae. aegypti *KLC genes. Scale shows number of aa changes per site. *An. stephensi KLC1 *is not included because we did not have the genome sequence at the time of phylogeny construction. AAEL005502 is not labeled *AaKLC1.2 *because this gene is a partial duplication of *AaKLC1 *and has only the Rab5-binding domain (see text).

All of the KLC genes have tetratricopeptide repeat (TPR) and Rab5-binding domains as determined by the Conserved Domain Database (CDD) v2.17 at the National Center for Biotechnology Information (NCBI) website at http://www.ncbi.nlm.nih.gov/ (Figure 1). Rab5 is a small GTPase that regulates early endosome fusion [16,17]. The Rab-5 binding and TPR domains detected in the 472 aa sequence of *AaKLC2.1 *have E-values of 1e^-26 ^and 3e^-3^, respectively. It is interesting to note that there is a partial gene duplication (gene ID: AAEL005502, see Figure 2) of *AaKLC1 *that contains only the Rab5 binding domain and has 100% nt identity to *AaKLC1 *throughout 729 of the 741 bases of the AAEL005502 ORF (differences are only found in the last 12 bases). The 246 aa conceptual translation of AAEL005502 only has 2 aa different than *AaKLC1*.

### Phylogenetic inference of KLC genes demonstrates a diverse evolutionary history

Published kinesin phylogenies have focused on the kinesin heavy chain. Here we present a KLC phylogeny including representatives from mosquito, fly, wasp, honeybee, worm, fish, frog, sea urchin, squid, monkey, cow, chicken, mouse, rat, human, and bacteria (Figure 2). Like kinesin heavy chain phylogeny, KLC phylogeny is also rather diverse having many paralogous groups, with most of the diversity existing in mammals. For example, humans have 4 paralogous KLC genes. In contrast, the holometabolous insects surveyed have 1 KLC gene in common that includes *AaKLC1 *in *Ae. aegypti*. The *AaKLC2 *genes that are zygotic-specific (see below) and their likely ortholog in *C. quinquefasciatus *appear to be restricted to species of Culicinae because similar sequences could not be detected in the genome sequences of the mosquito *An. gambiae *or other insects. Interestingly, the *AaKLC2 *genes and the *C. quinquefasciatus AaKLC2-like *gene are fast-evolving genes relative to the highly conserved *AaKLC1-like *genes. Shown are the only KLC genes detected in insects resulting from BLAST searches using divergent KLC genes from the phylogeny as queries.

The phylogeny indicates that the *AaKLC2-like *genes arose prior to the divergence of *Ae. aegypti *and *An. gambiae *lineages, suggesting an event that predates the divergence of Culicinae and Anophelinae. This is perplexing because *AaKLC2-like *genes are not detected in Anopheline mosquitoes *An. gambiae *and *An. stephensi*, which suggests that *AaKLC2-like *genes arose within Culicinae after Culicinae/Anophelinae divergence and prior to the divergence of Aedes and Culex genera. Also curious is that *Ae. aegypti *AAEL11410 and *C. quinquefasciatus *CPIJ002971 KLC aa sequences do not obtain each other as reciprocal best hits by blastp. However, the structural and phylogenetic inference supports their grouping (the clade credibility value is 0.99 for the node at the divergence of *KLC1-like *and *KLC2-like *clades). Genomic survey of more species will be needed to elucidate this matter.

### Expression analysis indicates that *AaKLC2 *genes are transiently expressed in the early zygotic stage while *AaKLC1 *is ubiquitously expressed

**Figure 3:**
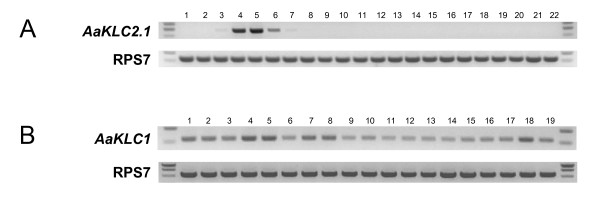
**RT-PCR expression profile of the early zygotic kinesin light chain gene *AaKLC2.1 *and the ubiquitously transcribed paralog *AaKLC1***. Thirty cycles of PCR were performed on cDNA synthesized using total RNA isolated from different life stages and ovaries. Negative control reactions using primers for RPS7 and *AaKLC2.1 *with no reverse transcriptase were performed to check for genomic DNA contamination (not shown). RPS7-specific primers were used for a loading control (bottom). A) *AaKLC2.1*. Lanes 1-11, embryos collected at 0-1, 1-2, ... and 10-11 hr after egg deposition. Lane 12, 12-24 hr embryo; lane 13, 24-40 hr embryo; lane 14, 1-4th instar larvae; lane 15, 0-48 hr pupae; lane 16, ovary from newly emerged females (0-1 day old); lane 17, ovary from 3-4 day old females; lane 18, ovary from females 48 hr post blood meal; lane 19, carcass minus ovaries, 48 hr post blood meal; lane 20, ovary from females 72 hr post blood meal; lane 21, 0-5 day old males, lane 22, 0-5 day old females. Note the faint bands in lane 3 and 7 (2-3 hr and 6-7 hr embryo). B) *AaKLC1*. Lanes 1-15 (same as above); lane 16, 0-5 day old males; lane 17, 0-5 day old females; lane 18, ovary from females 48 hr post blood meal; lane 19, carcass minus ovaries, 48 hr post blood meal.

The first indication of *AaKLC2.1 *and *AaKLC2.2 *as early and transiently expressed zygotic genes came from embryonic transcriptome profiling (see above, Table 1). Transcripts were absent in the 0-2 hr sample and had no hits in the 8-12 hr sample (*AaKLC2.2 *had one hit), but hits were present in both 2-4 and 4-8 hr samples. This profile was validated by RT-PCR using 1 hr time intervals from 0-11 hr embryos (Figure 3, only *AaKLC2.1 *is shown). Furthermore, RT-PCR with RNA from other tissues and life stages shows that their expression is restricted to the early embryo. Transcription begins as early as 2-3 hr and only a faint band can be detected at 6-7 hr, with the large majority of intensity found during the 3-5 hr time range. *AaKLC2.2 *has an almost identical expression profile to *AaKLC2.1 *according to RT-PCR (not shown). RT-PCR products were cloned, sequenced, and verified to be specific for each transcript. These data support that *AaKLC2 *genes are early zygotic transiently expressed genes whose expression is restricted to the early embryonic life stage. In contrast, *AaKLC1 *is expressed in all developmental stages and tissues surveyed by RT-PCR. It also has significant presence in the 0-2 and 8-12 hr Illumina sample (Table 1).

Expression data from ESTs and microarray experiments for KLC genes is consistent with the hypothesis that *AaKLC2-like *genes (Figure 1, left) are zygotic-specific genes and *AaKLC1-like *genes (Figure 1, right) are ubiquitously expressed genes (Table 2). Using BLAST by NCBI, EST hits for various life stages are detected for the *AaKLC1-like *genes in Table 2, and for the other holometabolous insects in Figure 2 (not shown). However, no EST hits for *AaKLC2-like *genes are detected. This is most likely simply explained by the lack of EST data from embryos. Microarray experiments covering developmental time periods for *An. gambiae *reveal expression for the *AaKLC1-like *but not for *AaKLC2-like *genes. These data further support the existence of two distinct types of KLC genes.

**Table 2:** Evidence for Expression of Kinesin Light Chain Genes

Species	Gene ID	Developmental Stage
*Ae. aegypti*	AAEL011410 (AaKLC2.1)	early embryo^1^

*Ae. aegypti*	AAEL014967 (AaKLC2.2)	early embryo^1^

*Ae. aegypti*	AAEL012472 (AaKLC1)	all life stages^1^

*An. gambiae*	AGAP010258 (AaKLC1-like)	various developmental stages^2^

*D. melanogaster*	FBgn0010235 (AaKLC1-like)	various developmental stages^3^

^1 ^These data are from RT-PCR and transcriptome profiling described in this study

^2 ^Expression in various developmental stages/tissues including adult is also reported by various microarray experiments [26-30] from http://www.vectorbase.org/index.php, and from the *Anopheles gambiae *gene expression profile database from UC Irvine, http://www.angaged.bio.uci.edu/)

^3 ^From EST hits by BLAST on NCBI http://blast.ncbi.nlm.nih.gov/

### Early zygotic promoter activity of *AaKLC2.1 *upstream sequence in *Ae. aegypti *embryos

**Figure 4:**
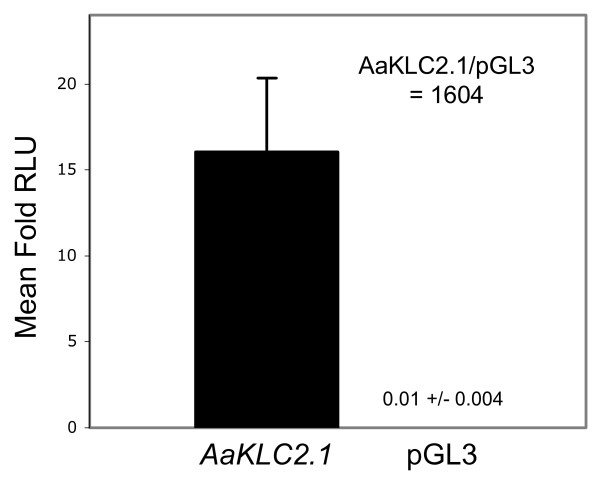
**Early zygotic promoter activity of *AaKLC2.1 *upstream sequence in *Ae. aegypti *embryos**. Embryos were injected in triplicate with either one of the following firefly luciferase reporter plasmids: pGL3-basic only (negative control, shown as pGL3) or pGL3-basic containing 1042 bp of *AaKLC2.1 *5'UTR and upstream sequence. A *Renilla *luciferase reporter pRL-null containing *D. melanogaster *actin 5C promoter was used as an injection control. Embryos were homogenized and assayed at 5 hr after deposition. Mean fold relative light units (RLUs) +/- standard error is shown. The mean fold RLU is calculated as the ratio of the firefly luciferase activity to the *Renilla *luciferase activity. The mean fold activity of *AaKLC2.1 *was 1604 times greater than pGL3-basic.

The 1 kb upstream sequence relative to the predicted start codon of *AaKLC2.1 *was synthesized (Epoch Biolabs, INC) to test for promoter activity. This sequence was cloned into the pGL3-basic luciferase reporter vector, injected into *Ae. aegypti *embryos, and assayed for luciferase activity. *AaKLC2.1 *upstream sequence clearly demonstrates promoter activity, as the mean fold activity of *AaKLC2.1*_pGL3-basic is 1604X greater than the empty vector alone (Figure 4). The 1 kb upstream sequence for *AaKLC2.2 *was also tested and it demonstrated similar activity (not shown). Several independent experiments have demonstrated the activity of these sequences and we are now working towards defining the minimal promoter sequence. The transcription profile of *AaKLC2.1 *is solely zygotic (Figure 3A) and the reporter assay clearly indicates early zygotic activity of the *AaKLC2.1 *promoter. However, the transient assay described here does not directly test whether the promoter is solely zygotic. Transgenic lines of *Ae. aegypti *are needed to directly answer such a question.

## Discussion

### A novel set of purely zygotic genes: duplications, fast evolution, and possible function

*AaKLC2-like *genes are only found in Culicinae mosquitoes *Ae. aegypti *and *C. quinquefasciatus*. However the *AaKLC1-like *genes are found in all surveyed holometabolous insects and are quite conserved in their structure and expression profiles. Thus it is likely that *AaKLC2 *genes are the paralogs that took on a new expression profile and possibly a new function in Culicinae. It is also interesting that in addition to the duplication that gave rise to the *AaKLC2 *gene lineage, there has been another duplication resulting in *AaKLC2.1 and AaKLC2.2*.

The *AaKLC2-like *genes appear to be fast-evolving genes according to their branch length (Figure 2). For example, *AaKLC2.1 *and the *C. quinquefasciatus AaKLC2-like *gene (CPIJ002971) have 44% aa identity, while *AaKLC1 *and the *C. quinquefasciatus AaKLC1-like *gene (CPIJ015771) have 94% aa identity. A curious question is whether *AaKLC2-like *genes have been under positive selection. However, we were unable to perform nonsynonymous/synonymous (dN/dS) analysis due to the divergence of the *AaKLC2 *and the *C. quinquefasciatus *CPIJ002971.

The presence of at least one highly conserved KLC gene (e.g. *AaKLC1-like*) in all of insects is not surprising given its indispensable role in intracellular transport, but it is interesting why a novel zygotic-specific KLC arose in Culicinae. The genome sizes of *Ae. aegypti *and *C. quinquefasciatus *are approximately three and two times larger than *An. gambiae *[18] possibly providing more evolutionary freedom for gene expansion early in Culicinae. A large proportion of Drosophila early and transient zygotic genes were found to be involved in neurogenesis and dorso-ventral patterning [2]. Also noteworthy is that a KLC gene has been implicated in translocation of dorsal determinants in Xenopus [19]. Although we do not have experimental evidence of *AaKLC2 *function in the embryo, it seems plausible that these genes may play a similar role in early embryonic development, given their early and transient zygotic-specific expression profile. Specialization of KLC genes is a recurrent event in evolution. In rat and mouse it was found that KLC3 is restricted to spermatid tails while KLC1 and KLC2 are only found in testis before meiosis [20]. A rat KLC isoform was found to associate with mitochondria in cultured cells [21]. In mouse it was also found that two KLCs were present in the central and peripheral nervous system, but one is enriched in sciatic nerve exons and the other is enriched in the olfactory bulb glomeruli [22]. These examples of KLC specialization are from mammals and are consistent with the KLC diversity in mammals demonstrated in Figure 2. Therefore, the fact that we can only find one conserved KLC gene in holometabolous insects (*AaKLC1*) underscores the generation of *AaKLC2 *genes as an interesting evolutionary event, demonstrating the potential for KLC specialization in this taxonomic group.

### Potential applications of an early zygotic promoter in the context of the Medea gene drive system

We demonstrated that the promoter region of the two *AaKLC2 *genes can direct early zygotic transcription in the *Ae. aegypti *embryo. Thus this promoter may be useful to direct the expression of the antidote gene as part of the *Medea *gene drive system [12,13]. The *AaKLC2 *genes are expressed specifically, transiently, and early in the embryonic stage, which makes them good candidates to drive the antidote expression. The antidote needs to be delivered at the appropriate time for embryonic rescue, providing the replacement of mRNA that was targeted by the maternal toxin. In addition, lack of antidote expression in the ovary will eliminate antagonist effects to the toxin. Lastly, purely zygotic expression will eliminate concerns of ectopic expression that could reduce fitness and thus reduce gene drive efficiency.

## Conclusions

We have discovered a novel set of KLC genes in *Ae. aegypti *that we call *AaKLC2.1 and AaKLC2.2*, and have demonstrated that they are purely zygotic. These are the first reported in any mosquito species and they have a newly evolved expression pattern relative to the *AaKLC1 *genes. We have also identified a likely *AaKlC2 *gene ortholog in *C. quinquefasciatus*. Its similar structure and lack of representation in ESTs suggests that it is also a zygotic gene. *AaKLC2-like *genes appear to be restricted to Culicinae, as they are not detected in *An. gambiae *or *An. stephensi*, or other genome sequences. Genomic survey of more species and additional expression analyses will be needed to test these hypotheses. Data from ESTs and hybridization experiments in addition to our transcriptome and expression analysis by RT-PCR support the existence of two distinct KLC gene groups, the zygotic *AaKLC2-like *genes, and the highly conserved *AaKLC1-like *genes that are found in all mosquitoes and insects surveyed. We also demonstrated strong promoter activity of the *AaKLC2 *gene upstream sequence in *Ae. aegypti *early embryos. Large-scale transcriptome profiling has proven to be a valuable method for the identification of early and transient zygotic-specific genes. These technologies have allowed us to rapidly achieve results for a specific objective. Moreover, as we are currently acquiring and analyzing more transcriptome data, we will be able to investigate the complex and dynamic expression of genes in the early embryo and conduct comparative evolutionary studies with other mosquito species and Drosophila.

## Methods

### Illumina Sequencing

Total RNA was isolated using TRIZOL RNA isolation reagent (Molecular Research Center) from *Ae. aegypti Liverpool *strain embryos at time points 0-2, 2-4, 4-8, and 8-12 hr. RNA was sent to Illumina (illumina.com) for poly-A RNA transcriptome sequencing. Approximately 4.4-6.1 million mappable reads of 33 nt in length were obtained per sample (Additional File 1). Annotated transcripts were used as queries for blastn searches against the Illumina transcriptome sequence reads in different samples, to count the number of sequence reads per annotated transcript per sample. E-value of 1e-7 was used as cutoff for the BLAST analysis. Normalization method is described in the footnotes of Table 1. See Additional File 1 for a tabulated summary of the Illumina RNA sequence data and Additional File 2 for a FASTA file of the Illumina reads that matched the three KLC transcripts AAEL011410, AAEL014967, and AAEL012472.

### Mosquito rearing and embryo collection

*Ae. aegypti Liverpool *strain mosquitoes were reared in incubators at 28°C and 60% relative humidity on a 16 hr light/8 hr dark photoperiod. Larvae and pupae were fed Sera Micron Fry Food with brewer's yeast, and Purina Game Fish Chow. Mosquitoes were blood-fed on female Hsd:ICR (CD-1^®^) mice (Harlan Laboratories http://www.harlan.com). Embryos were collected anywhere from 72 to 96 hr post blood meal.

### Phylogenetic Inference

Phylogenetic Inference was performed using MrBayes v3.1.2 [23]. A total of forty-one conceptually translated amino acid (aa) sequences were obtained from NCBI. Sequences were aligned using ClustalX v2.0 [24] (pairwise alignment parameters: gap opening = 10.0, gap extend = 0.1; multiple alignment parameters: gap opening = 10.0, gap extend = 0.2). The final alignment of approximately 400 aa used for phylogenetic inference was cropped to include obvious regions of conservation and contained the predicted TPR and Rab5-binding domains (see Additional File 3 for aa sequences and Additional File 4 for alignment in FASTA format). MrBayes was used to test 10 fixed-rate aa models, choosing the Jones model exclusively (posterior probability = 1.0) after running 700,000 generations. The tree is rooted using KLC sequences from two prokaryotic species.

### RNA isolation and RT-PCR

Total RNA was isolated for different life stages and ovaries from *Ae. aegypti Liverpool *strain mosquitoes using the TRIZOL RNA isolation reagent (Molecular Research Center). RNA was treated with Turbo DNA-*free *(Ambion) to remove any residual genomic DNA. 1 ug total RNA was used for cDNA synthesis with the First Strand cDNA Synthesis Kit and Oligo dT_20 _(Invitrogen). The final 21 microliter (ul) reaction volume was diluted by adding 21 ul of nuclease-free H20. One ul of this was used for 30 cycles of PCR with Taq Polymerase, dNTPs, and 10× buffer (Takara). Genomic and transcript sequences from the *Ae. aegypti *genome [25] were retrieved from http://aaegypti.vectorbase.org/index.php and http://www.ensembl.org/index.html. A 467 base region of *AaKLC2.1 *(transcript ID: AAEL011410-RA) cDNA was amplified using forward primer 5'-CTACCAGAAGCCATCAAACAT-3' Tm = 60.5°C, and reverse primer 5'- GATTCACTGTCACCATTCTGTGT-3' Tm = 62.8°C. A 464 base region of *AaKLC2.2 *(transcript ID: AAEL011410-RA) cDNA was amplified using forward primer 5'- CCCGAAGCCATTAAACAC-3' Tm = 60.5°C and reverse primer 5'- TGATTCACTGTCACCATTCTGTAC-3' Tm = 62.7°C. Both of these PCR products were cloned and sequenced to verify the primer's specificity for each transcript. A 550 base region of *AaKLC1 *(transcript ID: AAEL012472-RA) cDNA spanning 3 introns was amplified using forward primer 5'- ACTATGTTGAACATCCTGGCTTTAG-3' Tm = 63.1°C, and reverse primer 5'- TCGATTTGTTCTCTTCTCTTTCTTC-3' Tm = 62.8°C. The expected genomic product size is 28.8 kb. As a control for cDNA synthesis and for gel loading, a 562 base region of the ribosomal protein S7 (RPS7) cDNA was amplified using forward primer 5'-ATGGTTTTCGGATCAAAGG-3' Tm = 61.3°C, and reverse primer 5'-GGAATTCGAACGTAACGTCAC-3' Tm = 63.2°C. The expected genomic size is 5.5 kb. Negative control reactions without the use of reverse transcriptase were performed to test for genomic DNA contamination.

### Embryonic injection and luciferase assay

*Ae. aegypti Liverpool *strain embryos were injected with pGL3-basic firefly luciferase reporter plasmid (Promega) containing 1042 bp of the 5' UTR and upstream genomic sequence of the *AaKLC2.1 *early transient zygotic gene (gene ID: AAEL011410). For each injection group, an average of 45 embryos were injected at approximately 1 hr after deposition with 0.30 ug/ul of plasmid either with or without (negative control) insert. A *Renilla *luciferase reporter pRL-null (Promega) with the *D. melanogaster *actin promoter was used as an injection control at 0.15 ug/ul. 1× Injection Buffer used was 5 mM KCl 0.1 mM NaH2PO4 pH 7.2, sterile filtered. Embryos were homogenized in Cell Culture Lysis Buffer at 3 hr and 5 hr after deposition and assayed for luciferase activity using the Luciferase Assay System (Promega) and a Glomax 20/20 luminometer (Promega). Samples were read for 10 seconds. Injection needles were pulled from World Precision Instruments Kwik-Fil Borosilicate Glass Capillaries (item # 1B100F-4) on a Sutter Instrument P-2000 Micropipette Puller at the following settings: Heat = 270, Fil = 3, Vel = 37, Del = 250, Pul = 134. Injections were performed using an Eppendorf FemtoJet allowing injection solution to free-flow through the needle with the compensation pressure (Pc) set at ~3750 hPa. Injection Pressure setting (Pi) not used. The needle is held in a World Precision Instruments Micromanipulator (model M3301L) http://www.wpiinc.com. Microscope used was a Leica CM E.

## Authors' contributions

JB performed sequence analysis, phylogenetic inference, RT-PCR, luciferase assays, and wrote the manuscript. ZT conceived and oversaw the project, planned the embryonic transcriptome sequencing, analyzed the transcriptome data, and revised the manuscript. All authors read and approved the final manuscript.

## Author's Information

JB is a Research Scientist at Virginia Polytechnic Institute and State University

ZT is a Professor at Virginia Polytechnic Institute and State University

## Supplementary Material

Additional file 1 **Summary of Illumina RNA sequence data**. Click here for file

Additional file 2 **FASTA file of Illumina reads that matched the three KLC transcripts**. Click here for file

Additional file 3 **FASTA file of amino acid sequences used in this study**. Click here for file

Additional file 4 **FASTA file of amino acid sequence alignment used for phylogenetic inference**. Click here for file
