# Supplementary material for: Evolutionary analysis of the kinesin light chain genes in the yellow fever mosquito Aedes aegypti: gene duplication as a source for novel early zygotic genes
Source: BMC Evol Biol. 2010 Jul 8;10:206. doi: 10.1186/1471-2148-10-206 (PMC2927918; doi:10.1186/1471-2148-10-206)
Supplement: Additional file 2 — FASTA file of Illumina reads that matched the three KLC transcripts. [file 1471-2148-10-206-S2.DOC]

**Fasta file of Illumina reads that matched the three KLC transcripts**

The convention for names of these Illumina reads is: Sample name followed by a unique identification number followed by the number of reads that had the same sequence. For example, JTC_1270893_1 means there is 1 read in sample JTC that had the particular fasta sequence and 1270893 is the unique identification number of the sequence. JTA, 0-2 hr embryo sample; JTB, 2-4 hr embryo; JTC, 4-8 hr embryo; JTD, 8-12 hr embryo.

**1) Illumina reads that matched AAEL011410**

>JTC_1270893_1

GGAAGCGCTACGTACTGAGCTGCAGTCTATCCG

>JTC_673531_1

TGATGATCCAGCATTTGACCTATTCCCCGAAGA

>JTC_1399824_1

GCCTTATTATCTCACCTGAAAAGCATCGACGTT

>JTB_1765742_1

CTGCGAATTCCATCTGTCTCTTTTCCTCTTCGA

>JTB_3090350_2

CCGTTGTGTAAGCAAGCCCTAGAAGATCTTGAA

>JTB_908993_1

GTTTCAAACAGCATGCAGCTAGATTACACTTGG

>JTC_3357275_1

TGAACATCCAGATGTGGCAACAATGTTAAGCCT

>JTC_5165093_1

AGATGGAATTCGCAGCATCGATCAAGAAATACG

>JTC_3419758_1

TCATCACCGAACTTCTCGTCGAATTCTACACCT

>JTB_1774651_1

CTGATCTCGGTAGACCATGGTCAGGATGCTTAA

>JTB_198396_2

TGTCGACTTGCTAAGTCGTCTTTCTGCAATTTC

>JTB_4445128_1

CTTGAACGTGAGAATGGCCGTGAACATCCAGAT

>JTC_1034702_1

GTCATCCATCGGTGGCAGCGGCTTTAAATAATT

>JTC_422142_1

TTTCTTGATCGATGCTGCGAATTCCATCTGTCT

>JTB_4294730_1

GATCTCGGTAGACCATGGTCAGGATGCTTAACA

>JTC_3446745_1

TAGAATTCGACGAGAAGTTCGGTGATGATCCAG

>JTC_3114570_1

TTGCTCCGGAACTGACCGTGTTTCAGCTTGGAT

>JTB_937550_1

GTTGCATCAAAGATTGGGCAACTTTGACGTGGC

>JTC_2779749_4

TGAACATCCAGATGTGGCAACAATGTTAAGCAT

>JTC_958735_1

GTGTAAGCAAGCCCTAGAAGATCTTGAACGTGA

>JTC_745619_1

TCGGGGAATAGGTCAAATGCTGGATCATCACCG

>JTC_3009268_2

CAACTGTTTTGCCACATCGGGATGATATCGGCC

>JTC_3157649_1

TTCATATGTTTGATGGCTTCTGGTAGGTTATTC

>JTB_3296453_1

TGTTGGTCAAAACGCAGGATAAGGAATTCGATG

>JTC_4402311_1

CTCGGTAGACCATGGTCAGGATGCTTAACATTG

>JTB_2989760_2

GTCAAAAGTATACTAAAGCATATGGCCTCGTTG

>JTC_566855_1

TGTAAGCAAGCCCTAGAAGATCTTGAACGTGAG

>JTB_4482891_1

CTTACACAACGGTACTGCGACTTCATATCTTCC

>JTC_3348887_1

TGAAGTCGCAGTACCGTTGTGTAAGCAAGCCCC

>JTC_421171_1

TTTGAAACTCCGTAAGTACAAAGAAGCTGACCA

>JTC_884815_1

GTTGTGTAAGCAAGCCCTAGAAGATCTTGAACG

>JTB_4351022_1

GAAGTCGCAGTACCGTTGTGTAAGCAAGCCCTA

>JTC_3286554_1

TGCGTTTTGACCAACACGTCTCTTAACAACTGG

>JTC_1721042_1

CTGCAATTTCATCTTCGGGGAATAGGTCAAATG

>JTC_4328813_1

CTTACACAACGGTACTGCGACTTCATATCTTCC

>JTB_199348_2

TGTAAGCAAGCCCTAGAAGATCTTGAACGTGAG

>JTB_4073222_1

GGAACTGACCGTGTTTCAGCTTGGATGCCACGT

>JTC_1738676_1

CTCTTGCATAAAGATTCCGCTTCCTTGTAGTTA

>JTC_4315280_1

CTTCATTCATATGTTTGATGGCTTCTGGTAGGT

>JTC_5006733_1

CAACGGTCAAAAGTATACTAAAGCATATGGCCT

>JTC_3984250_1

GCTGCATGCTGTTTGAAACTCCGTAAGTACAAA

>JTC_214248_2

TAAAGATTCCGCTTCCTTGTAGTTACCGTTCTT

>JTC_2647817_1

AAGCGGAATCTTTATGCAAGAGAGCTTTGGCTA

>JTC_5200518_1

AGAAAGACGACTTAGCAAGTCGACACCAACACC

>JTC_2167165_1

CCTAGCTGTGATTCGAAAATTTCCAACGCTCTT

>JTC_3973921_1

GCTGTTTCGTGCCATGCTCCACTTTTACCGTCA

>JTC_1405977_1

GCCGTGAACATCCAGATGTGGCAACAATGTTAA

>JTC_507725_1

TTCATATCTCCCCTTTGAGGCGTAGCTTAATGC

>JTC_3744052_1

GGTCAGCTTCTTTGTACTTACGGAGTTTCAAAC

>JTB_5296596_1

AGTCAGCAGCAACTGCTGGAAGCTCAACTTACA

>JTC_3433040_1

TATGCCCAAGGCTTCATTCATATGTTTGATGGC

>JTC_4516433_1

CGGTATCATCCCGATGTGGCAAAACAGTTGAAC

>JTC_3273079_1

TGGAACTGTACATTCGAAGAGCGTTGGAAATTT

>JTC_1014150_1

GTCGCAGTACCGTTGTGTAAGCAAGCCCTAGCA

>JTC_3248682_1

TGGCCGTTTTGTATGGAAAGAACGGTAACTACA

>JTB_768557_1

TCTCCCAATCCTAGTTCGATGTTCTGCGCATTT

>JTC_2419556_1

CAAAAGTATACTAAAGCATATGGCCTCGTTGCA

>JTC_5265013_1

AAGATATGAAGTCGCAGTACCGTTGTGTAAGCA

>JTC_3164840_1

TTATTCTGATCTCGGTAGACCATGGTCAGGATG

>JTB_2462050_1

CAAAAGTATACTAAAGCATATGGCCTCGTTGCA

>JTC_4783413_1

CCTGCGCATTTCGATTGATGACTTCTTCCTTCT

>JTB_507686_1

TTCTGATCTCGGTAGACCATGGTCAGGATGCTT

>JTB_2656703_1

ACCTATTCCCCGAAGATGAAATTGCAGAAAGAC

>JTC_4671258_1

CGCAGTACCGTTGTGTAAGCAAGCCCTAGAAGA

>JTC_3385581_1

TCGTGAAAACGTTCTTGGCCGATATCANCCCGA

>JTC_283251_2

CTTTGCTTGACTGTCTTCATTTTGGAGAGCAGA

>JTC_4912845_1

CATCCACAATATCCAAAATCTCCTGTTTGCTCC

>JTC_3403375_1

TCCTTATCCTGCGTTTTGACCAACACGTCTCTT

>JTC_4718180_1

CGACTTGCTAAGTCGTCTTTCTGCAATTTCATC

>JTB_5414442_1

AATTTTCGAATCACAGCTAGGTGCTAACGATCC

>JTC_2660231_1

AACTGCTGGAAGCTCAACTTAAAATAGTCCAGC

>JTC_113711_3

TTCTGATCTCGGTAGACCATGGTCAGGATGCTT

>JTC_2947359_2

GAAACAGCAGTGGTTCACTCTCCAACGGTCAAA

>JTC_3067068_1

TTTGCCACATCGGGATGATATCGGCCAAGAACG

>JTB_821036_1

TCAAAACCAAGTGTAATCTAGCTGCATGCTGTT

>JTB_3189551_1

TTTCGATTGATGACTTCTTCCTTCTCCGGATTG

>JTC_2466266_1

ATTACACTTGGTTTTGATGGCATTGGGATCGAT

>JTC_252335_2

GGAATTCGATGAGACACAGAATGGTGACAGTGA

>JTC_1814104_1

CGTTTTCACGATTAGCCAAAGCTCTCTTGCATA

>JTB_2391798_1

CACGGTCAGTTCCGGAGCAAACAGGAGATTTTG

>JTC_2123462_1

CGAAGAGCGTTGGAAATTTTCGAATCACAGCTA

>JTC_4766634_1

CGAAACAGCAGTGGTTCACTCTCCAACGGTCAC

>JTB_779348_1

TCGGGATGATATCGGCCAAGAACGTTTTCACGC

>JTC_3949200_1

GGAAAGAACGGTAACTACAAGGAAGCGGAATCT

>JTB_2030368_1

CGGAACTGACCGTGTTTCAGCTTGGATGCCACG

>JTC_480018_1

TTCTGGTAGGTTATTCTGATCTCGGTAGACCAT

>JTB_4206882_1

GCCGTGAACATCCAGATGTGGCAACAATGTTAA

>JTB_4593344_1

CTCAACTTAAAATAGTCCAGCTCGAAGAGGAAA

>JTC_789517_1

TATGGGTTCGCTGTTTGGGTGAGTGTCATCCAT

>JTC_3502450_1

GTTTGATGGCTTCTGGTAGGTTATTCTGATCTC

>JTC_4990314_1

CAATATCCAAAATCTCCTGTTTGCTCCGGAACT

>JTB_4446225_1

CTTCTTTGTACTTACGGAGTTTCAAACAGCATG

>JTC_3105174_1

TTGGTGTCGACTTGCTAAGTCGTCTTTCTGCAA

>JTC_1867576_1

CGGTGATGATCCAGCATTTGACCTATTCCCCGC

>JTC_1198194_1

GGATTGGGAGAAGCTCAGCTGTTGGTCGCCTTA

>JTC_380075_2

AAACAGCAGTGGTTCACTCTCCAACGGTCAAAA

>JTC_3846156_1

GGATTGGGAGAAGCTCAGCTGTTGGTCGCCTTA

>JTC_5160395_1

AGCAAGCCCTAGAAGATCTTGAACGTGAGAATG

>JTB_4320889_1

GAGAAGCTCAGCTGTTGGTCGCCTTATTATCTT

>JTC_3587639_1

GTTCACTCTCCAACGGTCAAAAGTATACTAAAG

>JTC_865491_1

GTTTCGTGCCATGCTCCACTTTTACCGTAAGAA

>JTC_4686137_1

CGATTATGAAGTGTCTCCACGTCTAAAGACTAT

>JTC_4938129_1

CAGCATGCAGCTAGATTACACTTGGTTTTGATG

>JTC_2386500_1

CAAGAACGTTTTCACGATTAGCCAAAGCTCTCT

>JTC_4696810_1

CGATCCCAATGCCATCAAAACCAAGTGTAATCT

>JTB_3114200_2

ATTTGGCCGTTTTGTATGGAAAGAACGGTAACT

>JTC_2374811_1

CAATATCCAAAATCTCCTGTTTGCTCCGGAACT

>JTB_5061714_1

CATTGTTGCCACATCTGGATGTTCACGGCCCTT

>JTB_441796_1

TTTGACTTGTATGCGAAGCGTTTGCCGTTCCCC

>JTC_2074658_1

CGAGGTTGTTCAACTGTTTTGCCACATCGGGAT

>JTC_1798526_1

CTAGTTCGATGTTCTGCGCATTTCGATTGATGA

>JTC_4517525_1

CGGTAGACCATGGTCAGGATGCTTAACATTGTT

>JTB_5377578_1

ACTTCATCCCGTAGCCAGGAGGTTTCCTGACAC

>JTC_970953_1

GTGGAACTGTACATTCGAAGAGCGTTGGAAATT

>JTC_5240734_1

AATTTGGCCGTTTTGTATGGAAAGAACGGTAAC

>JTC_2653832_1

AAGACAGTCAGCAGCAACTGCTGGAAGCTCAAC

>JTB_2974463_2

GTTGGTCAAAACGCAGGATAAGGAATTCGATGA

>JTC_2690379_1

AAACAGCAGTGGTTCACTCTCCAACGGTCAAAG

>JTC_2292530_1

CATCGAACTAGGATTGGGAGAAGCTCAGCTGAG

>JTC_1206494_1

GGATGATATCGGCCAAGAACGTTTTCACGATTA

>JTC_4730798_1

CGACCGTTGGAGAGTGAACCACTGCTGTTTCGT

>JTB_2607972_1

AGACTCTCGTCGGTCATCAGCAACGTTGAGTGT

>JTB_2519963_1

ATGACACTCACCCAAACAGCGAACCCATATGCC

>JTB_2304806_1

CCAGGCTTTGCTTGACTGTCTTCATTTTGGAGA

>JTB_948490_1

GTTCTTGGCCGATATCATCCCGATGTGGCAAAA

>JTB_2397467_1

CACGAAACAGCAGTGGTTCACTCTCCAACGGTC

>JTC_3921159_1

GGAAGCGGAATCTTTATGCAAGAGAGCTTTGGC

>JTC_5132377_1

AGGTTATTCTGATCTCGGTAGACCATGGTCAGG

>JTB_3883388_1

GGTCAAAACGCAGGATAAGGAATTCGATGAGGT

>JTC_2154988_1

CCTGCGTTTTGACCAACACGTCTCTTAACAACT

>JTB_1771093_1

CTGCAATTTCATCTTCGGGGAATAGGTCAAATG

>JTC_2054851_1

CGATTATGAAGTGTCTCCACGTCTAAAGACTAT

>JTC_2995682_2

CCGGAACTGACCGTGTTTCAGCTTGGATGCCAC

>JTC_2609985_1

ACGAAACAGCAGTGGTTCACTCTCCAACGGTCA

>JTB_3481050_1

TGAACATCCAGATGTGGCAACAATGTTAAGCAT

>JTB_5364396_1

AGAACGTTTTCACGATTAGCCAAAGCTCTCTTG

>JTC_2308226_1

CAGGCTTTGCTTGACTGTCTTCATTTTGGAGAG

>JTB_302003_2

CTTCTTTGTACTTACGGAGTTTCAAACAGCATG

>JTC_3783424_1

GGGATGCTTAACATTGTTGCCACATCTGGATGT

>JTC_252330_2

GGAATTCGCAGCATCGATCAAGAAATACGACGA

>JTC_87965_4

GTAAAAGACAGTCAGCAGCAACTGCTGGAAGCT

>JTC_5262179_1

AAGCCCTAGAAGATCTTGAACGTGAGAATGGCC

>JTC_1353229_1

GCGTAGCTTAATGCCAAGTTTTGAATAGTCTTT

>JTC_3462174_1

TAACATTGTTGCCACATCTGGATGTTCACGGCC

>JTB_2263805_1

CCGATATCATCCCGATGTGGCAAAACAGTTGAA

>JTC_1436406_1

GCCAAAATCAAGGAAAGCACGGTGAGGTGGAGC

>JTC_2482832_1

ATCGTTAGCACCTAGCTGTGATTCGAAAATTTC

>JTB_858589_1

TAAAGATTCCGCTTCCTTGTAGTTACCGTTCTT

>JTB_3237057_1

TTGAACAACCTCGCATTATTATGCCAAAATCAA

>JTC_410614_1

TTTGGGTGAGTGTCATCCATCGGTGGCAGCGGC

>JTB_1043739_1

GTCGTATTTCTTGATCGATGCTGCGAATTCCAT

>JTC_721173_1

TCTTGATCGATGCTGCGAATTCCATCTGTCTCT

>JTB_3023585_2

GCCATATGCTTTAGTATACTTTTGACCGTTGGA

>JTC_1217088_1

GGAGTTTCAAACAGCATGCAGCTAGATTACACT

>JTB_1864463_1

CGTTGTGTAAGCAAGCCCTAGAAGATCTTGAAC

>JTC_3832074_1

GGCCAAGAACGTTTTCACGATTAGCCAAAGCTC

>JTC_2920910_2

GGAGAGTGAACCACTGCTGTTTCGTGCCATGCT

>JTC_5173570_1

AGAGCGTTGGAAATTTTCGAATCACAGCTAGGT

>JTB_2343906_1

CATAAAGATTCCGCTTCCTTGTAGTTACCGTTC

>JTC_3607073_1

GTGTAAGCAAGCCCTAGAAGATCTTGAACGTGA

>JTB_4032423_1

GGACGGTGTTGGTGTCGACTTGCTAAGTCGTCT

>JTB_5195843_1

CAAAAGTATACTAAAGCATATGGCCTCGTTGCA

>JTB_4067262_1

GGAAGATCTTGAACGTGAGAATGGCCGTGAACA

>JTB_4570958_1

CTCCTGTTTGCTCCGGAACTGACCGTGTTTCAG

>JTC_2513047_1

AGTCGCAGTACCGTTGTGTAAGCAAGCCCTGGA

>JTC_2812727_3

GTTTTGTATGGAAAGAACGGTAACTACAAGGAA

>JTC_3481022_1

GTTTTGATGGCATTGGGATCGTTAGCACCTAGA

>JTC_5267518_1

AAGACAGTCAGCAGCAACTGCTGGAAGCTCAAC

>JTB_2602269_1

AGATAATAAGGCGACCAACAGCTGAGCTTCTCC

>JTB_828449_1

TATCGGCCAAGAACGTTTTCACGATTAGCCAAA

>JTB_2721373_1

AAATGCTGGATCATCACCGAACTTCTCGTCGAA

>JTC_4537362_1

CGGGGAATAGGTCAAATGCTGGATCATCACCGA

>JTC_4194229_1

GAAGTTCGGTGATGATCCAGCATTTGACCTATT

>JTC_1630623_1

CTTGCATAAAGATTCCGCTTCCTTGTAGTTACC

>JTC_579137_1

TGGTCAGGATGCTTAACATTGTTGCCACATCTG

>JTC_2871193_2

TGTCGACTTGCTAAGTCGTCTTTCTGCAATTTC

>JTC_759973_1

TCCTTATCCTGCGTTTTGACCAACACGTCTCTT

>JTC_2410595_1

CAAAGATTGGGCAACTTTGACGTGGCATCCAAG

>JTB_3855647_1

GGTTGTTCAACTGTTTTGCCACATCGGGATGAT

>JTC_4362435_1

CTGCTCTCCAAAATGAAGACAGTCAAGCAAAGC

>JTB_4789486_1

CGCTGCCACCGATGGATGACACTCACCCAAACA

>JTB_5236014_1

ATTCGATGAGACACAGAATGGTGACAGTGAATC

>JTC_3719384_1

GGTTGTTCAACTGTTTTGCCACATCGGGATGCT

>JTC_3360702_1

TGAAAGACAGTCAGCAGCAACTGCTGGAAGCTC

>JTC_1754194_1

CTCGGTAGACCATGGTCAGGATGCTTAACATTG

>JTC_2777305_4

TTCTGATCTCGGTAGACCATGGTCAGGATGCTT

>JTC_1004623_1

GTCGTATTTCTTGATCGATGCTGCGAATTCCCT

>JTB_2959913_2

TCTCGGTAGACCATGGTCAGGATGCTTAACATT

>JTB_3472238_1

TGAAGTCGCAGTACCGTTGTGTAAGCAAGCCCT

>JTC_4840246_1

CCGATGGATGACACTCACCCAAACAGCGAACCC

>JTB_2873497_3

GTTTTGACCAACACGTCTCTTAACAACTGGTCA

>JTC_1953624_1

CGGAGTTTCAAACAGCATGCAGCTAGATTACAC

>JTC_1632618_1

CTTGATCGATGCTGCGAATTCCATCTGTCTCTT

>JTC_3413198_1

TCCCGATGTGGCAAAACAGTTGAACAACCTCGC

>JTB_1706813_1

CTTCCTTCTCCGGATTGGAGATGGGATCTGCAC

>JTB_1622169_1

GAAAGACAGTCAGCAGCAACTGCTGGAAGCTCC

>JTC_1086185_1

GGTGAGTGTCATCCATCGGTGGCAGCGGCTTTA

>JTC_1014151_1

GTCGCAGTACCGTTGTGTAAGCAAGCCCTAGAA

>JTC_603721_1

TGGCAGCGGCTTTAAATAATTTGGCCGTTTTGT

>JTB_425195_1

TTTTACTTCATCCCGTAGCCAGGAGGTTTCCTG

>JTB_4923304_1

CGAAACAGCAGTGGTTCACTCTCCAACGGTCAC

>JTC_835256_1

GTTTTGTATGGAAAGAACGGTAACTACAAGGAA

>JTC_1055275_1

GTACCGTTGTGTAAGCAAGCCCTAGAAGATCTT

>JTB_3754434_1

GTGCCATGCTCCACTTTTACCGTAAGAAGTGTT

>JTC_1611637_1

CTTTAGTATACTTTTGACCGTTGGAGAGTGAAC

>JTC_1058596_1

GTAATCTAGCTGCATGCTGTTTGAAACTCCGTA

>JTB_4306660_1

GAGGTTGTTCAACTGTTTTGCCACATCGGGATG

>JTC_2060798_1

CGATGAGACACAGAATGGTGACAGTGAATCAAT

>JTB_1300252_1

GGAATTCGCAGCATCGATCAAGAAATACGACGA

>JTC_5036527_1

CAAAACCAAGTGTAATCTAGCTGCATGCTGTTT

>JTC_3013362_2

ATTTGGCCGTTTTGTATGGAAAGAACGGTAACT

>JTB_1365973_1

GCTGTTGGTCGCCTTATTATCTCACCTGAAAAG

>JTC_4138434_1

GATGGATGACACTCACCCAAACAGCGAACCCAT

>JTB_605541_1

TGGTGACAGTGAATCAATTAACGTCGATAACAC

>JTC_5179968_1

AGACCATGGTCAGGATGCTTAACATTGTTGCCA

>JTC_3028151_2

AAGACAGTCAGCAGCAACTGCTGGAAGCTCCAC

>JTC_4213501_1

GAAATTTTCGAATCACAGCTAGGTGCTAACGAT

>JTC_2629769_1

AATTCGATGAGACACAGAATGGTGACAGTGAAT

>JTC_703700_1

TGAAGTGTCTCCACGTCTAAAGACTATTCAAAA

>JTB_3093264_2

CCGCTTCCTTGTAGTTACCGTTCTTTCCATACA

>JTC_2887096_2

TATCGGCCAAGAACGTTTTCACGATTAGCCAAA

>JTC_3885856_1

GGACTTTGTCAGGAAACCTCCTGGCTACGGGAC

>JTC_3692792_1

GTCAAATGCTGGATCATCACCGAACTTCTCGTC

>JTB_795796_1

TCCTGCGTTTTGACCAACACGTCTCTTAACAAC

>JTB_1616197_1

GAAATGCGCAGAACATCGAACTAGGATTGGGAG

>JTC_2486472_1

ATCGATCAAGAAATACGACGACTTTTTGGAAGG

>JTC_1824495_1

CGTGTTTCAGCTTGGATGCCACGTCAAAGTTGC

>JTB_5245211_1

ATTACACTTGGTTTTGATGGCATTGGGATCGAT

>JTB_225902_2

GTTTGCTCCGGAACTGACCGTGTTTCAGCTTGG

>JTC_2690378_1

AAACAGCAGTGGTTCACTCTCCAACGGTCACCA

>JTB_1276708_1

GGAGAGTGAACCACTGCTGTTTCGTGCCATGCT

>JTC_4872928_1

CCCAATGCCATCAAAACCAAGTGTAATCTAGCT

>JTB_3391921_1

TGGAACTGTACATTCGAAGAGCGTTGGAAATTT

>JTC_4102006_1

GCAGACTCTCGTCGGTCATCAGCAACGTTGATT

>JTB_3331975_1

TGTAAGCAAGCCCTAGAAGATCTTGAACGTGAG

>JTC_4716276_1

CGAGAAGTTCGGTGATGATCCAGCATTTGACCT

>JTC_958660_1

GTGTAATCTAGCTGCATGCTGTTTGAAACTCCG

>JTC_2482101_1

ATCTAGCTGCATGCTGTTTGAAACTCCGTAAGT

>JTC_2543314_1

AGCATCGATCAAGAAATACGACGACTTTTTGGA

>JTC_3704051_1

GTACCGTTGTGTAAGCAAGCCCTAGAAGATCTT

>JTC_940086_1

GTTCACTCTCCAACGGTCAAAAGTATACTAACG

>JTC_3495992_1

GTTTGGGTGAGTGTCATCCATCGGTGGCAGCGG

>JTC_5080655_1

ATTAGCCAAAGCTCTCTTGCATAAAGATTCCGC

>JTB_1691164_1

CTTCTTCCTTCTCCGGATTGGAGATGGGATCTG

>JTC_3376294_1

TCTCTTAACAACTGGTCAGCTTCTTTGTACTTA

>JTC_4503874_1

CGGTGTTGGTGTCGACTTGCTAAGTCGTCTTTC

>JTC_3423079_1

TCACGGCCATTCTCACGTTCAAGATCTTCTATG

>JTC_987623_1

GTGAACCACTGCTGTTTCGTGCCATGCTCCACT

>JTB_1100523_1

GTAAGTACAAAGAAGCTGACCAGTTGTTAAGAG

>JTB_1780774_1

CTGACCAGTTGTTAAGAGACGTGTTGGTCAAAA

>JTB_4003306_1

GGATCATCACCGAACTTCTCGTCGAATTCTACA

>JTB_4529130_1

CTGATCTCGGTAGACCATGGTCAGGATGCTTAA

>JTB_541080_1

TTCAACTGTTTTGCCACATCGGGATGATATCGG

>JTC_3062848_1

TTTGGCCGTTTTGTATGGAAAGAACGGTAATTA

>JTC_3917451_1

GGAAGGTGTAGAATTCGACGAGAAGTTCGGTGA

>JTC_1473158_1

GCAAAACAGTTGAACAACCTCGCATTATTATGC

>JTC_3889461_1

GGACGGTGTTGGTGTCGACTTGCTAAGTCGTCT

>JTC_4796392_1

CCGTTTTGTATGGAAAGAACGGTAACTACAAGG

>JTC_3864108_1

GGATAATGCTGGGACGGTGTTGGTGTCGACTTT

>JTC_4899113_1

CCAAAATCTCCTGTTTGCTCCGGAACTGACCGT

>JTB_4601301_1

CTAGATTACACTTGGTTTTGATGGCATTGGGAT

>JTC_493740_1

TTCGATGAGACACAGAATGGTGACAGTGAATCA

>JTC_2568864_1

AGAATTCGACGAGAAGTTCGGTGATGATCCAGC

>JTB_1533674_1

GATTATGAAGTGTCTCCACGTCTAAAGACTATT

>JTC_1605128_1

CTTTCTGCAATTTCATCTTCGGGGAATAGGTCA

>JTB_4484201_1

CTTAACATTGTTGCCACATCTGGATGTTCACGG

>JTC_1840555_1

CGTCGATGCTTTTCAGGTGAGATAATAAGGCGA

>JTB_3881246_1

GGTCAGGATGCTTAACATTGTTGCCACATCTGG

>JTC_1485513_1

GATGGATGACACTCACCCAAACAGCGAACCCAT

>JTB_5492088_1

AAAAGACAGTCAGCAGCAACTGCTGGAAGCTCA

>JTB_2156751_1

CGAATTCCTTATCCTGCGTTTTGACCAACACGT

>JTC_2489784_1

ATCCGATACGGCATCTCCAGTACAGATCCCATC

>JTC_3446495_1

TAGACCATGGTCAGGATGCTTAACATTGTTTCC

>JTC_1637930_1

CTTCTTTGTACTTACGGAGTTTCAAACAGCATG

>JTC_239582_2

GGTCAGGATGCTTAACATTGTTGCCACATCTGG

>JTC_3163745_1

TTCAAACAGCATGCAGCTAGATTACACTTGGTT

>JTC_3743996_1

GGTCAGGATGCTTAACATTGTTGCCACATCTGG

>JTC_5070065_1

ATTCTGATCTCGGTAGACCATGGTCAGGATGCT

>JTB_3464852_1

TGACCATGGTCTACCGAGATCAGAATAACCTCA

>JTC_545955_1

TGTGGAGCATGGCACGAAACAGCAGTGGTTCAC

>JTC_4214391_1

GAAATTGCAGAAAGACGACTTAGCAAGTCGACA

>JTC_2145099_1

CCTTGATTTTGGCATAATAATGCGAGGTTGTTC

>JTC_3377686_1

TCTCGGTAGACCATGGTCAGGATGCTTAACATT

>JTC_3628526_1

GTGAGGTGGAACTGTACATTCGAAGAGCGTTGG

>JTB_4645405_1

CGTATTTCTTGATCGATGCTGCGAATTCCATCT

>JTB_70872_5

CGGAATCTTTATGCAAGAGAGCTTTGGCTAATC

>JTC_2388869_1

CAACTGGTCAGCTTCTTTGTACTTACGGAGTTT

>JTB_2926415_3

AATAGGTCAAATGCTGGATCATCACCGAACTTC

>JTB_1365206_1

GCTTAACATTGTTGCCACATCTGGATGTTCACG

>JTC_4713924_1

CGAGATCAGAATAACCTACCAGAAGCCATCAAA

>JTB_873251_1

GTTTTGACCAACACGTCTCTTAACAACTGGTCA

>JTB_1137343_1

GGTCAAAACGCAGGATAAGGAATTCGATGAGGT

>JTB_2570840_1

AGGATGCTTAACATTGTTGCCACATCTGGATGT

>JTB_4657388_1

CGGTGTTGGTGTCGACTTGCTAAGTCGTCTTTC

>JTB_4486016_1

CTGTTGGTCGCCTTATTATCTCACCTGAAAAGC

>JTB_2531951_1

ATCGAATTCCTTATCCTGCGTTTTGACCAACAC

>JTC_205617_2

TGAACATCCAGATGTGGCAACAATGTTAAGCAT

>JTC_2846394_3

CCGTTGTGTAAGCAAGCCCTAGAAGATCTTGAA

>JTB_1914234_1

CGGTGATGATCCAGCATTTGACCTATTCCCCGA

>JTC_2361230_1

CACAGAATGGTGACAGTGAATCAATTAACGTCG

>JTB_3381630_1

TGGAGAGTGAACCACTGCTGTTTCGTGCCATGC

>JTC_1553240_1

GAACTGTACATTCGAAGAGCGTTGGAAATTTTC

>JTB_3658420_1

GTTGTTCAACTGTTTTGCCACATCGGGATGATA

>JTC_1240349_1

GGACGGTGTTGGTGTCGACTTGCTAAGTCGCCT

>JTC_471841_1

TTGACTTGTATGCGAAGCGTTTGCCGTTCAACG

>JTC_5204504_1

ACTTTTTGGAAGGTGTAGAATTCGACGAGACGT

>JTB_5340982_1

AGATAATAAGGCGACCAACAGCTGAGCTTCTCC

>JTB_1578536_1

GACCTATTCCCCGAAGATGAAATTGCAGAAAGA

>JTB_3698770_1

GTTCGGTGATGATCCAGCATTTGACCTATTCCC

>JTC_4207885_1

GAACGGCAAACGCTTCGCATACAAGTCAAAGGA

>JTC_149442_3

CTGATCTCGGTAGACCATGGTCAGGATGCTTAA

>JTB_462290_1

TTTCACGATTAGCCAAAGCTCTCTTGCATAAAG

>JTB_3542984_1

TCCAGGCTTTGCTTGACTGTCTTCATTTTGGAG

>JTB_1706814_1

CTTCCTTCTCCGGATTGGAGATGGGATCTGCAA

>JTC_2113113_1

CGAATGTACAGTTCCACCTCACCGTGCTTTCCT

>JTB_4234891_1

GCCAAGTGTAATCTAGCTGCATGCTGTTTGAAA

>JTB_1626969_1

GAAACAGCAGTGGTTCACTCTCCAACGGTCAAA

>JTB_808131_1

TCCAGATGTGGCAACAATGTTAAGCATCCTGAC

>JTC_4741290_1

CGAATTCCTTATCCTGCGTTTTGACCAACACGT

>JTC_4755781_1

CGAACTAGGATTGGGAGAAGCTCAGCTGTTGGC

>JTB_4052910_1

GGAATCTTTATGCAAGAGAGCTTTGGCTAATCG

>JTC_3774019_1

GGGGAATAGGTCAAATGCTGGATCATCACCGAA

>JTB_995744_1

GTGTAAGCAAGCCCTAGAAGATCTTGAACGCGA

>JTB_4306631_1

GAGGTTTCCTGACAAAGTCCTTTGACTTGTATG

>JTB_3080713_2

CGATATCATCCCGATGTGGCAAAACAGTTGAAC

>JTC_1320171_1

GCTTAACATTGTTGCCACATCTGGATGTTCACG

>JTC_2651381_1

AAGATATGAAGTCGCAGTACCGTTGTGTAAGCA

>JTC_4078009_1

GCCATATGCTTTAGTATACTTTTGACCGTTGGA

>JTC_3571036_1

GTTCGCTGTTTGGGTGAGTGTCATCCATCGGTG

>JTC_361808_2

ATTCGATGAGACACAGAATGGTGACAGTGAATC

>JTC_2164892_1

CCTCATCGAATTCCTTATCCTGCGTTTTGACCA

>JTC_5156733_1

AGCCAGGAGGTTTCCTGACAAAGTCCTTTGACT

>JTC_3022179_2

AGACAGTCAGCAGCAACTGCTGGAAGCTCAACT

>JTB_3986363_1

GGCAAAACAGTTGAACAACCTCGCATTATTATG

>JTB_1315437_1

GGAAGCGCTACGTACTGAGCTGCAGTCTATCCG

>JTC_4219296_1

GAAATACGACGACTTTTTGGAAGGTGTAGAATT

>JTC_1406940_1

GCCGTATCGGATAGACTGCAGCTCAGTACGTAG

>JTB_706407_1

TGATGATCCAGCATTTGACCTATTCCCCGACGC

>JTB_4375751_1

GAAATACGACGACTTTTTGGAAGGTGTAGAATT

>JTB_744780_1

TGAACCACTGCTGTTTCGTGCCATGCTCCACTT

>JTB_3526575_1

TCGACGAGAAGTTCGGTGATGATCCAGCATTTG

>JTC_3467399_1

TAAAAGACAGTCAGCAGCAACTGCTGGAAGCTC

>JTC_2948790_2

CTTTTTGGAAGGTGTAGAATTCGACGAGAAGTT

>JTB_4584310_1

CTCCAATCCGGAGAAGGAAGAAGTCATCAATCG

>JTB_3106493_2

CACAGAATGGTGACAGTGAATCAATTAACGTCG

>JTC_2040497_1

CGCAGGATAAGGAATTCGATGAGACACAGAATG

>JTC_2170808_1

CCGTGTTTCAGCTTGGATGCCACGTCAAAGTTG

>JTC_5243631_1

AATTCGCAGCATCGATCAAGAAATACGACGACT

>JTC_4295081_1

CTTCTGGTAGGTTATTCTGATCTCGGTAGACCA

>JTC_3343110_1

TGACAGTGAATCAATTAACGTCGATAACACTTC

>JTB_2880633_3

GTCAAATGCTGGATCATCACCGAACTTCTCGTC

>JTC_3973424_1

GCTTAACATTGTTGCCACATCTGGATGTTCAAG

>JTC_4987669_1

CAATCTTTGATGCAACGAGGCCATATGCTTTAG

>JTB_4636613_1

CGTCGGTGGCAGCGGCTTTAAATAATTTGGCCG

>JTC_3685670_1

GTCAGCAGCAACTGCTGGAAGCTCAACTTAAAA

>JTC_1631311_1

CTTGATTTTGGCATAATAATGCGAGGTTGTTCA

>JTC_4285440_1

CTTGATCGATGCTGCGAATTCCATCTGTCTCTT

>JTB_2317456_1

CCAATCCGGAGAAGGAAGAAGTCATCAATCGAA

>JTC_3977785_1

GCTGGTAGGTTATTCTGATCTCGGTAGACCATG

>JTC_3719385_1

GGTTGTTCAACTGTTTTGCCACATCGGGATGAT

>JTC_1607519_1

CTTTCCTTGATTTTGGCATAATAATGCGAGGTT

>JTC_762625_1

TCCTCACCGTGCTTTCCTTGATTTTGGCATAAT

>JTC_3390115_1

TCGGGATGATATCGGCCAAGAACGTTTTCACGA

>JTC_3216320_1

TGTAAGCAAGCCCTAGAAGATCTTGAACGTGAG

>JTC_3017435_2

ATATGAAGTCGCAGTACCGTTGTGTAAGCAAGC

>JTB_4064669_1

GGAAGCGCTACGTACTGAGCTGCAGTCTATCCG

>JTB_4664620_1

CGGTGAGGTGGAACTGTACATTCGAAGAGCGTT

>JTB_3440973_1

TGATGATCCAGCATTTGACCTATTCCCCGAAGA

>JTC_3366403_1

TCTTGATCGATGCTGCGAATTCCATCTGTCTCT

>JTC_4222695_1

GAAAGACAGTCAGCAGCAACTGCTGGAAGCTCA

>JTC_292731_2

CTGGGACGGTGTTGGTGTCGACTTGCTAAGTCG

>JTC_2508384_1

AGTTGAACAACCTCGCATTATTATGCCAAAATC

>JTB_2496974_1

ATTCTGATCTCGGTAGACCATGGTCAGGATGCT

>JTC_4454586_1

CTAAGTCGTCTTTCTGCAATTTCATCTTCGGGG

>JTB_4842658_1

CGATTATGAAGTGTCTCCACGTCTAAAGACTAT

>JTC_3804395_1

GGCTTTGCTTGACTGTCTTCATTTTGGAGAGCA

>JTC_4491559_1

CGTCAAAGTTGCCCAATCTTTGATGCAACGAGG

>JTB_5071484_1

CATCGATCAAGAAATACGACGACTTTTTGGAAG

>JTB_2594475_1

AGATTCCGCTTCCTTGTAGTTACCGTTCTTTCC

>JTC_5013652_1

CAACAGCTGAGCTTCTCCCAATCCTAGTTCGAT

>JTB_2986568_2

GTCGCAGTACCGTTGTGTAAGCAAGCCCTAGAA

>JTB_1209496_1

GGCGGGCTTGCTTACACAACGGTACTGCGACTT

>JTC_835255_1

GTTTTGTATGGAAAGAACGGTAACTACAAGGCA

>JTC_348527_2

CAGCAGTGGTTCACTCTCCAACGGTCAAAAGTA

>JTC_3629627_1

GTGAGAATGGCCGTGAACATCCAGATGTGGCAA

>JTC_3045833_1

TTTTGACCAACACGTCTCTTAACAACTGGTCAG

>JTC_675540_1

TGATCTCGGTAGACCATGGTCAGGATGCTTACC

>JTC_1144565_1

GGGACGGTGTTGGTGTCGACTTGCTAAGTCGTC

>JTB_3547238_1

TCATCCCGTAGCCAGGAGGTTTCCTGACAAAGT

>JTC_4444600_1

CTAGTTCGATGTTCTGCGCATTTCGATTGATTA

>JTB_2730987_1

AAAGAAGCTGACCAGTTGTTAAGAGACGTGTTG

>JTB_304042_2

CTTCCTTGTAGTTACCGTTCTTTCCATACAAAA

>JTC_195952_2

TGGCCGTTTTGTATGGAAAGAACGGTAACTACA

>JTB_2968037_2

GTTTTGTATGGAAAGAACGGTAACTACAAGGAA

>JTB_4105605_1

GCTTTCCTTGATTTTGGCATAATAATGCGAGGT

>JTC_190366_2

TGTTGGTGTCGACTTGCTAAGTCGTCTTTCTGC

>JTC_4484973_1

CGTCGATGCTTTTCAGGTGAGATAATAAGGCGC

>JTC_1517977_1

GACTTCATATCTCCCCTTTGAGGCGTAGCTTAA

>JTB_3313448_1

TGTGATTCGAAAATTTCCAACGCTCTTCGAATG

>JTC_4943647_1

CAGATGTGGCAACAATGTTAAGCATCCTGACCA

>JTB_5206308_1

ATTTTGGAGAGCAGACTCTCGTCGGTCATCAGC

>JTB_765851_1

TCTCGGTAGACCATGGTCAGGATGCTTAACATT

>JTC_2491533_1

ATCCAAAATCTCCTGTTTGCTCCGGAACTGACC

>JTB_3256666_1

TTCGATGAGACACAGAATGGTGACAGTGAATCA

>JTC_5170936_1

AGATAATAAGGCGACCAACAGCTGAGCTTCTCC

>JTB_4629916_1

CGTGCTCCACTTTTACCGTAAGAAGTGTTATCG

>JTC_2649643_1

AAGCAAGCCCTAGAAGATCTTGAACGTGAGACT

>JTB_4361327_1

GAACTTCTCGTCGAATTCTACACCTTCCAAAAA

>JTC_5313864_1

AAAAGACAGTCAGCAGCAACTGCTGGAAGCTCA

>JTC_3062703_1

TTTGGCTAATCGTGAAAACGTTCTTGGCCGATA

>JTB_1675265_1

CTTGGCATTAAGCTACGCCTCAAAGGGGAGATA

>JTB_1745322_1

CTGGGACGGTGTTGGTGTCGACTTGCTAAGTCG

>JTB_559451_1

TGTTGGTGTCGACTTGCTAAGTCGTCTTTCTCC

>JTB_2338641_1

CATCCATCGGTGGCAGCGGCTTTAAATAATTTT

>JTB_2169884_1

CGAACTTCTCGTCGAATTCTACACCTTCCAAAA

>JTC_1535119_1

GAATTCCTTATCCTGCGTTTTGACCAACACGTC

>JTB_2214258_1

CCGTGGGTGAGTGTCATCCATCGGTGGCAGCGG

>JTC_4056022_1

GCCGTTTTGTATGGAAAGAACGGTAACTACAAG

>JTC_208395_2

TCGGGATGATATCGGCCAAGAACGTTTTCACGA

>JTB_3820611_1

GTCAGGATGCTTAACATTGTTGCCACATCTGGA

>JTB_453399_1

TTTCGATTGATGACTTCTTCCTTCTCCGGATTG

>JTC_3348886_1

TGAAGTCGCAGTACCGTTGTGTAAGCAAGCCCT

>JTB_4439265_1

CTTGCATAAAGATTCCGCTTCCTTGTAGTTACC

>JTC_4510776_1

CGGTGATGATCCAGCATTTGACCTATTCCCCGA

>JTB_566306_1

TGTTATCGACGTTAATTGATTCACTGTCACCCT

>JTC_843569_1

GTTTTCACGATTAGCCAAAGCTCTCTTGCATAG

>JTC_134235_3

GGAGAGTGAACCACTGCTGTTTCGTGCCATGCT

>JTB_1878195_1

CGTGCTTTCCTTGATTTTGGCATAATAATGCGC

>JTC_2410301_1

CAAAGCCTGGAAGCGCTACGTACTGAGCTGCCG

>JTC_5081982_1

ATTACACTTGGTTTTGATGGCATTGGGATCGAT

>JTB_523410_1

TTCGAAGAGCGTTGGAAATTTTCGAATCACAGC

>JTB_2644365_1

ACGGTGTTGGTGTCGACTTGCTAAGTCGTCTTT

>JTC_5098169_1

ATCGTTAGCACCTAGCTGTGATTCGAAAATTTC

>JTB_1906727_1

CGGTGTTGGTGTCGACTTGCTAAGTCGTCTTTC

>JTB_3589392_1

TAAGCAAGCCCTAGAAGATCTTGAACGTGAGAA

>JTB_350464_2

CGAAACAGCAGTGGTTCACTCTCCAACGGTCAA

>JTC_2649644_1

AAGCAAGCCCTAGAAGATCTTGAACGTGAGAAT

>JTC_2258229_1

CCAGTACAGATCCCATCTCCAATCCGGAGAAGG

>JTB_242822_2

GTCGACTTGCTAAGTCGTCTTTCTGCAATTTCA

>JTB_1365662_1

GCTGTTTGAAACTCCGTAAGTACAAAGAAGCTG

>JTC_2699805_1

AAAAGACAGTCAGCAGCAACTGCTGGAAGCTCA

>JTC_2254623_1

CCATGCTCCACTTTTACCGTAAGAAGTGTTATC

>JTC_940087_1

GTTCACTCTCCAACGGTCAAAAGTATACTAAAG

>JTC_4922950_1

CAGTACCGTTGTGTAAGCAAGCCCTAGAAGATC

>JTC_4733471_1

CGACCAACAGCTGAGCTTCTCCCAATCCTAGTT

>JTB_3287702_1

TTACACTTGGTTTTGATGGCATTGGGATCGTTA

>JTC_703887_1

TGAAGTCGCAGTACCGTTGTGTAAGCAAGCCCT

>JTC_5243654_1

AATTCGATGAGACACAGAATGGTGACAGTGAAT

>JTB_4678310_1

CGGGTGAGTGTCATCCATCGGTGGCAGCGGCCC

>JTC_2650939_1

AAGATCTTGAACGTGAGAATGGCCGTGAACCTC

>JTC_880927_1

GTTGTTTCGTGCCATGCTCCACTTTTACCGTAA

>JTC_5073727_1

ATTCGACGAGAAGTTCGGTGATGATCCAGCATT

>JTB_5284367_1

ATAATCGGGATAATGCTGGGACGGTGTTGGTGT

>JTC_804598_1

TAGAATTCGACGAGAAGTTCGGTGATGATCCAG

>JTC_3478032_1

GTTTTGTATGGAAAGAACGGTAACTACAAGCAA

>JTB_2359649_1

CAGCTTCTTTGTACTTACGGAGTTTCAAACAGC

>JTC_3486877_1

GTTTTCACGATTAGCCAAAGCTCTCTTGCATAA

>JTC_3685198_1

GTCAGCTTCTTTGTACTTACGGAGTTTCAAACA

>JTC_3645629_1

GTCTCATCGAATTCCTTATCCTGCGTTTTGACC

>JTC_177341_3

AAAAAGTCGTCGTATTTCTTGATCGATGCTGTG

>JTC_286884_2

CTTGAACGTGAGAATGGCCGTGAACATCCAGAT

>JTB_750529_1

TGAAAACGTTCTTGGCCGATATCATCCCGATGT

>JTB_958141_1

GTTCGGTGATGATCCAGCATTTGACCTATTCCC

>JTC_3666227_1

GTCGACTTTTTGGAAGGTGTAGAATTCGACGAG

>JTC_741129_1

TCGTCTTTCTGCAATTTCATCTTCGGGGAATAG

>JTB_1857407_1

CTAAGTCGTCTTTCTGCAATTTCATCTTCGGGG

>JTB_5418186_1

AATTCGATGAGACACAGAATGGTGACAGTGAAT

>JTC_820298_1

TAACATTGTTGCCACATCTGGATGTTCACGGCC

>JTC_5090397_1

ATGATATCGGCCAAGAACGTTTTCACGATTAGC

>JTB_2937327_2

TTGAATAGTCTTTAGACGTGGAGACACTTCATA

>JTB_2978116_2

GTTCGATGTTCTGCGCATTTCGATTGATGACTT

>JTB_2191646_1

CCTTATCCTGCGTTTTGACCAACACGTCTCTTA

>JTB_2337267_1

CATCGATCAAGAAATACGACGACTTTTTGGAAG

>JTB_2427001_1

CAAGCCCTAGAAGATCTTGAACGTGAGAATGGC

>JTC_944776_1

GTTATTCTGATCTCGGTAGACCATGGTCAGGAT

>JTB_2715864_1

AACAAGTGTAATCTAGCTGCATGCTGTTTGAAA

>JTB_941333_1

GTTGATAACACTTCTTACGGTAAAAGTGGAGCA

>JTC_4446481_1

CTAGATTACACTTGGTTTTGATGGCATTGGGAT

>JTB_757876_1

TCTGTGTCTCATCGAATTCCTTATCCTGCGTTT

>JTC_2950880_2

CTTTAGTATACTTTTGACCGTTGGAGAGTGAAC

>JTC_2833187_3

CTTGAACGTGAGAATGGCCGTGAACATCCAGAT

>JTC_1149534_1

GGGAATAGGTCAAATGCTGGATCATCACCGAAC

>JTB_2433436_1

CAACTGGTCAGCTTCTTTGTACTTACGGAGTTT

>JTB_3086070_2

CGAAGATGAAATTGCAGAAAGACGACTTAGCAA

>JTC_2814463_3

GTTGTGTAAGCAAGCCCTAGAAGATCTTGAACG

>JTC_1184403_1

GGCCAAGAACGTTTTCACGATTAGCCAAAGCTC

>JTB_5087324_1

CAGGCTTTGCTTGACTGTCTTCATTTTGGAGAG

>JTC_4290633_1

CTTCTTTGTACTTACGGAGTTTCAAACAGCATG

>JTB_2501296_1

ATTCGAAGAGCGTTGGAAATTTTCGAATCACAG

>JTB_2970892_2

GTTTGATGGCTTCTGGTAGGTTATTCTGATCTC

>JTC_3744266_1

GGTCAGCAGCAACTGCTGGAAGCTCAACTTAAA

>JTB_5095325_1

CAGCTAGATTACACTTGGTTTTGATGGCATTGG

>JTC_2941017_2

GATCTCGGTAGACCATGGTCAGGATGCTTAACA

>JTC_3271292_1

TGGAAGGTGTAGAATTCGACGAGAAGTTCGGTG

>JTB_512687_1

TTCGTGCCATGCTCCACTTTTACCGTAAGAAGT

>JTB_5326568_1

AGCATTATCCCGATTATGAAGTGTCTCCACGGC

>JTB_4266280_1

GCAAGCCCTAGAAGATCTTGAACGTGAGAATGG

>JTC_307659_2

CGGTGTTGGTGTCGACTTGCTAAGTCGTCTTTC

>JTB_980217_1

GTTCAACGTCGATGCTTTTCAGGTGAGATAATA

>JTB_2365855_1

CAGCATTTGACCTATTCCCCGAAGATGAAATTG

>JTC_3635908_1

GTGAACCACTGCTGTTTCGTGCCATGCTCCACT

>JTC_4387452_1

CTCTTGCATAAAGATTCCGCTTCCTTGTAGTTA

>JTB_1304155_1

GGAATCTTTATGCAAGAGAGCTTTGGCTAATCG

>JTB_4923305_1

CGAAACAGCAGTGGTTCACTCTCCAACGGTCAA

>JTC_3714438_1

GTAAAAGACAGTCAGCAGCAACTGCTGGAAGCT

>JTC_675541_1

TGATCTCGGTAGACCATGGTCAGGATGCTTAAC

>JTC_2922891_2

GGAATTCGATGAGACACAGAATGGTGACAGTGA

>JTB_829102_1

TATCGATCCCAATGCCATCAAAACCAAGTGTAA

>JTC_4166582_1

GAGAAGCTCAGCTGTTGGTCGCCTTATTATCTC

>JTC_3505880_1

GTTTGAAACTCCGTAAGTACAAAGAAGCTGACC

>JTC_537625_1

TGTTCAACTGTTTTGCCACATCGGGATGATATC

>JTB_420732_1

TTTTCGAATCACAGCTAGGTGCTAACGATCCCA

>JTC_4140595_1

GATGAAGTAAAAGACAGTCAGCAGCAACTGCTG

>JTB_3639905_1

GTTTCGTGCCATGCTCCACTTTTACCGTAAGAA

>JTC_495034_1

TTCGACGAGAAGTTCGGTGATGATCCAGCATTT

>JTC_1462305_1

GCAAGCCCTAGAAGATCTTGAACGTGAGAATGG

>JTC_463772_1

TTGCTAAGTCGTCTTTCTGCAATTTCATCTTCG

>JTC_1052422_1

GTAGAATTCGACGAGAAGTTCGGTGATGATCCA

>JTB_4441203_1

CTTGATCGATGCTGCGAATTCCATCTGTCTCTT

>JTC_217382_2

GTTTGGGTGAGTGTCATCCATCGGTGGCAGCGG

>JTC_2952566_2

CTTGATTTTGGCATAATAATGCGAGGTTGTTCA

>JTC_2346958_1

CACGGGATGAAGTAAAAGACAGTCAGCAGCAAC

>JTC_984809_1

GTGAATTCCATCTGTCTCTTTTCCTCTTCGAGC

>JTC_1017973_1

GTCGACTTGCTAAGTCGTCTTTCTGCAATTTCC

>JTC_3905603_1

GGAATTCGCAGCATCGATCAAGAAATACGACGA

>JTC_3529609_1

GTTGTTAAGAGACGTGTTGGTCAAAACGCAGGA

>JTC_5263376_1

AAGCAAGCCCTAGAAGATCTTGAACGTGAGAAT

>JTC_1566205_1

GAAATACGACGACTTTTTGGAAGGTGTAGAATT

>JTC_606226_1

TGGCAAAACAGTTGAACAACCTCGCATTATTAT

>JTC_2175272_1

CCGTATCGGATAGACTGCAGCTCAGTACGTAGC

>JTB_4250547_1

GCAGCATCGATCAAGAAATACGACGACTTTTTG

>JTC_3529259_1

GTTGTTCAACTGTTTTGCCACATCGGGATGATA

>JTC_3380539_1

TCTCCAACGGTCAAAAGTATACTAAAGCATATG

>JTC_5175097_1

AGAGAGCTTTGGCTAATCGTGAAAACGTTCTTG

>JTB_4884812_1

CGACGAGAAGTTCGGTGATGATCCAGCATTTGA

>JTC_4249889_1

CTTTTACCGTAAGAAGTGTTATCGACGTTAATT

>JTC_4099296_1

GCAGCATCGATCAAGAAATACGACGACTTTTTG

>JTB_3548707_1

TCAGGATGCTTAACATTGTTGCCACATCTGGAT

>JTC_5146125_1

AGGAATTCGATGAGACACAGAATGGTGACAGTG

>JTB_1652465_1

CTTTGCTTGACTGTCTTCATTTTGGAGAGCAGA

>JTB_1438806_1

GCGACCAACAGCTGAGCTTCTCCCAATCCTAGT

>JTB_5117378_1

CAGAAAGACGACTTAGCAAGTCGACACCAACAC

>JTC_5284596_1

AAATTTTCGAATCACAGCTAGGTGCTAACGATC

>JTB_4406345_1

CTTTTACCGTAAGAAGTGTTATCGACGTTAATT

>JTB_341484_2

CGATTATGAAGTGTCTCCACGTCTAAAGACTAT

>JTC_4965778_1

CACGATTAGCCAAAGCTCTCTTGCATAAAGATT

>JTB_706408_1

TGATGATCCAGCATTTGACCTATTCCCCGAAGA

>JTC_2517599_1

AGGTTATTCTGATCTCGGTAGACCATGGTCAGG

>JTC_4421067_1

CTCCGGAACTGACCGTGTTTCAGCTTGGATGAC

>JTC_2514086_1

AGTCAGCAGCAACTGCTGGAAGCTCAACTTAAA

>JTC_2379357_1

CAAGGCTTCATTCATATGTTTGATGGCTTCTGG

>JTB_212767_2

TGAACATCCAGATGTGGCAACAATGTTAAGCAT

>JTC_2510224_1

AGTGTCTCCACGTCTAAAGACTATTCAAAACTT

>JTC_997231_1

GTCTCATCGAATTCCTTATCCTGCGTTTTGACC

>JTB_2571958_1

AGGATAAGGAATTCGATGAGACACAGAATGGTG

>JTC_5260352_1

AAGGAAGCGGAATCTTTATGCAAGAGAGCTTTT

>JTC_4857197_1

CCCGTTGGAGAGTGAACCACTGCTGTTTCGTGC

>JTC_4058207_1

GCCGTGAACATCCAGATGTGGCAACAATGTTAA

>JTC_556312_1

TGTCGACTTGCTAAGTCGTCTTTCTGCAATTTC

>JTC_3192046_1

TGTGTAAGCAAGCCCTAGAAGATCTTGAACGTG

>JTB_4501375_1

CTGGGACGGTGTTGGTGTCGACTTGCTAAGTCG

>JTC_2088259_1

CGACTTGCTAAGTCGTCTTTCTGCAATTTCATC

>JTB_4333613_1

GACCGTTGGAGAGTGAACCACTGCTGTTTCGTG

>JTC_1800436_1

CTAGATTACACTTGGTTTTGATGGCATTGGGAT

>JTB_2624251_1

AGAACGTTTTCACGATTAGCCAAAGCTCTCTTT

**2) Illumina reads that matched AAEL014967**

>JTB_904055_1

GTTTCCTGTACCACACTCAACGTTGCTGATGAC

>JTC_1270893_1

GGAAGCGCTACGTACTGAGCTGCAGTCTATCCG

>JTC_673531_1

TGATGATCCAGCATTTGACCTATTCCCCGAAGA

>JTC_2620716_1

ACACAACGGTACTGCGACTTCATATCTTCCCTT

>JTC_1066718_1

GGTTTTGATGGCATTGGGATCGATAACACCTAG

>JTB_908993_1

GTTTCAAACAGCATGCAGCTAGATTACACTTGG

>JTC_2805422_3

TTCGAAGAGCGTTGGAAATTTTTGAATCGCAGC

>JTC_3357275_1

TGAACATCCAGATGTGGCAACAATGTTAAGCCT

>JTB_2026589_1

CGGAAGGGAAGATATGAAGTCGCAGTACCGTTG

>JTC_565508_1

TGTACATTCGAAGAGCGTTGGAAATTTTTGAAT

>JTC_3419758_1

TCATCACCGAACTTCTCGTCGAATTCTACACCT

>JTB_1774651_1

CTGATCTCGGTAGACCATGGTCAGGATGCTTAA

>JTB_198396_2

TGTCGACTTGCTAAGTCGTCTTTCTGCAATTTC

>JTB_4445128_1

CTTGAACGTGAGAATGGCCGTGAACATCCAGAT

>JTC_420067_1

TTTGAATCGCAGCTAGGTGTTATCGATCCCCAT

>JTC_359079_2

ATTTTTGAATCGCAGCTAGGTGTTATCGATCCC

>JTB_4294730_1

GATCTCGGTAGACCATGGTCAGGATGCTTAACA

>JTC_3446745_1

TAGAATTCGACGAGAAGTTCGGTGATGATCCAG

>JTC_3114570_1

TTGCTCCGGAACTGACCGTGTTTCAGCTTGGAT

>JTC_2895130_2

GTTGTGTAAGCAAGCCCTGGAAGATCTTGAACG

>JTC_2596346_1

ACTATTCAAAACTTGGCATTAAACTACGCCTCG

>JTB_3830458_1

GTATTTCTTGATCGATGCTGTGAATTCCATCTG

>JTC_2779749_4

TGAACATCCAGATGTGGCAACAATGTTAAGCAT

>JTC_5095071_1

ATCTTCCAGGGCTTGCTTACACAACGGTACTGC

>JTC_745619_1

TCGGGGAATAGGTCAAATGCTGGATCATCACCG

>JTC_3009268_2

CAACTGTTTTGCCACATCGGGATGATATCGGCC

>JTB_3296453_1

TGTTGGTCAAAACGCAGGATAAGGAATTCGATG

>JTC_4402311_1

CTCGGTAGACCATGGTCAGGATGCTTAACATTG

>JTB_2989760_2

GTCAAAAGTATACTAAAGCATATGGCCTCGTTG

>JTB_4482891_1

CTTACACAACGGTACTGCGACTTCATATCTTCC

>JTC_3348887_1

TGAAGTCGCAGTACCGTTGTGTAAGCAAGCCCC

>JTC_421171_1

TTTGAAACTCCGTAAGTACAAAGAAGCTGACCA

>JTC_2294915_1

CATCAGCAACGTTGAGTGTGGTACAGGAAACAT

>JTB_4351022_1

GAAGTCGCAGTACCGTTGTGTAAGCAAGCCCTA

>JTC_3286554_1

TGCGTTTTGACCAACACGTCTCTTAACAACTGG

>JTC_1721042_1

CTGCAATTTCATCTTCGGGGAATAGGTCAAATG

>JTC_4328813_1

CTTACACAACGGTACTGCGACTTCATATCTTCC

>JTB_3527957_1

TCGAAGGGAAGATATGAAGTCGCAGTACCGTTG

>JTC_1738676_1

CTCTTGCATAAAGATTCCGCTTCCTTGTAGTTA

>JTC_646039_1

TGCGCTTCCAGGCTTTGCTTCACTGTCTTCATT

>JTC_2497531_1

ATATGGCCTCGTTGCACCAAAGATTGGGCAACT

>JTC_5006733_1

CAACGGTCAAAAGTATACTAAAGCATATGGCCT

>JTC_3984250_1

GCTGCATGCTGTTTGAAACTCCGTAAGTACAAA

>JTC_214248_2

TAAAGATTCCGCTTCCTTGTAGTTACCGTTCTT

>JTC_2647817_1

AAGCGGAATCTTTATGCAAGAGAGCTTTGGCTA

>JTC_5200518_1

AGAAAGACGACTTAGCAAGTCGACACCAACACC

>JTC_3973921_1

GCTGTTTCGTGCCATGCTCCACTTTTACCGTCA

>JTC_1405977_1

GCCGTGAACATCCAGATGTGGCAACAATGTTAA

>JTC_3744052_1

GGTCAGCTTCTTTGTACTTACGGAGTTTCAAAC

>JTB_5296596_1

AGTCAGCAGCAACTGCTGGAAGCTCAACTTACA

>JTC_4516433_1

CGGTATCATCCCGATGTGGCAAAACAGTTGAAC

>JTC_3248682_1

TGGCCGTTTTGTATGGAAAGAACGGTAACTACA

>JTC_2959504_2

CTGCGATTCAAAAATTTCCAACGCTCTTCGAAT

>JTB_768557_1

TCTCCCAATCCTAGTTCGATGTTCTGCGCATTT

>JTC_2419556_1

CAAAAGTATACTAAAGCATATGGCCTCGTTGCA

>JTB_3086174_2

CGAAGAGCGTTGGAAATTTTTGAATCGCAGCTA

>JTC_5265013_1

AAGATATGAAGTCGCAGTACCGTTGTGTAAGCA

>JTC_3164840_1

TTATTCTGATCTCGGTAGACCATGGTCAGGATG

>JTB_2462050_1

CAAAAGTATACTAAAGCATATGGCCTCGTTGCA

>JTC_4783413_1

CCTGCGCATTTCGATTGATGACTTCTTCCTTCT

>JTB_507686_1

TTCTGATCTCGGTAGACCATGGTCAGGATGCTT

>JTB_2656703_1

ACCTATTCCCCGAAGATGAAATTGCAGAAAGAC

>JTB_4410663_1

CTTTGCTTCACTGTCTTCATTTTGGAGAGCAGA

>JTC_3385581_1

TCGTGAAAACGTTCTTGGCCGATATCANCCCGA

>JTC_4912845_1

CATCCACAATATCCAAAATCTCCTGTTTGCTCC

>JTB_3220397_1

TTGGATGACACGTCAAAGTTGCCCAATCTTTGG

>JTC_3403375_1

TCCTTATCCTGCGTTTTGACCAACACGTCTCTT

>JTC_1256146_1

GGAATTCGATGAGGTACAGAATGGTGACAGTGA

>JTC_4718180_1

CGACTTGCTAAGTCGTCTTTCTGCAATTTCATC

>JTC_2660231_1

AACTGCTGGAAGCTCAACTTAAAATAGTCCAGC

>JTC_113711_3

TTCTGATCTCGGTAGACCATGGTCAGGATGCTT

>JTC_2947359_2

GAAACAGCAGTGGTTCACTCTCCAACGGTCAAA

>JTC_3067068_1

TTTGCCACATCGGGATGATATCGGCCAAGAACG

>JTB_821036_1

TCAAAACCAAGTGTAATCTAGCTGCATGCTGTT

>JTB_3189551_1

TTTCGATTGATGACTTCTTCCTTCTCCGGATTG

>JTC_1814104_1

CGTTTTCACGATTAGCCAAAGCTCTCTTGCATA

>JTC_2466266_1

ATTACACTTGGTTTTGATGGCATTGGGATCGAT

>JTB_2391798_1

CACGGTCAGTTCCGGAGCAAACAGGAGATTTTG

>JTC_4766634_1

CGAAACAGCAGTGGTTCACTCTCCAACGGTCAC

>JTB_779348_1

TCGGGATGATATCGGCCAAGAACGTTTTCACGC

>JTC_3949200_1

GGAAAGAACGGTAACTACAAGGAAGCGGAATCT

>JTC_1323353_1

GCTGTACATTCGAAGAGCGTTGGAAATTTTTGA

>JTB_4206882_1

GCCGTGAACATCCAGATGTGGCAACAATGTTAA

>JTB_4593344_1

CTCAACTTAAAATAGTCCAGCTCGAAGAGGAAA

>JTC_789517_1

TATGGGTTCGCTGTTTGGGTGAGTGTCATCCAT

>JTC_4990314_1

CAATATCCAAAATCTCCTGTTTGCTCCGGAACT

>JTB_4446225_1

CTTCTTTGTACTTACGGAGTTTCAAACAGCATG

>JTC_3105174_1

TTGGTGTCGACTTGCTAAGTCGTCTTTCTGCAA

>JTC_1867576_1

CGGTGATGATCCAGCATTTGACCTATTCCCCGC

>JTC_1198194_1

GGATTGGGAGAAGCTCAGCTGTTGGTCGCCTTA

>JTC_3906086_1

GGAATTCACAGCATCGATCAAGAAATACGACGA

>JTC_1388917_1

GCGACTTCATATCTTCCCTTCGAGGCGTAGTTT

>JTC_380075_2

AAACAGCAGTGGTTCACTCTCCAACGGTCAAAA

>JTC_3846156_1

GGATTGGGAGAAGCTCAGCTGTTGGTCGCCTTA

>JTB_4320889_1

GAGAAGCTCAGCTGTTGGTCGCCTTATTATCTT

>JTC_3587639_1

GTTCACTCTCCAACGGTCAAAAGTATACTAAAG

>JTC_865491_1

GTTTCGTGCCATGCTCCACTTTTACCGTAAGAA

>JTC_4686137_1

CGATTATGAAGTGTCTCCACGTCTAAAGACTAT

>JTC_4938129_1

CAGCATGCAGCTAGATTACACTTGGTTTTGATG

>JTC_2386500_1

CAAGAACGTTTTCACGATTAGCCAAAGCTCTCT

>JTB_621468_1

TGGGATCGATAACACCTAGCTGCGATTCAAAAA

>JTC_4696810_1

CGATCCCAATGCCATCAAAACCAAGTGTAATCT

>JTC_1256573_1

GGAATTCACAGCATCGATCAAGAAATACGACGA

>JTB_3114200_2

ATTTGGCCGTTTTGTATGGAAAGAACGGTAACT

>JTC_2374811_1

CAATATCCAAAATCTCCTGTTTGCTCCGGAACT

>JTC_456635_1

TTGGGATCGATAACACCTAGCTGCGATTCAAAA

>JTB_5061714_1

CATTGTTGCCACATCTGGATGTTCACGGCCCTT

>JTB_441796_1

TTTGACTTGTATGCGAAGCGTTTGCCGTTCCCC

>JTB_1838453_1

CTCACCTGAAAAGCATCGATGTTGAACGGCAAG

>JTC_2074658_1

CGAGGTTGTTCAACTGTTTTGCCACATCGGGAT

>JTC_4633847_1

CGCTCTTCGAATGTACAGCTCCACCTCACCGTG

>JTC_1798526_1

CTAGTTCGATGTTCTGCGCATTTCGATTGATGA

>JTC_4517525_1

CGGTAGACCATGGTCAGGATGCTTAACATTGTT

>JTB_5377578_1

ACTTCATCCCGTAGCCAGGAGGTTTCCTGACAC

>JTC_5240734_1

AATTTGGCCGTTTTGTATGGAAAGAACGGTAAC

>JTC_2653832_1

AAGACAGTCAGCAGCAACTGCTGGAAGCTCAAC

>JTB_2974463_2

GTTGGTCAAAACGCAGGATAAGGAATTCGATGA

>JTC_2690379_1

AAACAGCAGTGGTTCACTCTCCAACGGTCAAAG

>JTC_1234073_1

GGAGACACTTCATAATCGGGATAGTGCTGGGTC

>JTC_2292530_1

CATCGAACTAGGATTGGGAGAAGCTCAGCTGAG

>JTC_1206494_1

GGATGATATCGGCCAAGAACGTTTTCACGATTA

>JTC_4730798_1

CGACCGTTGGAGAGTGAACCACTGCTGTTTCGT

>JTB_2607972_1

AGACTCTCGTCGGTCATCAGCAACGTTGAGTGT

>JTB_2519963_1

ATGACACTCACCCAAACAGCGAACCCATATGCC

>JTB_948490_1

GTTCTTGGCCGATATCATCCCGATGTGGCAAAA

>JTB_2397467_1

CACGAAACAGCAGTGGTTCACTCTCCAACGGTC

>JTC_3921159_1

GGAAGCGGAATCTTTATGCAAGAGAGCTTTGGC

>JTC_5132377_1

AGGTTATTCTGATCTCGGTAGACCATGGTCAGG

>JTC_2154988_1

CCTGCGTTTTGACCAACACGTCTCTTAACAACT

>JTB_3883388_1

GGTCAAAACGCAGGATAAGGAATTCGATGAGGT

>JTB_1300652_1

GGAATTCACAGCATCGATCAAGAAATACGAAGA

>JTB_1095074_1

GTACGTAGCGCTTCCAGGCTTTGCTTCACTGTC

>JTB_1771093_1

CTGCAATTTCATCTTCGGGGAATAGGTCAAATG

>JTC_2054851_1

CGATTATGAAGTGTCTCCACGTCTAAAGACTAT

>JTC_2609985_1

ACGAAACAGCAGTGGTTCACTCTCCAACGGTCA

>JTC_5167166_1

AGATCTTCCAGGGCTTGCTTACACAACGGTACT

>JTB_3481050_1

TGAACATCCAGATGTGGCAACAATGTTAAGCAT

>JTB_5364396_1

AGAACGTTTTCACGATTAGCCAAAGCTCTCTTG

>JTB_302003_2

CTTCTTTGTACTTACGGAGTTTCAAACAGCATG

>JTC_3783424_1

GGGATGCTTAACATTGTTGCCACATCTGGATGT

>JTC_1621894_1

CTTGGGTATATGGGTTCGCTGTTTGGGTGAGTG

>JTC_87965_4

GTAAAAGACAGTCAGCAGCAACTGCTGGAAGCT

>JTC_338550_2

CCGGAACTGACCGTGTTTCAGCTTGGATGACAC

>JTC_3462174_1

TAACATTGTTGCCACATCTGGATGTTCACGGCC

>JTB_730881_1

TGACAGTGAATCAATTAACGTTGATAACACTTC

>JTB_2263805_1

CCGATATCATCCCGATGTGGCAAAACAGTTGAA

>JTC_1436406_1

GCCAAAATCAAGGAAAGCACGGTGAGGTGGAGC

>JTB_858589_1

TAAAGATTCCGCTTCCTTGTAGTTACCGTTCTT

>JTB_3237057_1

TTGAACAACCTCGCATTATTATGCCAAAATCAA

>JTC_410614_1

TTTGGGTGAGTGTCATCCATCGGTGGCAGCGGC

>JTB_3023585_2

GCCATATGCTTTAGTATACTTTTGACCGTTGGA

>JTC_1217088_1

GGAGTTTCAAACAGCATGCAGCTAGATTACACT

>JTC_3832074_1

GGCCAAGAACGTTTTCACGATTAGCCAAAGCTC

>JTC_2920910_2

GGAGAGTGAACCACTGCTGTTTCGTGCCATGCT

>JTB_2343906_1

CATAAAGATTCCGCTTCCTTGTAGTTACCGTTC

>JTC_896837_1

GTTGGAAATTTTTGAATCGCAGCTAGGTGTTAT

>JTB_4032423_1

GGACGGTGTTGGTGTCGACTTGCTAAGTCGTCT

>JTB_5195843_1

CAAAAGTATACTAAAGCATATGGCCTCGTTGCA

>JTB_4067262_1

GGAAGATCTTGAACGTGAGAATGGCCGTGAACA

>JTC_2421364_1

CAAAACGCAGGATAAGGAATTCGATGAGGTACA

>JTB_4570958_1

CTCCTGTTTGCTCCGGAACTGACCGTGTTTCAG

>JTC_2513047_1

AGTCGCAGTACCGTTGTGTAAGCAAGCCCTGGA

>JTC_2812727_3

GTTTTGTATGGAAAGAACGGTAACTACAAGGAA

>JTC_5267518_1

AAGACAGTCAGCAGCAACTGCTGGAAGCTCAAC

>JTC_4985739_1

CAATTAACGTTGATAACACTTCTTACGGTAAAA

>JTC_3026323_2

AATTTTTGAATCGCAGCTAGGTGTTATCGATCC

>JTB_2602269_1

AGATAATAAGGCGACCAACAGCTGAGCTTCTCC

>JTB_828449_1

TATCGGCCAAGAACGTTTTCACGATTAGCCAAA

>JTC_1055274_1

GTACCGTTGTGTAAGCAAGCCCTGGAAGATCTT

>JTB_2721373_1

AAATGCTGGATCATCACCGAACTTCTCGTCGAA

>JTC_4537362_1

CGGGGAATAGGTCAAATGCTGGATCATCACCGA

>JTC_4194229_1

GAAGTTCGGTGATGATCCAGCATTTGACCTATT

>JTC_1630623_1

CTTGCATAAAGATTCCGCTTCCTTGTAGTTACC

>JTC_4114623_1

GCAAGCCCTGGAAGATCTTGAACGTGAGAATGG

>JTC_579137_1

TGGTCAGGATGCTTAACATTGTTGCCACATCTG

>JTC_2871193_2

TGTCGACTTGCTAAGTCGTCTTTCTGCAATTTC

>JTC_393985_1

TTTTGATGGCATTGGGATCGATAACACCTAGCT

>JTC_759973_1

TCCTTATCCTGCGTTTTGACCAACACGTCTCTT

>JTB_3855647_1

GGTTGTTCAACTGTTTTGCCACATCGGGATGAT

>JTB_4789486_1

CGCTGCCACCGATGGATGACACTCACCCAAACA

>JTC_3719384_1

GGTTGTTCAACTGTTTTGCCACATCGGGATGCT

>JTC_1754194_1

CTCGGTAGACCATGGTCAGGATGCTTAACATTG

>JTC_2777305_4

TTCTGATCTCGGTAGACCATGGTCAGGATGCTT

>JTC_3360702_1

TGAAAGACAGTCAGCAGCAACTGCTGGAAGCTC

>JTB_2959913_2

TCTCGGTAGACCATGGTCAGGATGCTTAACATT

>JTC_1213782_1

GGATAGTGCTGGGACGGTGTTGGTGTCGACTTG

>JTB_3472238_1

TGAAGTCGCAGTACCGTTGTGTAAGCAAGCCCT

>JTC_4840246_1

CCGATGGATGACACTCACCCAAACAGCGAACCC

>JTB_2873497_3

GTTTTGACCAACACGTCTCTTAACAACTGGTCA

>JTC_1953624_1

CGGAGTTTCAAACAGCATGCAGCTAGATTACAC

>JTC_3413198_1

TCCCGATGTGGCAAAACAGTTGAACAACCTCGC

>JTB_1706813_1

CTTCCTTCTCCGGATTGGAGATGGGATCTGCAC

>JTB_1622169_1

GAAAGACAGTCAGCAGCAACTGCTGGAAGCTCC

>JTC_1086185_1

GGTGAGTGTCATCCATCGGTGGCAGCGGCTTTA

>JTB_4923304_1

CGAAACAGCAGTGGTTCACTCTCCAACGGTCAC

>JTC_835256_1

GTTTTGTATGGAAAGAACGGTAACTACAAGGAA

>JTB_3754434_1

GTGCCATGCTCCACTTTTACCGTAAGAAGTGTT

>JTC_1611637_1

CTTTAGTATACTTTTGACCGTTGGAGAGTGAAC

>JTC_1058596_1

GTAATCTAGCTGCATGCTGTTTGAAACTCCGTA

>JTB_4049206_1

GGAATTCACAGCATCGATCAAGAAATACGACGA

>JTB_4306660_1

GAGGTTGTTCAACTGTTTTGCCACATCGGGATG

>JTC_420068_1

TTTGAATCGCAGCTAGGTGTTATCGATCCCAAT

>JTC_5036527_1

CAAAACCAAGTGTAATCTAGCTGCATGCTGTTT

>JTC_3013362_2

ATTTGGCCGTTTTGTATGGAAAGAACGGTAACT

>JTC_102066_4

CGATGAGGTACAGAATGGTGACAGTGAATCAAT

>JTB_1365973_1

GCTGTTGGTCGCCTTATTATCTCACCTGAAAAG

>JTC_4138434_1

GATGGATGACACTCACCCAAACAGCGAACCCAT

>JTC_5179968_1

AGACCATGGTCAGGATGCTTAACATTGTTGCCA

>JTC_3028151_2

AAGACAGTCAGCAGCAACTGCTGGAAGCTCCAC

>JTC_3134370_1

TTCTCACGTTCAAGATCTTCCAGGGCTTGCTTA

>JTC_703700_1

TGAAGTGTCTCCACGTCTAAAGACTATTCAAAA

>JTC_3829402_1

GGCCATTCTCACGTTCAAGATCTTCCAGGGCTT

>JTB_3093264_2

CCGCTTCCTTGTAGTTACCGTTCTTTCCATACA

>JTC_2887096_2

TATCGGCCAAGAACGTTTTCACGATTAGCCAAA

>JTC_3885856_1

GGACTTTGTCAGGAAACCTCCTGGCTACGGGAC

>JTC_3692792_1

GTCAAATGCTGGATCATCACCGAACTTCTCGTC

>JTB_795796_1

TCCTGCGTTTTGACCAACACGTCTCTTAACAAC

>JTB_1616197_1

GAAATGCGCAGAACATCGAACTAGGATTGGGAG

>JTC_2486472_1

ATCGATCAAGAAATACGACGACTTTTTGGAAGG

>JTB_5245211_1

ATTACACTTGGTTTTGATGGCATTGGGATCGAT

>JTB_225902_2

GTTTGCTCCGGAACTGACCGTGTTTCAGCTTGG

>JTC_2690378_1

AAACAGCAGTGGTTCACTCTCCAACGGTCACCA

>JTC_614621_1

TGGAGCTGTACATTCGAAGAGCGTTGGAAATTT

>JTB_1276708_1

GGAGAGTGAACCACTGCTGTTTCGTGCCATGCT

>JTC_4872928_1

CCCAATGCCATCAAAACCAAGTGTAATCTAGCT

>JTC_4102006_1

GCAGACTCTCGTCGGTCATCAGCAACGTTGATT

>JTC_4716276_1

CGAGAAGTTCGGTGATGATCCAGCATTTGACCT

>JTC_103891_4

CCGTTGTGTAAGCAAGCCCTGGAAGATCTTGAA

>JTC_958660_1

GTGTAATCTAGCTGCATGCTGTTTGAAACTCCG

>JTC_2482101_1

ATCTAGCTGCATGCTGTTTGAAACTCCGTAAGT

>JTC_2543314_1

AGCATCGATCAAGAAATACGACGACTTTTTGGA

>JTC_3442330_1

TAGGTGTTATCGATCCCAATGCCATCAAAACCA

>JTC_2875015_2

TGGAGCTGTACATTCGAAGAGCGTTGGAAATTT

>JTC_940086_1

GTTCACTCTCCAACGGTCAAAAGTATACTAACG

>JTC_3495992_1

GTTTGGGTGAGTGTCATCCATCGGTGGCAGCGG

>JTC_304644_2

CGTGTTTCAGCTTGGATGACACGTCAAAGTTGC

>JTC_5080655_1

ATTAGCCAAAGCTCTCTTGCATAAAGATTCCGC

>JTB_1368583_1

GCTGTACATTCGAAGAGCGTTGGAAATTTTTGA

>JTB_1691164_1

CTTCTTCCTTCTCCGGATTGGAGATGGGATCTG

>JTC_3328562_1

TGAGGTACAGAATGGTGACAGTGAATCAATTAA

>JTC_3376294_1

TCTCTTAACAACTGGTCAGCTTCTTTGTACTTA

>JTC_4503874_1

CGGTGTTGGTGTCGACTTGCTAAGTCGTCTTTC

>JTC_987623_1

GTGAACCACTGCTGTTTCGTGCCATGCTCCACT

>JTB_1100523_1

GTAAGTACAAAGAAGCTGACCAGTTGTTAAGAG

>JTC_4905106_1

CATGTGTTTAATGGCTTCGGGGAGGTTATTCTG

>JTB_1780774_1

CTGACCAGTTGTTAAGAGACGTGTTGGTCAAAA

>JTB_4003306_1

GGATCATCACCGAACTTCTCGTCGAATTCTACA

>JTB_3380189_1

TGGAGCTGTACATTCGAAGAGCGTTGGAAATTT

>JTC_1044413_1

GTCAAAGTTGCCCAATCTTTGGTGCAACGAGGC

>JTB_4529130_1

CTGATCTCGGTAGACCATGGTCAGGATGCTTAA

>JTB_541080_1

TTCAACTGTTTTGCCACATCGGGATGATATCGG

>JTC_3062848_1

TTTGGCCGTTTTGTATGGAAAGAACGGTAATTA

>JTC_3917451_1

GGAAGGTGTAGAATTCGACGAGAAGTTCGGTGA

>JTC_1473158_1

GCAAAACAGTTGAACAACCTCGCATTATTATGC

>JTC_4796392_1

CCGTTTTGTATGGAAAGAACGGTAACTACAAGG

>JTC_3889461_1

GGACGGTGTTGGTGTCGACTTGCTAAGTCGTCT

>JTC_872378_1

GTTTCAGCTTGGATGACACGTCAAAGTTGCCCA

>JTC_4899113_1

CCAAAATCTCCTGTTTGCTCCGGAACTGACCGT

>JTB_4601301_1

CTAGATTACACTTGGTTTTGATGGCATTGGGAT

>JTC_2568864_1

AGAATTCGACGAGAAGTTCGGTGATGATCCAGC

>JTB_1533674_1

GATTATGAAGTGTCTCCACGTCTAAAGACTATT

>JTC_1605128_1

CTTTCTGCAATTTCATCTTCGGGGAATAGGTCA

>JTB_4484201_1

CTTAACATTGTTGCCACATCTGGATGTTCACGG

>JTC_1840555_1

CGTCGATGCTTTTCAGGTGAGATAATAAGGCGA

>JTB_3881246_1

GGTCAGGATGCTTAACATTGTTGCCACATCTGG

>JTC_1485513_1

GATGGATGACACTCACCCAAACAGCGAACCCAT

>JTB_5492088_1

AAAAGACAGTCAGCAGCAACTGCTGGAAGCTCA

>JTB_4985381_1

CCGGAACTGACCGTGTTTCAGCTTGGATGACAC

>JTB_2156751_1

CGAATTCCTTATCCTGCGTTTTGACCAACACGT

>JTC_2489784_1

ATCCGATACGGCATCTCCAGTACAGATCCCATC

>JTC_3446495_1

TAGACCATGGTCAGGATGCTTAACATTGTTTCC

>JTC_1637930_1

CTTCTTTGTACTTACGGAGTTTCAAACAGCATG

>JTC_239582_2

GGTCAGGATGCTTAACATTGTTGCCACATCTGG

>JTC_3163745_1

TTCAAACAGCATGCAGCTAGATTACACTTGGTT

>JTC_3743996_1

GGTCAGGATGCTTAACATTGTTGCCACATCTGG

>JTC_5070065_1

ATTCTGATCTCGGTAGACCATGGTCAGGATGCT

>JTB_3464852_1

TGACCATGGTCTACCGAGATCAGAATAACCTCA

>JTC_545955_1

TGTGGAGCATGGCACGAAACAGCAGTGGTTCAC

>JTC_4214391_1

GAAATTGCAGAAAGACGACTTAGCAAGTCGACA

>JTB_417079_1

TTTTGAATCGCAGCTAGGTGTTATCGATCCCAA

>JTC_2145099_1

CCTTGATTTTGGCATAATAATGCGAGGTTGTTC

>JTC_4826842_1

CCGGAACTGACCGTGTTTCAGCTTGGATGACCC

>JTC_3377686_1

TCTCGGTAGACCATGGTCAGGATGCTTAACATT

>JTB_70872_5

CGGAATCTTTATGCAAGAGAGCTTTGGCTAATC

>JTC_2388869_1

CAACTGGTCAGCTTCTTTGTACTTACGGAGTTT

>JTB_2926415_3

AATAGGTCAAATGCTGGATCATCACCGAACTTC

>JTB_1365206_1

GCTTAACATTGTTGCCACATCTGGATGTTCACG

>JTB_873251_1

GTTTTGACCAACACGTCTCTTAACAACTGGTCA

>JTB_1137343_1

GGTCAAAACGCAGGATAAGGAATTCGATGAGGT

>JTB_2570840_1

AGGATGCTTAACATTGTTGCCACATCTGGATGT

>JTB_4657388_1

CGGTGTTGGTGTCGACTTGCTAAGTCGTCTTTC

>JTC_3662361_1

GTCGCAGTACCGTTGTGTAAGCAAGCCCTGGAA

>JTB_4486016_1

CTGTTGGTCGCCTTATTATCTCACCTGAAAAGC

>JTB_2531951_1

ATCGAATTCCTTATCCTGCGTTTTGACCAACAC

>JTC_5119218_1

ATAACACCTAGCTGCGATTCAAAAATTTCCAAC

>JTC_205617_2

TGAACATCCAGATGTGGCAACAATGTTAAGCAT

>JTC_1816513_1

CGTTGTGTAAGCAAGCCCTGGAAGATCTTGAAC

>JTC_1915126_1

CGGCTTTGAATAATTTGGCCGTTTTGTATGGCA

>JTC_2361230_1

CACAGAATGGTGACAGTGAATCAATTAACGTCG

>JTB_1914234_1

CGGTGATGATCCAGCATTTGACCTATTCCCCGA

>JTB_3381630_1

TGGAGAGTGAACCACTGCTGTTTCGTGCCATGC

>JTB_3658420_1

GTTGTTCAACTGTTTTGCCACATCGGGATGATA

>JTC_1240349_1

GGACGGTGTTGGTGTCGACTTGCTAAGTCGCCT

>JTC_471841_1

TTGACTTGTATGCGAAGCGTTTGCCGTTCAACG

>JTC_5204504_1

ACTTTTTGGAAGGTGTAGAATTCGACGAGACGT

>JTB_5340982_1

AGATAATAAGGCGACCAACAGCTGAGCTTCTCC

>JTB_1578536_1

GACCTATTCCCCGAAGATGAAATTGCAGAAAGA

>JTC_3107967_1

TTGGGATCGATAACACCTAGCTGCGATTCAACA

>JTB_110744_3

TTTTGATGGCATTGGGATCGATAACACCTAGCT

>JTC_943139_1

GTTCAACATCGATGCTTTTCAGGTGAGATAATA

>JTB_3698770_1

GTTCGGTGATGATCCAGCATTTGACCTATTCCC

>JTC_4207885_1

GAACGGCAAACGCTTCGCATACAAGTCAAAGGA

>JTC_149442_3

CTGATCTCGGTAGACCATGGTCAGGATGCTTAA

>JTB_462290_1

TTTCACGATTAGCCAAAGCTCTCTTGCATAAAG

>JTB_1706814_1

CTTCCTTCTCCGGATTGGAGATGGGATCTGCAA

>JTC_318076_2

CGGAACTGACCGTGTTTCAGCTTGGATGACACG

>JTC_3513348_1

GTTTCCTGTACCACACTCAACGTTGCTGATGAC

>JTB_4234891_1

GCCAAGTGTAATCTAGCTGCATGCTGTTTGAAA

>JTB_1626969_1

GAAACAGCAGTGGTTCACTCTCCAACGGTCAAA

>JTB_808131_1

TCCAGATGTGGCAACAATGTTAAGCATCCTGAC

>JTC_1632617_1

CTTGATCGATGCTGTGAATTCCATCTGTCTCTT

>JTC_4741290_1

CGAATTCCTTATCCTGCGTTTTGACCAACACGT

>JTC_4755781_1

CGAACTAGGATTGGGAGAAGCTCAGCTGTTGGC

>JTB_4052910_1

GGAATCTTTATGCAAGAGAGCTTTGGCTAATCG

>JTC_3774019_1

GGGGAATAGGTCAAATGCTGGATCATCACCGAA

>JTC_1661579_1

CTTCCAGGGCTTGCTTACACAACGGTACTGCGA

>JTC_1595991_1

CTTTTACCGTAAGAAGTGTTATCAACGTTAATT

>JTB_4306631_1

GAGGTTTCCTGACAAAGTCCTTTGACTTGTATG

>JTB_3080713_2

CGATATCATCCCGATGTGGCAAAACAGTTGAAC

>JTC_1320171_1

GCTTAACATTGTTGCCACATCTGGATGTTCACG

>JTC_2651381_1

AAGATATGAAGTCGCAGTACCGTTGTGTAAGCA

>JTC_4078009_1

GCCATATGCTTTAGTATACTTTTGACCGTTGGA

>JTC_3571036_1

GTTCGCTGTTTGGGTGAGTGTCATCCATCGGTG

>JTB_594870_1

TGTAAGCAAGCCCTGGAAGATCTTGAACGTGAG

>JTB_2501295_1

ATTCGAAGAGCGTTGGAAATTTTTGAATCGCAG

>JTC_2164892_1

CCTCATCGAATTCCTTATCCTGCGTTTTGACCA

>JTC_5156733_1

AGCCAGGAGGTTTCCTGACAAAGTCCTTTGACT

>JTC_3022179_2

AGACAGTCAGCAGCAACTGCTGGAAGCTCAACT

>JTB_3986363_1

GGCAAAACAGTTGAACAACCTCGCATTATTATG

>JTB_1315437_1

GGAAGCGCTACGTACTGAGCTGCAGTCTATCCG

>JTC_4219296_1

GAAATACGACGACTTTTTGGAAGGTGTAGAATT

>JTC_1406940_1

GCCGTATCGGATAGACTGCAGCTCAGTACGTAG

>JTB_706407_1

TGATGATCCAGCATTTGACCTATTCCCCGACGC

>JTB_4375751_1

GAAATACGACGACTTTTTGGAAGGTGTAGAATT

>JTB_744780_1

TGAACCACTGCTGTTTCGTGCCATGCTCCACTT

>JTC_3467399_1

TAAAAGACAGTCAGCAGCAACTGCTGGAAGCTC

>JTB_3526575_1

TCGACGAGAAGTTCGGTGATGATCCAGCATTTG

>JTC_2948790_2

CTTTTTGGAAGGTGTAGAATTCGACGAGAAGTT

>JTB_4584310_1

CTCCAATCCGGAGAAGGAAGAAGTCATCAATCG

>JTB_3106493_2

CACAGAATGGTGACAGTGAATCAATTAACGTCG

>JTC_4962799_1

CACGGTGAGGTGGAGCTGTACATTCGAAGAGCG

>JTB_2880633_3

GTCAAATGCTGGATCATCACCGAACTTCTCGTC

>JTC_3973424_1

GCTTAACATTGTTGCCACATCTGGATGTTCAAG

>JTC_2458433_1

ATTCGAAGAGCGTTGGAAATTTTTGAATCGCAG

>JTD_3387897_1

TGGAGCTGTACATTCGAAGAGCGTTGGAAATTT

>JTC_3685670_1

GTCAGCAGCAACTGCTGGAAGCTCAACTTAAAA

>JTC_385996_1

TTTTTGAATCGCAGCTAGGTGTTATCGATCCCA

>JTC_1631311_1

CTTGATTTTGGCATAATAATGCGAGGTTGTTCA

>JTB_2317456_1

CCAATCCGGAGAAGGAAGAAGTCATCAATCGAA

>JTC_3719385_1

GGTTGTTCAACTGTTTTGCCACATCGGGATGAT

>JTC_1607519_1

CTTTCCTTGATTTTGGCATAATAATGCGAGGTT

>JTC_762625_1

TCCTCACCGTGCTTTCCTTGATTTTGGCATAAT

>JTC_3390115_1

TCGGGATGATATCGGCCAAGAACGTTTTCACGA

>JTC_3017435_2

ATATGAAGTCGCAGTACCGTTGTGTAAGCAAGC

>JTB_3440973_1

TGATGATCCAGCATTTGACCTATTCCCCGAAGA

>JTB_4064669_1

GGAAGCGCTACGTACTGAGCTGCAGTCTATCCG

>JTC_4222695_1

GAAAGACAGTCAGCAGCAACTGCTGGAAGCTCA

>JTC_292731_2

CTGGGACGGTGTTGGTGTCGACTTGCTAAGTCG

>JTC_2508384_1

AGTTGAACAACCTCGCATTATTATGCCAAAATC

>JTB_2496974_1

ATTCTGATCTCGGTAGACCATGGTCAGGATGCT

>JTC_4454586_1

CTAAGTCGTCTTTCTGCAATTTCATCTTCGGGG

>JTB_4842658_1

CGATTATGAAGTGTCTCCACGTCTAAAGACTAT

>JTB_5071484_1

CATCGATCAAGAAATACGACGACTTTTTGGAAG

>JTC_204287_2

TGACAGTGAATCAATTAACGTTGATAACACTTC

>JTC_752520_1

TCGCAGTACCGTTGTGTAAGCAAGCCCTGGAAG

>JTB_2594475_1

AGATTCCGCTTCCTTGTAGTTACCGTTCTTTCC

>JTC_5013652_1

CAACAGCTGAGCTTCTCCCAATCCTAGTTCGAT

>JTB_1209496_1

GGCGGGCTTGCTTACACAACGGTACTGCGACTT

>JTC_835255_1

GTTTTGTATGGAAAGAACGGTAACTACAAGGCA

>JTC_348527_2

CAGCAGTGGTTCACTCTCCAACGGTCAAAAGTA

>JTC_3629627_1

GTGAGAATGGCCGTGAACATCCAGATGTGGCAA

>JTC_3045833_1

TTTTGACCAACACGTCTCTTAACAACTGGTCAG

>JTC_1334044_1

GCTGACTGTCTTTCACTTCATCCCGTAGCCCGG

>JTC_1993475_1

CGCTTCCAGGCTTTGCTTCACTGTCTTCATTTT

>JTC_675540_1

TGATCTCGGTAGACCATGGTCAGGATGCTTACC

>JTC_1144565_1

GGGACGGTGTTGGTGTCGACTTGCTAAGTCGTC

>JTB_3547238_1

TCATCCCGTAGCCAGGAGGTTTCCTGACAAAGT

>JTC_4444600_1

CTAGTTCGATGTTCTGCGCATTTCGATTGATTA

>JTC_1715319_1

CTGCGATTCAAAAATTTCCAACGCTCTTCGAAT

>JTB_2730987_1

AAAGAAGCTGACCAGTTGTTAAGAGACGTGTTG

>JTB_304042_2

CTTCCTTGTAGTTACCGTTCTTTCCATACAAAA

>JTB_2956582_2

TGACAGTGAATCAATTAACGTTGATAACACTTC

>JTB_1655661_1

CTTTGAATAATTTGGCCGTTTTGTATGGAAAGA

>JTC_195952_2

TGGCCGTTTTGTATGGAAAGAACGGTAACTACA

>JTB_2968037_2

GTTTTGTATGGAAAGAACGGTAACTACAAGGAA

>JTB_4105605_1

GCTTTCCTTGATTTTGGCATAATAATGCGAGGT

>JTC_190366_2

TGTTGGTGTCGACTTGCTAAGTCGTCTTTCTGC

>JTC_4484973_1

CGTCGATGCTTTTCAGGTGAGATAATAAGGCGC

>JTC_4943647_1

CAGATGTGGCAACAATGTTAAGCATCCTGACCA

>JTB_5206308_1

ATTTTGGAGAGCAGACTCTCGTCGGTCATCAGC

>JTB_765851_1

TCTCGGTAGACCATGGTCAGGATGCTTAACATT

>JTC_2491533_1

ATCCAAAATCTCCTGTTTGCTCCGGAACTGACC

>JTC_4182251_1

GACACTTCATAATCGGGATAGTGCTGGGACGGT

>JTC_5170936_1

AGATAATAAGGCGACCAACAGCTGAGCTTCTCC

>JTC_2982897_2

CGCAGGATAAGGAATTCGATGAGGTACAGAATG

>JTB_4629916_1

CGTGCTCCACTTTTACCGTAAGAAGTGTTATCG

>JTC_4797025_1

CCGTTGTGTAAGCAAGCCCTGGAAGATCTTGAA

>JTC_1788609_1

CTCACGTTCAAGATCTTCCAGGGCTTGCTTACA

>JTB_4361327_1

GAACTTCTCGTCGAATTCTACACCTTCCAAAAA

>JTC_3062703_1

TTTGGCTAATCGTGAAAACGTTCTTGGCCGATA

>JTC_5313864_1

AAAAGACAGTCAGCAGCAACTGCTGGAAGCTCA

>JTC_4233725_1

GAAAAGAGACAGATGGAATTCACAGCATCGATC

>JTB_1745322_1

CTGGGACGGTGTTGGTGTCGACTTGCTAAGTCG

>JTB_559451_1

TGTTGGTGTCGACTTGCTAAGTCGTCTTTCTCC

>JTB_2169884_1

CGAACTTCTCGTCGAATTCTACACCTTCCAAAA

>JTC_1535119_1

GAATTCCTTATCCTGCGTTTTGACCAACACGTC

>JTB_2214258_1

CCGTGGGTGAGTGTCATCCATCGGTGGCAGCGG

>JTC_2880864_2

TGACAGTGAATCAATTAACGTTGATAACACTTC

>JTC_1278959_1

GGAACTGACCGTGTTTCAGCTTGGATGACACGT

>JTC_2333669_1

CAGAATGGTGACAGTGAATCAATTAACGTTGAT

>JTC_4056022_1

GCCGTTTTGTATGGAAAGAACGGTAACTACAAG

>JTC_1712362_1

CTGCTCTCCAAAATGAAGACAGTGAAGCAAAGC

>JTC_208395_2

TCGGGATGATATCGGCCAAGAACGTTTTCACGA

>JTB_3820611_1

GTCAGGATGCTTAACATTGTTGCCACATCTGGA

>JTB_453399_1

TTTCGATTGATGACTTCTTCCTTCTCCGGATTG

>JTC_3348886_1

TGAAGTCGCAGTACCGTTGTGTAAGCAAGCCCT

>JTB_4439265_1

CTTGCATAAAGATTCCGCTTCCTTGTAGTTACC

>JTC_5183522_1

AGAATGGTGACAGTGAATCAATTAACGTTGATA

>JTC_4510776_1

CGGTGATGATCCAGCATTTGACCTATTCCCCGA

>JTC_843569_1

GTTTTCACGATTAGCCAAAGCTCTCTTGCATAG

>JTC_134235_3

GGAGAGTGAACCACTGCTGTTTCGTGCCATGCT

>JTB_1878195_1

CGTGCTTTCCTTGATTTTGGCATAATAATGCGC

>JTC_2410301_1

CAAAGCCTGGAAGCGCTACGTACTGAGCTGCCG

>JTC_5081982_1

ATTACACTTGGTTTTGATGGCATTGGGATCGAT

>JTC_3071138_1

TTTGAATAATTTGGCCGTTTTGTATGGAAAGAA

>JTB_2644365_1

ACGGTGTTGGTGTCGACTTGCTAAGTCGTCTTT

>JTB_1906727_1

CGGTGTTGGTGTCGACTTGCTAAGTCGTCTTTC

>JTB_350464_2

CGAAACAGCAGTGGTTCACTCTCCAACGGTCAA

>JTC_2258229_1

CCAGTACAGATCCCATCTCCAATCCGGAGAAGG

>JTC_4190960_1

GAATCGCAGCTAGGTGTTATCGATCCCAATGCC

>JTB_242822_2

GTCGACTTGCTAAGTCGTCTTTCTGCAATTTCA

>JTB_1365662_1

GCTGTTTGAAACTCCGTAAGTACAAAGAAGCTG

>JTC_2699805_1

AAAAGACAGTCAGCAGCAACTGCTGGAAGCTCA

>JTC_2254623_1

CCATGCTCCACTTTTACCGTAAGAAGTGTTATC

>JTC_940087_1

GTTCACTCTCCAACGGTCAAAAGTATACTAAAG

>JTB_5494214_1

AAAACGCAGGATAAGGAATTCGATGAGGTACAG

>JTC_4733471_1

CGACCAACAGCTGAGCTTCTCCCAATCCTAGTT

>JTB_3287702_1

TTACACTTGGTTTTGATGGCATTGGGATCGTTA

>JTC_703887_1

TGAAGTCGCAGTACCGTTGTGTAAGCAAGCCCT

>JTB_4678310_1

CGGGTGAGTGTCATCCATCGGTGGCAGCGGCCC

>JTC_1919966_1

CGGCGTCGTATTTCTTGATCGATGCTGTGAATT

>JTC_1055877_1

GTACATTCGAAGAGCGTTGGAAATTTTTGAATC

>JTC_2650939_1

AAGATCTTGAACGTGAGAATGGCCGTGAACCTC

>JTC_880927_1

GTTGTTTCGTGCCATGCTCCACTTTTACCGTAA

>JTC_5073727_1

ATTCGACGAGAAGTTCGGTGATGATCCAGCATT

>JTC_4254223_1

CTTTGCTTCACTGTCTTCATTTTGGAGAGCAGA

>JTC_804598_1

TAGAATTCGACGAGAAGTTCGGTGATGATCCAG

>JTC_3478032_1

GTTTTGTATGGAAAGAACGGTAACTACAAGCAA

>JTB_2359649_1

CAGCTTCTTTGTACTTACGGAGTTTCAAACAGC

>JTC_3486877_1

GTTTTCACGATTAGCCAAAGCTCTCTTGCATAA

>JTC_3685198_1

GTCAGCTTCTTTGTACTTACGGAGTTTCAAACA

>JTC_3645629_1

GTCTCATCGAATTCCTTATCCTGCGTTTTGACC

>JTC_177341_3

AAAAAGTCGTCGTATTTCTTGATCGATGCTGTG

>JTC_286884_2

CTTGAACGTGAGAATGGCCGTGAACATCCAGAT

>JTB_750529_1

TGAAAACGTTCTTGGCCGATATCATCCCGATGT

>JTC_698079_1

TGACAGTGAATCAATTAACGTTGATAACACTTT

>JTB_958141_1

GTTCGGTGATGATCCAGCATTTGACCTATTCCC

>JTC_3666227_1

GTCGACTTTTTGGAAGGTGTAGAATTCGACGAG

>JTC_741129_1

TCGTCTTTCTGCAATTTCATCTTCGGGGAATAG

>JTC_3002598_2

CAGTACCGTTGTGTAAGCAAGCCCTGGAAGATC

>JTB_1857407_1

CTAAGTCGTCTTTCTGCAATTTCATCTTCGGGG

>JTC_820298_1

TAACATTGTTGCCACATCTGGATGTTCACGGCC

>JTC_3415481_1

TCCAGGCTTTGCTTCACTGTCTTCATTTTGGAG

>JTC_5090397_1

ATGATATCGGCCAAGAACGTTTTCACGATTAGC

>JTB_2937327_2

TTGAATAGTCTTTAGACGTGGAGACACTTCATA

>JTB_2978116_2

GTTCGATGTTCTGCGCATTTCGATTGATGACTT

>JTB_2191646_1

CCTTATCCTGCGTTTTGACCAACACGTCTCTTA

>JTB_2337267_1

CATCGATCAAGAAATACGACGACTTTTTGGAAG

>JTB_3259148_1

TTCGAAGAGCGTTGGAAATTTTTGAATCGCAGC

>JTC_2925156_2

GGAACTGACCGTGTTTCAGCTTGGATGACACGT

>JTC_944776_1

GTTATTCTGATCTCGGTAGACCATGGTCAGGAT

>JTB_2715864_1

AACAAGTGTAATCTAGCTGCATGCTGTTTGAAA

>JTB_941333_1

GTTGATAACACTTCTTACGGTAAAAGTGGAGCA

>JTC_258032_2

GCTTTGAATAATTTGGCCGTTTTGTATGGAAAG

>JTC_4446481_1

CTAGATTACACTTGGTTTTGATGGCATTGGGAT

>JTB_3057105_2

CTCCACTTTTACCGTAAGAAGTGTTATCAACGT

>JTC_2950880_2

CTTTAGTATACTTTTGACCGTTGGAGAGTGAAC

>JTB_4774514_1

CGGAACTGACCGTGTTTCAGCTTGGATGACACG

>JTC_2833187_3

CTTGAACGTGAGAATGGCCGTGAACATCCAGAT

>JTC_1149534_1

GGGAATAGGTCAAATGCTGGATCATCACCGAAC

>JTB_2433436_1

CAACTGGTCAGCTTCTTTGTACTTACGGAGTTT

>JTB_3086070_2

CGAAGATGAAATTGCAGAAAGACGACTTAGCAA

>JTC_2995683_2

CCGGAACTGACCGTGTTTCAGCTTGGATGACAC

>JTC_4787147_1

CCTCGTTGCACCAAAGATTGGGCAACTTTGACG

>JTB_1124060_1

GGTGGAGCTGTACATTCGAAGAGCGTTGGAAAT

>JTC_1184403_1

GGCCAAGAACGTTTTCACGATTAGCCAAAGCTC

>JTC_4290633_1

CTTCTTTGTACTTACGGAGTTTCAAACAGCATG

>JTC_3744266_1

GGTCAGCAGCAACTGCTGGAAGCTCAACTTAAA

>JTB_5095325_1

CAGCTAGATTACACTTGGTTTTGATGGCATTGG

>JTC_473585_1

TTGAATCGCAGCTAGGTGTTATCGATCCCAATT

>JTC_2941017_2

GATCTCGGTAGACCATGGTCAGGATGCTTAACA

>JTB_2469615_1

ATTTTTGAATCGCAGCTAGGTGTTATCGATCCC

>JTC_3271292_1

TGGAAGGTGTAGAATTCGACGAGAAGTTCGGTG

>JTB_512687_1

TTCGTGCCATGCTCCACTTTTACCGTAAGAAGT

>JTC_307659_2

CGGTGTTGGTGTCGACTTGCTAAGTCGTCTTTC

>JTB_2365855_1

CAGCATTTGACCTATTCCCCGAAGATGAAATTG

>JTC_3635908_1

GTGAACCACTGCTGTTTCGTGCCATGCTCCACT

>JTC_3941298_1

GGAAATTTTTGAATCGCAGCTAGGTGTTATCGC

>JTC_4387452_1

CTCTTGCATAAAGATTCCGCTTCCTTGTAGTTA

>JTB_1304155_1

GGAATCTTTATGCAAGAGAGCTTTGGCTAATCG

>JTC_3313314_1

TGATTCACTGTCACCATTCTGTACCTCATCGAA

>JTC_3986981_1

GCTGACTGTCTTTCACTTCATCCCGTAGCCAGG

>JTB_4923305_1

CGAAACAGCAGTGGTTCACTCTCCAACGGTCAA

>JTC_473586_1

TTGAATCGCAGCTAGGTGTTATCGATCCCAATG

>JTC_3714438_1

GTAAAAGACAGTCAGCAGCAACTGCTGGAAGCT

>JTB_5101998_1

CAGCACTATCCCGATTATGAAGTGTCTCCACGT

>JTC_675541_1

TGATCTCGGTAGACCATGGTCAGGATGCTTAAC

>JTB_829102_1

TATCGATCCCAATGCCATCAAAACCAAGTGTAA

>JTC_4166582_1

GAGAAGCTCAGCTGTTGGTCGCCTTATTATCTC

>JTC_3505880_1

GTTTGAAACTCCGTAAGTACAAAGAAGCTGACC

>JTC_4547038_1

CGGGATAGTGCTGGGACGGTGTTGGTGTCGACT

>JTC_537625_1

TGTTCAACTGTTTTGCCACATCGGGATGATATC

>JTB_3639905_1

GTTTCGTGCCATGCTCCACTTTTACCGTAAGAA

>JTC_4470044_1

CGTGTTTCAGCTTGGATGACACGTCAAAGTTGC

>JTC_495034_1

TTCGACGAGAAGTTCGGTGATGATCCAGCATTT

>JTC_463772_1

TTGCTAAGTCGTCTTTCTGCAATTTCATCTTCG

>JTC_1052422_1

GTAGAATTCGACGAGAAGTTCGGTGATGATCCA

>JTC_985238_1

GTGAATCAATTAACGTTGATAACACTTCTTACG

>JTC_217382_2

GTTTGGGTGAGTGTCATCCATCGGTGGCAGCGG

>JTC_2952566_2

CTTGATTTTGGCATAATAATGCGAGGTTGTTCA

>JTC_4275336_1

CTTGGGTATATGGGTTCGCTGTTTGGGTGAGTG

>JTC_984809_1

GTGAATTCCATCTGTCTCTTTTCCTCTTCGAGC

>JTC_1017973_1

GTCGACTTGCTAAGTCGTCTTTCTGCAATTTCC

>JTC_3366402_1

TCTTGATCGATGCTGTGAATTCCATCTNTCTCT

>JTC_3529609_1

GTTGTTAAGAGACGTGTTGGTCAAAACGCAGGA

>JTC_1566205_1

GAAATACGACGACTTTTTGGAAGGTGTAGAATT

>JTC_606226_1

TGGCAAAACAGTTGAACAACCTCGCATTATTAT

>JTC_2175272_1

CCGTATCGGATAGACTGCAGCTCAGTACGTAGC

>JTB_1837922_1

CTCACGTTCAAGATCTTCCAGGGCTTGCTTACA

>JTC_3529259_1

GTTGTTCAACTGTTTTGCCACATCGGGATGATA

>JTC_3380539_1

TCTCCAACGGTCAAAAGTATACTAAAGCATATG

>JTB_4250547_1

GCAGCATCGATCAAGAAATACGACGACTTTTTG

>JTC_958734_1

GTGTAAGCAAGCCCTGGAAGATCTTGAACGTGA

>JTC_5175097_1

AGAGAGCTTTGGCTAATCGTGAAAACGTTCTTG

>JTC_2316173_1

CAGCTAGGTGTTATCGATCCCAATGCCATCAAA

>JTB_4884812_1

CGACGAGAAGTTCGGTGATGATCCAGCATTTGA

>JTB_3548707_1

TCAGGATGCTTAACATTGTTGCCACATCTGGAT

>JTB_4491532_1

CTGTACATTCGAAGAGCGTTGGAAATTTTTGAA

>JTC_4099296_1

GCAGCATCGATCAAGAAATACGACGACTTTTTG

>JTC_1598593_1

CTTTGGTGCAACGAGGCCATATGCTTTAGTATA

>JTB_1438806_1

GCGACCAACAGCTGAGCTTCTCCCAATCCTAGT

>JTB_5117378_1

CAGAAAGACGACTTAGCAAGTCGACACCAACAC

>JTB_4470195_1

CTTCATTCATGTGTTTAATGGCTTCGGGGAGGT

>JTB_341484_2

CGATTATGAAGTGTCTCCACGTCTAAAGACTAT

>JTC_4965778_1

CACGATTAGCCAAAGCTCTCTTGCATAAAGATT

>JTC_1744520_1

CTCTCGTCGGTCATCAGCAACGTTGAGTGTGGT

>JTB_706408_1

TGATGATCCAGCATTTGACCTATTCCCCGAAGA

>JTC_2517599_1

AGGTTATTCTGATCTCGGTAGACCATGGTCAGG

>JTC_4421067_1

CTCCGGAACTGACCGTGTTTCAGCTTGGATGAC

>JTC_2514086_1

AGTCAGCAGCAACTGCTGGAAGCTCAACTTAAA

>JTC_997231_1

GTCTCATCGAATTCCTTATCCTGCGTTTTGACC

>JTB_212767_2

TGAACATCCAGATGTGGCAACAATGTTAAGCAT

>JTC_2510224_1

AGTGTCTCCACGTCTAAAGACTATTCAAAACTT

>JTC_5260352_1

AAGGAAGCGGAATCTTTATGCAAGAGAGCTTTT

>JTC_4857197_1

CCCGTTGGAGAGTGAACCACTGCTGTTTCGTGC

>JTC_4058207_1

GCCGTGAACATCCAGATGTGGCAACAATGTTAA

>JTC_556312_1

TGTCGACTTGCTAAGTCGTCTTTCTGCAATTTC

>JTB_4501375_1

CTGGGACGGTGTTGGTGTCGACTTGCTAAGTCG

>JTC_2088259_1

CGACTTGCTAAGTCGTCTTTCTGCAATTTCATC

>JTB_4333613_1

GACCGTTGGAGAGTGAACCACTGCTGTTTCGTG

>JTB_5236861_1

ATTCGAAGAGCGTTGGAAATTTTTGAATCGCAG

>JTC_1800436_1

CTAGATTACACTTGGTTTTGATGGCATTGGGAT

>JTB_2624251_1

AGAACGTTTTCACGATTAGCCAAAGCTCTCTTT

**3) Illumina reads that matched AAEL012472**

>JTC_679696_1

TGATAGCTATGACGAAGAGGGAAAGCGATCTCC

>JTC_1457267_1

GCACAAAGCAGCTAAAGTTGACTCTCCTACTCT

>JTC_3299844_1

TGCCGAGCCGCTTTGCAAGCGAGCTTTGGAAAT

>JTB_321604_2

CGTCTGTAGAGGGCGCCTAGATTCTTCAATGTA

>JTB_3836679_1

GTAGAGGGCGCCTAGATTCTTCAATGTTGTCGT

>JTB_2848352_4

CTGATTTGGTGGTGTTGGGGACATGGTATGTCT

>JTC_296747_2

CTCTTCCCGGAAGATGAGTCCGAGGAGAGACAT

>JTB_61559_5

GTTGGCACAAAGCAGCTAAAGTTGACTCTCCTA

>JTD_482845_1

GTCCCACTTTGCAAACAAGCTCTGGAGGATTTG

>JTD_5545788_1

CATCCTTGTATTTGCCACGTTTACCATAAAGCA

>JTD_5393503_1

CGGCATCCTTGTATTTGCCACGTTTACCATAAA

>JTD_4558127_1

AATTTGCTTAATGATGCGCTTGCAATTCGTGCA

>JTB_4609425_1

CTAAGGTATCAGCAGCCTCGTACTTTCCTTGTC

>JTB_3463272_1

TGACGACAACATTGAAGAATCTAGGCGCCCTCT

>JTB_4143019_1

GCTATCTGAAACAAGGCAAATATAAGGAAGCTG

>JTB_3058089_2

CTCAAGCGCGCTGGGATCTCATAGCCTGCATTG

>JTB_174301_3

ATTTGGTGGTGTTGGGGACATGGTATGTCTCTC

>JTB_732018_1

TGACACAAAAGTGCTAAATTGTTCAGTTGTTTT

>JTD_1989910_1

TCCTTGTATTTGCCACGTTTACCATAAAGCACA

>JTB_4413282_1

CTTTGACGTCTGTTTTCGTCGGACACTCCCAGG

>JTB_1787892_1

CTCTTTCTTCGGCAACCTGCCAGATAGGCTTGT

>JTC_3486875_1

GTTTTCACGATTTTTCGATTTGTTCTCTTCTCT

>JTB_84667_4

GTTCAACATAGTTGCGACGTCCGGATGATCGTG

>JTB_1526026_1

GATTTGTTCTCTTCTCTTTCTTCGGCAACCTGC

>JTB_5213822_1

ATTTGGAGAGAACCAGCGGTCACGATCATCCGG

>JTB_3949618_1

GGCTCGTACTTTCCTTGTCGTCTGTAGAGGGCG

>JTB_4985205_1

CCGGAAGATGAGTCCGAGGAGAGACATACCATG

>JTB_4564113_1

CTCGCAGCCAGGCATTTTCCTGACATAATCGTC

>JTB_2491711_1

ATTGGCGTGGTTTGCAAACTGATTTGGTGGTGT

>JTC_3083222_1

TTTCCTTGTCGTCTGTAGAGGGCGCCTAGATTC

>JTB_312028_2

CTGAAATTCTGTACAAGCAGGTTCTAACTCGTG

>JTB_4131201_1

GCTGATACCTTAGAGGATTGCGCCCTACGCAGC

>JTC_2669013_1

AACAAGCTCTGGAGGATTTGGAGAGAACCAGCG

>JTB_756133_1

TCTTCCTCAAGCTGGGCAACCGTCTGCTCTGAC

>JTA_1697497_3

CTTCCTTATATTTGCCTTGTTTCAGATAGCAGC

>JTC_1429587_1

GCCACGTTTACCATAAAGCACAGCTAGATTGTT

>JTB_2595339_1

AGATGTATTACCAAAGAGCCTTGGAAATATACG

>JTB_118508_3

TGCAAATTTGCTTAATGATGCGCTTGCAATTCG

>JTC_3597413_1

GTTACGTACACAGGTGCGACGATTATGTCAGGA

>JTB_3431415_1

TGCAAATTTGCTTAATGATGCGCTTGCAATTCG

>JTC_463431_1

TTGCTCTTCATTTTCTTGGTTATCGTCATATTT

>JTD_3255710_2

CAGGTGCGACGATTATGTCAGGAAAATGCCTGG

>JTB_580735_1

TGTCGTGCTTGCATCTCATCTACAAAATATCGA

>JTC_251631_2

GGACAGTGATAGCTATGACGAAGAGGGAAAGCG

>JTB_4149258_1

GCGTGGTTTGCAAACTGATTTGGTGGTGTTGGG

>JTB_146374_3

GAAACATCTGGAGTTCATGGCATCCGTGAAGAA

>JTB_466930_1

TTTAGCTGCTTTGTGCCAACCGCCATATTCTCC

>JTB_564238_1

TGTTCTTGGTCTTGGCGACGTTTGGATCATCAG

>JTC_822976_1

TAAAGCCAGGATGTTCAACATAGTTGCGACGTC

>JTC_2470016_1

ATGGTATGTCTCTCCTCGGACTCATCTTCCGGG

>JTB_190274_2

TTGCAAACAAGCTCTGGAGGATTTGGAGAGAAC

>JTB_1528976_1

GATTGTCGTGCTTGCATCTCATCTACAAAATAT

>JTD_486928_1

GTCAGGAAAATGCCTGGCTGCGAGATGAACTCG

>JTB_4386476_1

GAAAATGCCTGGCTGCGAGATGAACTAGCAAAC

>JTB_25418_10

CGGAAGATGAGTCCGAGGAGAGACATACCATGT

>JTD_1745343_2

GAAGAGAACAAATCGAAAAATCGTGAAAACACA

>JTC_1947904_1

CGGATGCCGAGCCGCTTTGCAAGCGAGCTTTGG

>JTB_690193_1

TGCATTGGCGTGGTTTGCAAACTGATTTGGTGG

>JTB_3029639_2

GATCTCATAGCCTGCATTGGCGTGGTTTGCAAA

>JTB_5391953_1

ACGATAACCAAGAAAATGAAGAGCAACAGGAAA

>JTB_2575680_1

AGGACATTGCACAATCTGGTTATCCAGTACGCG

>JTC_5137200_1

AGGCTGCAAATTTGCTTAATGATGCGCTTGCCA

>JTB_2553025_1

AGTTGACTCTCCTACTGTGACGACAACATTGAA

>JTA_197193_2

GCGGTTGGCACAAAGCAGCTAAAGTTGACTCTC

>JTC_5006664_1

CAACGGTGAAAACAAGCCTATCTGGCAGGTTGC

>JTC_2282528_1

CATTGGCGTGGTTTGCAAACTGATTTGGTGGTG

>JTB_2982861_2

GTGATTTTCGCCTAAGGTTTTTTCACGAATTGC

>JTB_3381068_1

TGGAGATGTATTACCAAAGAGCCTTGGAAATAT

>JTB_4804475_1

CGCGCGCTGGGATCTCATAGCCTGCATTGGCGT

>JTB_2670698_1

ACAAAGCAGCTAAAGTTGACTCTCCTACTGTGA

>JTB_4550422_1

CTCTACAGACGACAAGGAAAGTACGAGGCTGCT

>JTC_2366352_1

CACAAAGCAGCTAAAGTTGACTCTCCTACTGTG

>JTD_1255451_1

CATCCGTGAAGAAATATGACGATAACCAAGAAA

>JTC_5175910_1

AGAGAACCAGCGGTCACGATCATCCGGACGTCG

>JTD_1076933_1

CGCTTTCCCTCTTCGTCATAGCTATCACTGTCC

>JTB_2033180_1

CGGAAATTGTTATGAAAAATATTGAAAACATCG

>JTB_2921662_3

ATTTATTCTGATCTCTGTATACTAAAGCCAGGA

>JTA_1874602_2

CACAAAGCAGCTAAAGTTGACTCTCCTACTGTG

>JTB_2820308_5

GATTTTTCCTGTTGCTCTTCATTTTCTTGGTTA

>JTD_2459141_1

CTTATATTTGCCTTGTTTCAGATAGCAGCTTGC

>JTB_1722487_1

CTTATATTTGCCTTGTTTCAGATAGCAGCTTGC

>JTB_5436619_1

AAGCTCTGGAGGATTTGGAGAGAACCAGCGGGC

>JTB_5346189_1

AGAGAACCAGCGGTCACGATCATCCGGACGTCG

>JTD_1494038_1

ACGAAAACAGACGTCAAAGGATGAAGGACAGTG

>JTB_927355_1

GTTGGGGACATGGTATGTCTCTCCTCGGACTCC

>JTB_699376_1

TGATTGTCGTGCTTGCATCTCATCTACAAAATA

>JTD_3216923_2

CTTTGACGTCTGTTTTCGTCGGACACTCCCAGG

>JTB_2718747_1

AAATTGTTCAGTTGTTTTGCAACGTCCGGGTGA

>JTC_355284_2

CAACGGTGAAAACAAGCCTATCTGGCAGGTTGC

>JTB_2862348_3

TTTGCAAACTGATTTGGTGGTGTTGGGGACATG

>JTB_52121_6

CTTCGTCATAGCTATCACTGTCCTTCATCCTTT

>JTD_1159032_1

CCTTGTATTTGCCACGTTTACCATAAAGCACAG

>JTB_5282335_1

ATACGGTGTGTTTTCACGATTTTTCGATTTGTT

>JTB_3595491_1

TAAACGTGGCAAATACAAGGATGCCGAGCCGCT

>JTC_4936684_1

CAGCCTCGTACTTTCCTTGTCGTCTGTAGAGGG

>JTB_2796525_7

GCACAAAGCAGCTAAAGTTGACTCTCCTACTGT

>JTB_3011260_2

GGAAATTGTTATGAAAAATATTGAAAACATCGA

>JTC_556984_1

TGTCCTTCATCCTTTGACGTCTGTTTTCGTCGG

>JTB_1112363_1

GGTTGGCACAAAGCAGCTAAAGTTGACTCTCCT

>JTB_1524791_1

GATTTTTGCCAAGCACATTCTCACGAATTTCCA

>JTB_2658175_1

ACCGCCATATTCTCCATACGGTGTGTTTTCACG

>JTB_3108505_2

CAAGATTGTTCTTGGTCTTGGCGACGTTTGGAT

>JTB_3443552_1

TGATCGTGACCGCTGGTTCTCTCCAAATCCTCC

>JTB_721018_1

TGAGAAGCACAGGTGATTGTCGTGCTTGCATCT

>JTD_4210639_1

CGAAAATCCTGGGAGTGTCCGACGAAAACAGAG

>JTD_1168061_1

CCTGCATTGGCGTGGTTTGCAAACTGATTTGGT

>JTB_313524_2

CTCGTGAGCACGAGTTAGAACCTGCTTGTACAG

>JTC_5209391_1

ACTGAAGGCAGCAAAAAAGACCCAGATAAGTCG

>JTB_46831_6

TGCAAACTGATTTGGTGGTGTTGGGGACATGGT

>JTB_2459433_1

CAAACAAGCTCTGGAGGATTTGGAGAGAACCAG

>JTB_3628501_1

GTTTGCTTAATGATGCGCTTGCAATTCGTGAAA

>JTB_3586144_1

TAATGATGCGCTTGCAATTCGTGAAAAAACCTT

>JTC_4256746_1

CTTTGACGTCTGTTTTCGTCGGACACTCCCAGG

>JTD_2798326_1

CAGATTCTTCAATGTTGTCGTCACAGTAGGAGA

>JTB_1789526_1

CTCTTCGTCATAGCTATCACTGTCCTTCATCCT

>JTB_4110592_1

GCTTGAGGACATTGCACAATCTGGTTATCCAGT

>JTB_5101121_1

CAGCAGCCTCGTACTTTCCTTGTCGTCTGTAGA

>JTD_4989182_1

GTCCCACTTTGCAAACAAGCTCTGGAGGATTTG

>JTB_5072013_1

CATCCTTGTATTTGCCACGTTTACCATAAAGCA

>JTC_2000097_1

CGCTCGTACTTTCCTTGTCGTCTGTAGAGGGCG

>JTB_1353022_1

GCTTTGCAAGCGAGCTTTGGAAATTCGTGAGAA

>JTB_4277572_1

GCAAAAATCACCCGGACGTTGCAAAACAACTGA

>JTB_3811953_1

GTCCCACTTTGCAAACAAGCTCTGGAGGATTTG

>JTD_3403397_1

TGATTTTCGCCTAAGGTTTTTTCACGAATTGCA

>JTC_4230541_1

GAAAATCCTGGGAGTGTCCGACGAAAACAGACG

>JTB_436110_1

TTTGCTTTTCGGCCTCGATATTTTGTAGATGAG

>JTD_3886376_1

CTTTCTTCGGCAACCTGCCAGATAGGCTTGTTT

>JTB_2919159_3

CAATCCTCTAAGGTATCAGCAGCCTCGTACTTT

>JTC_3423341_1

TCACGATCATCCGGACGTCGCAACTATGTTGAA

>JTC_2978786_2

CGGAAGATGAGTCCGAGGAGAGACATACCATGT

>JTC_2960195_2

CTGATCTCTGTATACTAAAGCCAGGATGTTCAA

>JTC_4099006_1

GCAGCCAGGCATTTTCCTGACATAATCGTCGCA

>JTC_1420902_1

GCCCTACGCAGCAAAAAAGAGGCGCTGGATTTA

>JTB_3550601_1

TCACTGTCCTTCATCCTTTGACGTCTGTTTTCG

>JTB_3706011_1

GTTCCTTGTCGTCTGTAGAGGGCGCCTAGATTC

>JTB_156261_3

CGGTCCCACTTTGCAAACAAGCTCTGGAGGATT

>JTC_3794711_1

GGGACCGCTACCTCGTAACGACCTTGAGACGCG

>JTD_3864233_1

GAAACATCTGGAGTTCATGGCATCCGTGAAGAG

>JTC_4359413_1

CTGCTTTGTGCCAACCGCCATATTCTCCATACG

>JTB_493296_1

TTGCAAACTGATTTGGTGGTGTTGGGGACATGG

>JTB_4871907_1

CGAGACTATTTCTTCTTGACTCATTTGCGTCAT

>JTB_2959693_2

TCTGATCTCTGTATACTAAAGCCAGGATGTTCA

>JTB_2145448_1

CGACGAAAACAGACGTCAAAGGATGAAGGACAG

>JTB_2202180_1

CCTCTACAGACGACAAGGAAAGTACGAGGCTGC

>JTB_4397735_1

CTTTTTTGCTGCGTAGGGCGCAATCCTCTAAGG

>JTD_2011846_1

TAGCTATGACGAAGAGGGAAAGCGATCTCAAAA

>JTB_997689_1

GTGGTGGAACTCTTCCCGGAAGATGAGTCCGAG

>JTB_444878_1

TTTCTTGGTTATCGTCATATTTCTTCACGGATG

>JTA_1873160_2

CACCTGTGTACGTAACTTTTGCTTTTCGGCCTC

>JTB_3404060_1

TGCTCTTCATTTTCTTGGTTATCGTCATATTTC

>JTB_4417562_1

CTTTCCTTGTCGTCTGTAGAGGGCGCCTAGATT

>JTB_2938038_2

TTCTGATCTCTGTATACTAAAGCCAGGATGTTC

>JTB_3889181_1

GGTAAACGTGGCAAATACAAGGATGCCGAGCCG

>JTC_950004_1

GTTACGAGGTAGCGGTCCCACTTTGCAAACAAG

>JTB_2013967_1

CGGACGTTGCAAAACAACTGAACAATTTAGCAC

>JTB_3357079_1

TGGGATCTCATAGCCTGCATTGGCGTGGTTTGC

>JTB_5204374_1

ATTTTTCCTGTTGCTCTTCATTTTCTTGGTTAT

>JTB_5184575_1

CAAATACAAGGATGCCGAGCCGCTTTGCAAGCG

>JTB_2732661_1

AAACTGATTTGGTGGTGTTGGGGACATGGTATG

>JTB_133119_3

GGGACATGGTATGTCTCTCCTCGGACTCATCTT

>JTB_5080484_1

CAGTGGTGGAACTCTTCCCGGAAGATGAGTCCG

>JTB_2199652_1

CCTGATCTCTGTATACTAAAGCCAGGATGTTCA

>JTD_2093579_1

GTTAGAACCTGCTTGTACAGAATTTCAGCTTCC

>JTC_5166870_1

AGATGAACTAGCAAACACACAACAGAAGCTCCA

>JTB_1524930_1

GATTTTTCGATTTGTTCTCTTCTCTTTCTTCGG

>JTA_452629_1

TGACACAAAAGTGCTAAATTGTTCAGTTGTTTT

>JTB_383588_2

ATCTCATAGCCTGCATTGGCGTGGTTTGCAAAC

>JTB_1426521_1

GCGCAATCCTCTAAGGTATCAGCAGCCTCGTAC

>JTB_3279255_1

TTATTCTGATCTCTGTATACTAAAGCCAGGATG

>JTC_592084_1

TGGGAGTGTCCGACGAAAACAGACGTCAAAGGA

>JTB_4019866_1

GGAGCGATCAACGGTGAAAACAAGCCTATCTGG

>JTB_2447119_1

CAAATTTGCAGCCTCCTTATATTTATTCTGATC

>JTB_1100254_1

GTAATACATCTCCACTTCTTCATACTTTGTCTG

>JTC_606865_1

TGGATTTAGTAAAGCAGTCCAAGGTTGCGAACA

>JTA_2388246_1

GGAAGCCTTAAGGGTTGAACACTTAACCCTCCT

>JTB_2973447_2

GTTGTTTTGCAACGTCCGGGTGATTTTTGCCAA

>JTB_1300219_1

GGAATTCGGAGCGATCAACGGTGAAAACAAGCC

>JTC_1630478_1

CTTGCATCTCATCTACAAAATATCGAGGCCGAA

>JTC_742924_1

TCGTAACGACCTTGAGACGCGTACTGGATAACC

>JTC_976380_1

GTGATTTTCGCCTAAGGTTTTTTCACGAATTGC

>JTC_5290437_1

AAATCACGCTCGGACCCAGTGGTGGAACTCTTC

>JTB_4937237_1

CCTGGCTTTAGTATACAGAGATCAGAATAAATA

>JTB_3999993_1

GGATCTCATAGCCTGCATTGGCGTGGTTTGCTC

>JTB_3141429_1

TTTTTGCCAAGCACATTCTCACGAATTTCCAAA

>JTB_4853964_1

CGATCATCCGGACGTCGCAACTATGTTGAACAT

>JTB_2827393_5

CAAATTTGCTTAATGATGCGCTTGCAATTCGTG

>JTB_3357480_1

TGGGAGTGTCCGACGAAAACAGACGTCAAAGGA

>JTB_3500795_1

TCTCTGTATACTAAAGCCAGGATGTTCAACATA

>JTB_3690357_1

GTTCTTCAAGGGCTGGAAGCCTTAAGGGTTGAA

>JTB_357356_2

CCGCCATATTCTCCATACGGTGTGTTTTCACGA

>JTB_4278545_1

GATTTTTCGATTTGTTCTCTTCTCTTTCTTCGG

>JTB_1644761_1

CTTTTCTTCCTCAAGCTGGGCAACCGTCTGCTC

>JTD_2003460_1

TATGTCTCTCCTCGGACTCATCTTCCGGGAAGA

>JTB_3970034_1

GGCCGAAAAGCAAAAGTTACGTACACAGGTGCG

>JTB_128378_3

GTGATTTTCGCCTAAGGTTTTTTCACGAATTGC

>JTB_2592231_1

AGCAACAGGAAAAATCACGCTCGGACCCAGTGG

>JTC_1350685_1

GCGTGGTGGAACTCTTCCCGGAAGATGAGTCCG

>JTB_1575101_1

GACGATTATGTCAGGAAAATGCCTGGCTGCGGG

>JTB_3481024_1

TGAACATCCTGGCTTTAGTATACAGAGATCAGA

>JTC_3339640_1

TGACGATAACCAAGAAAATGAAGAGCAACAGGA

>JTB_4014793_1

GGAGGCTGCAAATTTGCTTAATGATGCGCTTGC

>JTB_3985305_1

GGCAAATACAAGGATGCCGAGCCGCTTTGCAAG

>JTB_3616248_1

GTTTTCACGATTTTTCGATTTGTTCTCTTCTCT

>JTB_4934800_1

CCTGTGCTTCTCCTAAGCCCAATTCGATGTTTT

>JTB_119019_3

TGATAGCTATGACGAAGAGGGAAAGCGATCTCA

>JTB_3783557_1

GTCGTGCTTGCATCTCATCTACAAAATATCGAA

>JTC_294958_2

CTGATTTGGTGGTGTTGGGGACATGGTATGTCT

>JTC_2920229_2

GGAGGCTGCAAATTTGCTTAATGATGCGCTTGC

>JTB_2990355_2

GTACTTTCCTTGTCGTCTGTAGAGGGCGCCTAG

>JTB_839194_1

TAGAGGGCGCCTAGATTCTTCAATGTTGTCGTC

>JTB_1434987_1

GCGAGATGAACTAGCAAACACACAACAGAAGCT

>JTB_1881363_1

CGTGACCGCTGGTTCTCTCCAAATCCTCCAGAG

>JTB_2611826_1

AGACATACCATGTCCCCAACACCACCAAATCAG

>JTD_153300_2

CAAGGATGCCGAGCCGCTTTGCAAGCGAGCTTT

>JTC_2707501_1

AAAAAGAGGCGCTGGATTTAGTAAAGCAGTCCA

>JTB_374939_2

CAACCGTCTGCTCTGACGATTGGAGCTTCTGTT

>JTB_4650467_1

CGTAACTTTTGCTTTTCGGCCTCGATATTTTGT

>JTB_5321326_1

AGCTATGACGAAGAGGGAAAGCGATCTCAAAAG

>JTA_789155_1

GCTCGGCATCCTTGTATTTGCCACGTTTACCAT

>JTB_3158000_1

TTTTCACGATTTTTCGATTTGTTCTCTTCTCTT

>JTB_4989130_1

CCGCTACCTCGTAACGACCTTGAGACGCGTACT

>JTC_2676202_1

AAATCCTGGGAGTGTCCGACGAAAACAGACGTC

>JTC_4739309_1

CGACAACATTGAAGAATCTAGGCGCCCTCTACA

>JTD_2131972_1

GTCCGACGAAAACAGACGTCAAAGGATGAAGGA

>JTB_3088122_2

CGAAAACAGACGTCAAAGGATGAAGGACAGTGA

>JTD_2699642_1

CCTTCATCCTTTGACGTCTGTTTTCGTCGGACC

>JTD_3239351_2

CGCTTTCCCTCTTCGTCATAGCTATCACTGTCC

>JTC_927102_1

GTTCGAGACTATTTCTTCTTGACTCATTTGCGT

>JTC_5177214_1

AGACGTCAAAGGATGAAGGACAGTGATAGCTAT

>JTD_887718_1

CTGTCCTTCATCCTTTGACGTCTGTTTTCGTCG

>JTC_3784383_1

GGGATCTCATAGCCTGCATTGGCGTGGTTTGCA

>JTC_3897331_1

GGACATGGTATGTCTCTCCTCGGACTCATCTTC

>JTC_1960569_1

CGGAGCGATCAACGGTGAAAACAAGCCTATCTG

>JTB_4152113_1

GCGTACTGGATAACCAGATTGTGCAATGTCCTC

>JTB_1033479_1

GTCTGCTCTGACGATTGGAGCTTCTGTTGTGTG

>JTC_2808270_3

TGGAACTCTTCCCGGAAGATGAGTCCGAGGAGA

>JTB_5044903_1

CCACTTTGCAAACAAGCTCTGGAGGATTTGGAG

>JTB_4722328_1

CGGCCTCCTTATATTTATTCTGATCTCTGTATA

>JTD_4306799_1

CATCCTTGTATTTGCCACGTTTACCATAAAGCA

>JTB_5088938_1

CAGGATGTTCAACATAGTTGCGACGTCCGGATG

>JTB_5192976_1

CAAACAAGCTCTGGAGGATTTGGAGAGAACCAG

>JTB_4469758_1

CTTCATTTTCTTGGTTATCGTCATATTTCTTCA

>JTB_2198952_1

CCTGCATTGGCGTGGTTTGCAAACTGATTTGGT

>JTB_2945717_2

TGGTTCTCTCCAAATCCTCCAGAGCTTGTTTGC

>JTB_2802911_6

GTTGGCACAAAGCAGCTAAAGTTGACTCTCCTA

>JTC_2159058_1

CCTCTAAGGTATCAGCAGCCTCGTACTTTCCTT

>JTB_3566436_1

TATCACTGTCCTTCATCCTTTGACGTCTGTTTT

>JTA_1369568_1

CACCTGTGTACGTAACTTTTGCTTTTCGGCCTC

>JTB_2469487_1

ATTTTTGCCAAGCACATTCTCACGAATTTCCAA

>JTC_220141_2

GTTTACCATAAAGCACAGCTAGATTGTTGAGAG

>JTB_4393554_1

GAAAAATCACGCTCGGACCCAGTGGTGGAACTC

>JTB_27978_9

GGATCTCATAGCCTGCATTGGCGTGGTTTGCAA

>JTD_1333852_1

CAAAGCAGCTAAAGTTGACTCTCCTACTGTGAC

>JTB_4121349_1

GCTGGTTCTCTCCAAATCCTCCAGAGCTTGTTT

>JTB_1238628_1

GGCAAATACAAGGATGCCGAGCCGCTTTGCAAG

>JTA_66247_4

CTTCCTTATATTTGCCTTGTTTCAGATAGCAGC

>JTD_5256042_1

CTTCTCTTTCTTCGGCAACCTGCCAGATAGGCT

>JTB_5127858_1

CACGATTTTTCGATTTGTTCTCTTCTCTTTCTT

>JTB_3554628_1

TCAACGGTGAAAACAAGCCTATCTGGCAGGTTG

>JTB_5021598_1

CCCGGAAGATGAGTCCGAGGAGAGACATACCAT

>JTB_2744167_1

AAAAGTGCTAAATTGTTCAGTTGTTTTGCAACG

>JTC_93340_4

GCACAAAGCAGCTAAAGTTGACTCTCCTACTGT

>JTC_4786001_1

CCTCTTCGTCATAGCTATCACTGTCCTTCATCC

>JTC_5040834_1

CAAAAAAGAGGCGCTGGATTTAGTAAAGCAGTC

>JTA_2299409_1

GGTAAACGTGGCAAATACAAGGATGCCGAGCCG

>JTC_989605_1

GTGAAAACAAGCCTATCTGGCAGGTTGCCGAGG

>JTB_2913769_3

CCTCAAGCTGGGCAACCGTCTGCTCTGACGATT

>JTB_2584303_1

AGCGGTCCCACTTTGCAAACAAGCTCTGGAGGA

>JTB_750984_1

TGAAAACAAGCCTATCTGGCAGGTTGCCGAAGA

>JTB_4143557_1

GCTAGTTCATCTCGCAGCCAGGCATTTTCCTGA

>JTB_3430034_1

TGCAATTCGTGAAAAAACCTTAGGCGAAAATCA

>JTB_1177189_1

GGGATCTCATAGCCTGCATTGGCGTGGTTTGCG

>JTB_488453_1

TTGCTCTTCATTTTCTTGGTTATCGTCATATTT

>JTB_3112938_2

CAAAAAAGAGGCGCTGGATTTAGTAAAGCAGTC

>JTB_892880_1

GTTTGCAAACTGATTTGGTGGTGTTGGGGACAT

>JTB_2717040_1

AACAAATCGAAAAATCGTGAAAACACACCGTAT

>JTD_3025281_1

AAGGACAGTGATAGCTATGACGAAGAGGGAAAG

>JTB_4500865_1

CTGGGATCTCATAGCCTGCATTGGCGTGGTTTC

>JTA_2708003_1

CGTTGAGAGTAGCAGCTACAGCTGGGTGATTTT

>JTB_112832_3

TTGGCACAAAGCAGCTAAAGTTGACTCTCCTAC

>JTC_2425939_1

CAAAAAAGAGGCGCTGGATTTAGTAAAGCAGTC

>JTC_1830419_1

CGTGCTTGCATCTCATCTACAAAATATCGAGGC

>JTA_269465_2

CAGACGGTTGCCCAGCTTGAGGAAGAAAAGAAA

>JTC_2349805_1

CACGATCATCCGGACGTCGCAACTATGTTGAAC

>JTB_2868847_3

TGCAAACTGATTTGGTGGTGTTGGGGACATGGT

>JTC_1618642_1

CTTGTATTTGCCACGTTTACCATAAAGCACAGC

>JTD_224128_1

TTCTCTTCTCTTTCTTCGGCAACCTGCCAGATA

>JTA_2391398_1

GGAACTCTTCCCGGAAGATGAGTCCGAGGAGGG

>JTB_5372048_1

AGAAACATCTGGAGTTCATGGCATCCGTGAAGA

>JTB_4601185_1

CTAGCAAACACACAACAGAAGCTCCAATCGTCA

>JTB_5126144_1

CACGGATGCCATGAACTCCAGATGTTTCTTTTC

>JTB_1370160_1

GCTGGGTGATTTTCGCCTAAGGTTTTTTCACGA

>JTB_3257714_1

TTCGAGACTATTTCTTCTTGACTCATTTGCGTC

>JTB_2902098_3

CTCGGACCCAGTGGTGGAACTCTTCCCGGAAGA

>JTC_626309_1

TGGAACTCTTCCCGGAAGATGAGTCCGAGGAGC

>JTC_5004576_1

CAACTGAACAATTTAGCACTTTTGTGTCAGAAC

>JTB_172244_3

CAAGGATGCCGAGCCGCTTTGCAAGCGAGCTTT

>JTC_333384_2

CGAAAACAGACGTCAAAGGATGAAGGACAGTGA

>JTB_2920583_3

CAAACACACAACAGAAGCTCCAATCGTCAGAGC

>JTB_3547687_1

TCATAGCCTGCATTGGCGTGGTTTGCAAACTGA

>JTB_3858326_1

GGTTGCGAAAATCCTGGGAGTGTCCGACGAAAA

>JTB_2729130_1

AAAGCAGCTAAAGTTGACTCTCCTACTGTGACG

>JTB_1493390_1

GCAGGTTCTAACTCGTGCTCACGAGAGGGAATT

>JTB_4274627_1

GCAAACTGATTTGGTGGTGTTGGGGACATGGCA

>JTB_26886_9

TGGAACTCTTCCCGGAAGATGAGTCCGAGGAGA

>JTD_1710608_2

GTTGCCGAAGAAAGAGAAGAGAACAAATCGAAA

>JTB_2934227_2

TTTCACGATTTTTCGATTTGTTCTCTTCTCTTT

>JTC_200425_2

TGCCGAGCCGCTTTGCAAGCGAGCTTTGGAAAT

>JTB_5169272_1

CAACGGTGAAAACAAGCCTATCTGGCAGGTTGC

>JTB_4516839_1

CTGCTGATACCTTAGAGGATTGCGCCCTACGCA

>JTB_5342664_1

AGAGGGCGCCTAGATTCTTCAATGTTGTCGTCA

>JTB_151964_3

CTGACACAAAAGTGCTAAATTGTTCAGTTGTTT

>JTD_3016123_1

AATCCTTGTATTTGCCACGTTTACCATAAAGCA

>JTC_1620343_1

CTTGGTTATCGTCATATTTCTTCACGGATGCCC

>JTB_2818950_5

GGAAAATGCCTGGCTGCGAGATGAACTAGCAAA

>JTB_3902089_1

GGGGGTCCGAGCGTGATTTTTCCTGTTGCTCTT

>JTB_1091039_1

GTAGGGCGCAATCCTCTAAGGTATCAGCAGCCT

>JTD_2468719_1

CTGTCCTTCATCCTTTGACGTCTGTTTTCGTCG

>JTB_670917_1

TGCTAGTTCATCTCGCAGCCAGGCATTTTCCTG

>JTC_5308667_1

AAAATGAAGAGCAACAGGAAAAATCACGCTCGG

>JTB_1759148_1

CTGCTTGTACAGAATTTCAGCTTCCTTATATTT

>JTC_4384437_1

CTGAAATTCTGTACAAGCAGGTTCTAACTCGTG

>JTB_1904539_1

CGGTTCTGACACAAAAGTGCTAAATTGTTCAGT

>JTC_675973_1

TGATCGTGACCGCTGGTTCTCTCCAAATCCTCC

>JTB_233869_2

GTTCGAGACTATTTCTTCTTGACTCATTTGCGT

>JTB_3172171_1

TTTGCTTAATGATGCGCTTGCAATTCGTGAAAA

>JTA_761627_1

GGAACTCTTCCCGGAAGATGAGTCCGAGGAGAG

>JTB_1664046_1

CTTTACTAAATCCAGCGCCTCTTTTTTGCTGCG

>JTB_483568_1

TTGGCACAAAGCAGCTAAAGTTGACTCTCCTCC

>JTB_4375013_1

GAAATATGACGATAACCAAGAAAATGAAGAGCA

>JTA_2552258_1

GAACTCTTCCCGGAAGATGAGTCCGAGGAGATA

>JTB_1635374_1

GAAAAATCGTGAAAACACACCGTATGGAGAATA

>JTB_3504978_1

TCTCCACTTCTTCATACTTTGTCTGGTTCTGAC

>JTB_3114104_2

ATTTGGTGGTGTTGGGGACATGGTATGTCTCTC

>JTC_458942_1

TTGGCACAAAGCAGCTAAAGTTGACTCTCCTAC

>JTC_247981_2

GGATCTCATAGCCTGCATTGGCGTGGTTTGCAA

>JTB_598294_1

TGGTGTTGGGGACATGGTATGTCTCTCCTCGGC

>JTB_5167614_1

CAACTATGTTGAACATCCTGGCTTTAGTATACA

>JTA_1835089_2

CGGTCCCACTTTGCAAACAAGCTCTGGAGGATT

>JTB_4439131_1

CTTGCATCTCATCTACAAAATATCGAGGCCGAA

>JTB_1524947_1

GATTTTTCCTGTTGCTCTTCATTTTCTTGGTTA

>JTC_1752962_1

CTCGTAACGACCTTGAGACGCGTACTGGATAAC

>JTB_2837946_4

GTCGTCTGTAGAGGGCGCCTAGATTCTTCAATG

>JTD_638103_1

GCCATGAACTCCAGATGTTTCTTTTCTTCCTCA

>JTB_5307840_1

AGGATTTGGAGAGAACCAGCGGTCACGATCATC

>JTB_2545998_1

ATACCATGTCCCCAACACCACCAAATCAGTTTG

>JTB_3145691_1

TTTTGTGTCAGAACCAGACAAAGTATGAAGAAG

>JTB_4615920_1

CGTTTACCATAAAGCACAGCTAGATTGTTGAGA

>JTB_375776_2

CAAATTTGCTTAATGATGCGCTTGCAATTCGTG

>JTB_1199613_1

GGCTTAGGAGAAGCACAGGTGATTGTCGTGCTT

>JTB_3317778_1

TGTCGTCATATTTCTTCACGGATGCCATGAACT

>JTB_3582450_1

TACCAAAGAGCCTTGGAAATATACGAAATGAAG

>JTB_3194225_1

TTTCCCTCTTCGTCATAGCTATCACTGTCCTTC

>JTA_2667169_1

CTCGTAACGACCTTGAGACGCGTACTGGATAAC

>JTB_3653265_1

GTTTACCATAAAGCACAGCTAGATTGTTGAGAG

>JTB_2878387_3

GTGATTTTTGCCAAGCACATTCTCACGAATTTC

>JTC_3662953_1

GTCGCAACTATGTTGAACATCCTGGCTTTAGTA

>JTB_2534030_1

ATCCGGACGTCGCAACTATGTTGAACATCCTGG

>JTD_4217063_1

CCTTCATCCTTTGACGTCTGTTTTCGTCGGACA

>JTB_1509229_1

GCAATGTCCTCAAGCGCGCTGGGATCTCATAGC

>JTC_237510_2

GGTTTGCAAACTGATTTGGTGGTGTTGGGGACA

>JTB_4414697_1

CTTTCTTCGGCAACCTGCCAGATAGGCTTGTTT

>JTC_894578_1

GTTGGCACAAAGCAGCTAAAGTTGACTCTCCTA

>JTB_243718_2

GTCCGAGGAGAGACATACCATGTCCCCAACACC

>JTB_5199565_1

CAAAAATCACCCGGACGTTGCAAAACAACTGAA

>JTC_146976_3

CTTCGTCATAGCTATCACTGTCCTTCATCCTTT

>JTB_4041513_1

GGACAGTGATAGCTATGACGAAGAGGGAAAGCG

>JTC_3011486_2

CAAACACACAACAGAAGCTCCAATCGTCAGAGC

>JTC_2271474_1

CCAATTCGATGTTTTCAATATTTTTCATAACAA

>JTB_96925_4

CTCGGACCCAGTGGTGGAACTCTTCCCGGAAGA

>JTB_2418459_1

CAATCCTCTAAGGTATCAGCAGCCTCGTACTTT

>JTD_2778278_1

CATCCTTGTATTTGCCACGTTTACCATAAAGCA

>JTB_183150_2

TTTTCTTCCTCAAGCTGGGCAACCGTCTGCTCT

>JTB_946833_1

GTTGAACACTTAACCCTCATTTCCAACCTTACT

>JTC_5271896_1

AAGAAAGAGAAGAGAACAAATCGAAAAATCGTG

>JTB_2878254_3

GTGGAACTCTTCCCGGAAGATGAGTCCGAGGAG

>JTB_5281192_1

ATAGCCTGCATTGGCGTGGTTTGCAAACTGATT

>JTC_843568_1

GTTTTCACGATTTTTCGATTTGTTCTCTTCTCT

>JTB_4492915_1

CTGGTTCTCTCCAAATCCTCCAGAGCTTGTTTG

>JTC_3219442_1

TGGTTATCCAGTACGCGTCTCAAGGTCGTTACG

>JTC_2610138_1

ACGAAAACAGACGTCAAAGGATGAAGGACAGCG

>JTB_5466029_1

AAATGAAGAGCAACAGGAAAAATCACGCTCGGA

>JTB_4556111_1

CTCGTAACGACCTTGAGACGCGTACTGGATAAC

>JTB_4811137_1

CGCCTCCTTATATTTATTCTGATCTCTGTATAC

>JTB_4594160_1

CTCAACAATCTAGCTGTGCTTTATGGTAAACGT

>JTB_1241124_1

GGATTTAGTAAAGCAGTCCAAGGTTGCGAAAAT

>JTB_1758862_1

CTGCTTTGTGCCAACCGCCATATTCTCCATACG

>JTB_5351104_1

AGACATACCATGTCCCCAACACCACCAAATCAG

>JTB_300064_2

CTTGGTTATCGTCATATTTCTTCACGGATGCCA

>JTB_203135_2

TGGAGTTCATGGCATCCGTGAAGAAATATGACG

>JTD_134037_2

CGGCACCTGTGTACGTAACTTTTGCTTTTCGGC

>JTB_1335132_1

GGAAATTGTTATGAAAAATATTGAAAACATCGA

>JTC_1319900_1

GCTTAATGATGCGCTTGCAATTCGTGAAAAAAC

>JTB_4634164_1

CGTCTGTAGAGGGCGCCTAGATTCTTCAATGTT

>JTB_319254_2

CTAAGGTATCAGCAGCCTCGTACTTTCCTTGTC

>JTB_3579108_1

TACGTACACAGGTGCGACGATTATGTCAGGAAA

>JTB_5160518_1

CAAGCACGACAATCACCTGTGCTTCTCCTAAGC

>JTB_2884895_3

GGATCTCATAGCCTGCATTGGCGTGGTTTGCCA

>JTC_435588_1

TTTCCAAGGCTCTTTGGTAATACATCTCCACTT

>JTC_3108862_1

TTGGCGTGGTTTGCAAACTGATTTGGTGGTGTT

>JTB_55957_6

CACAAAGCAGCTAAAGTTGACTCTCCTACTGTG

>JTB_3393857_1

TGGAAATGAGGGTTAAGTGTTCAACCCTTAAGG

>JTB_2039253_1

CGCTTTCCCTCTTCGTCATAGCTATCACTGTAC

>JTB_1955210_1

CGGGAGTGTCCGACGAAAACAGACGTCAAAGGA

>JTB_2797665_7

CTCATAGCCTGCATTGGCGTGGTTTGCAAACTG

>JTC_993639_1

GTCTGTAGAGGGCGCCTAGATTCTTCAATGTAG

>JTB_769786_1

TCTCAAGGTCGTTACGAGGTAGCGGTCCCACTT

>JTB_5043601_1

CCAGAGCTTGTTTGCAAAGTGGGACCGCTACCT

>JTB_1746503_1

CTGGCTGCGAGATGAACTAGCAAACACACAACA

>JTA_2129129_1

TAGCGGTCCCACTTTGCAAACAAGCTCTGGAGG

>JTB_3923185_1

GGGATCTCATAGCCTGCATTGGCGTGGTTTGCA

>JTB_1128772_1

GGTCTTTTTTGCTGCCTTCAGTAAGGTTGGAAA

>JTD_74875_2

TGTCCTTCATCCTTTGACGTCTGTTTTCGTCGG

>JTC_3953380_1

GGAAAATGCCTGGCTGCGAGATGAACTAGCACA

>JTB_3682750_1

GTTGAGAGTAGCAGCTACAGCTGGGTGATTTTT

>JTB_4543872_1

CTCTTCCCGGAAGATGAGTCCGAGGAGAGACAT

>JTB_2929951_2

TTTTCTTCCTCAAGCTGGGCAACCGTCTGCTCT

>JTB_188774_2

TTGTCGTGCTTGCATCTCATCTACAAAATATCG

>JTB_5426385_1

AATCACCCAGCTGTAGCTGCTACTCTCAACAAT

>JTB_198079_2

TGTCGTCACAGTAGGAGAGTCAACTTTAGCTGC

>JTB_2184563_1

CGAAAAATCGTGAAAACACACCGTATGGAGAAT

>JTB_2952583_2

TGCCGAGCCGCTTTGCAAGCGAGCTTTGGAAAT

>JTB_3810292_1

GTCCGACGAAAACAGACGTCAAAGGATGAAGGC

>JTD_1172873_1

CCTCCTTATATTTATTCTGATCTCTGTATACTA

>JTC_2951445_2

CTTGTCGTCTGTAGAGGGCGCCTAGATTCTTCA

>JTB_223732_2

GTTTTGCAACGTCCGGGTGATTTTTGCCAAGCA

>JTD_2192358_1

GGCATCCTTGTATTTGCCACGTTTACCATAAAG

>JTD_4566531_1

AATCTAGGCGCCCTCTACAGACGACAAGGAAAG

>JTB_123526_3

GTTTTCACGATTTTTCGATTTGTTCTCTTCTCT

>JTB_1155801_1

GGGGGTCCGAGCGTGATTTTTCCTGTTGCTCTT

>JTB_2820272_5

GCAAACTGATTTGGTGGTGTTGGGGACATGGTA

>JTB_5488121_1

AAAATATCGAGGCCGAAAAGCAAAAGTTACGTA

>JTB_1789826_1

CTCTTCCTCAAGCTGGGCAACCGTCTGCTCTGA

>JTB_2899282_3

CTTCCCGGAAGATGAGTCCGAGGAGAGACATAC

>JTB_2838325_4

GTCCGACGAAAACAGACGTCAAAGGATGAAGGA

>JTA_1006773_1

CTGCGTAGGGCGCAATCCTCTAAGGTATCAGCA

>JTB_213917_2

TCTTCCCGGAAGATGAGTCCGAGGAGAGACATA

>JTB_3130087_2

ACAAATCGAAAAATCGTGAAAACACACCGTATG

>JTB_370400_2

CACGATCATCCGGACGTCGCAACTATGTTGAAC

>JTB_4272992_1

GCAAATATAAGGAAGCTGAAATTCTGTACAAGC

>JTC_3440134_1

TATAAGGAAGCTGAAATTCTGTACAAGCAGGTT

>JTD_1958516_1

TGATCGTGACCGCTGGTTCTCTCCAAATCCTCC

>JTB_3594638_1

TAAAGCCAGGATGTTCAACATAGTTGCGACGTC

>JTB_1774253_1

CTGATGATCCAAACGTCGCCAAGACCAAGAACA

>JTB_3046759_2

CTTCATCCTTTGACGTCTGTTTTCGTCGGACAC

>JTB_3378942_1

TGGAGGCTGCAAATTTGCTTAATGATGCGCTTG

>JTC_4884940_1

CCACTTTGCAAACAAGCTCTGGAGGATTTGGAG

>JTC_4314844_1

CTTCATTTTCTTGGTTATCGTCATATTTCTTCA

>JTB_2724672_1

AAATAGTCTCGAACACTAAAACGGTTCTTCAAG

>JTB_3535032_1

TCCGGGTGATTTTTGCCAAGCACATTCTCACGT

>JTC_1569341_1

GAAAGAGAAGAGAACAAATCGAAAAATCGTGAA

>JTB_1682043_1

CTTGCAAGATTGTTCTTGGTCTTGGCGACGTTT

>JTC_1846326_1

CGTCACAGTAGGAGAGTCAACTTTAGCTGCTTT

>JTD_384887_1

GTTTGCTAGTTCATCTCGCAGCCAGGCATTTTT

>JTD_3948507_1

CTGTCCTTCATCCTTTGACGTCTGTTTTCGTCG

>JTB_3030080_2

GATCAACGGTGAAAACAAGCCTATCTGGCAGGT

>JTB_1566083_1

GAGAAGCACAGGTGATTGTCGTGCTTGCATCTC

>JTD_308524_1

TGAAGGACAGTGATAGCTATGACGAAGAGGGCA

>JTC_816635_1

TAAGTCGGAAATTGTTATGAAAAATATTGAAAA

>JTC_2902524_2

GTGCAATGTCCTCAAGCGCGCTGGGATCTCATA

>JTB_113536_3

TTCTGACACAAAAGTGCTAAATTGTTCAGTTGT

>JTA_2068513_1

TGACACAAAAGTGCTAAATTGTTCAGTTGTTTT

>JTC_3850272_1

GGATGTTCAACATAGTTGCGACGTCCGGATGAT

>JTC_5052899_1

ATTTGCTTAATGATGCGCTTGCAATTCGTGAAA

>JTB_436601_1

TTTGCTTAATGATGCGCTTGCAATTCGTGAAAA

>JTB_3304360_1

TGTGTTTTCACGATTTTTCGATTTGTTCTCTTC

>JTA_2670016_1

CTCGGCATCCTTGTATTTGCCACGTTTACCATA

>JTC_3159360_1

TTCAGATAGCAGCTTGCAAGATTGTTCTTGGTC

>JTC_1208864_1

GGATCTCATAGCCTGCATTGGCGTGGTTTGCTA

>JTB_804154_1

TCCCTCTTCGTCATAGCTATCACTGTCCTTCAT

>JTB_3317546_1

TGTCGTGCTTGCATCTCATCTACAAAATATCGA

>JTC_4077971_1

GCCATATTCTCCATACGGTGTGTTTTCACGATT

>JTB_2013966_1

CGGACGTTGCAAAACAACTGAACAATTTAGCCC

>JTD_2132459_1

GTCCCACTTTGCAAACAAGCTCTGGAGGATTTG

>JTB_3045194_2

CTTCGTCATAGCTATCACTGTCCTTCATCCTTT

>JTB_2516176_1

ATGCCTGGCTGCGAGATGAACTAGCAAACACAC

>JTB_4793846_1

CGCTACCTCGTAACGACCTTGAGACGCGTACTG

>JTB_812420_1

TCATAGCCTGCATTGGCGTGGTTTGCAAACTGT

>JTB_1985877_1

CGGCAGCAAAAAAGAGGCGCTGGATTTAGTAAA

>JTB_183770_2

TTTTAGTGTTCGAGACTATTTCTTCTTGACTCA

>JTB_2642317_1

ACTAAATCCAGCGCCTCTTTTTTGCTGCGTAGG

>JTD_3345570_1

TTCATCCTTTGACGTCTGTTTTCGTCGGACAGT

>JTC_4786290_1

CCTCTGTATACTAAAGCCAGGATGTTCAACATC

>JTB_2266617_1

CCGAGCGTGATTTTTCCTGTTGCTCTTCATTTT

>JTD_4891602_1

TAGCAAACACACAACAGAAGCTCCAATCGTCAG

>JTD_4491710_1

AGGATGTTCAACATAGTTGCGACGTCCGGATGA

>JTB_1526370_1

GATTTGGTGGTGTTGGGGACATGGTATGTCTCT

>JTB_2718283_1

AAATTTGCAGCCTCCTTATATTTATTCTGATCT

>JTC_1576649_1

GAAAATGCCTGGCTGCGAGATGAACTAGCAAAC

>JTB_2610471_1

AGACGACAAGGAAAGTACGAGGCTGCTGATACC

>JTD_2420394_1

CTTTAGTATACAGAGATCAGAATAAATATAAGG

>JTB_1799630_1

CTCGTGCTCACGAGAGGGAATTCGGAGCGATCA

>JTB_3051729_2

CTGCAAATTTGCTTAATGATGCGCTTGCAATTC

>JTC_5022637_1

CAAATACAAGGATGCCGAGCCGCTTTGCAAGCG

>JTB_62786_5

GTCCGACGAAAACAGACGTCAAAGGATGAAGGA

>JTB_1845420_1

CTATGACGAAGAGGGAAAGCGATCTCAAAAGGA

>JTC_3093920_1

TTTAGTGTTCGAGACTATTTCTTCTTGACTCAT

>JTB_5391567_1

ACGATCATCCGGACGTCGCAACTATGTTGAACA

>JTB_142114_3

GCCATATTCTCCATACGGTGTGTTTTCACGATT

>JTB_3054621_2

CTCGTACTTTCCTTGTCGTCTGTAGAGGGCGCC

>JTA_67480_4

CTCGTAACGACCTTGAGACGCGTACTGGATAAC

>JTC_1583273_1

GAAAAATATTGAAAACATCGAATTGGGCTTAGG

>JTB_2763127_31

GGAACTCTTCCCGGAAGATGAGTCCGAGGAGAG

>JTB_5235021_1

ATTCGGAGCGATCAACGGTGAAAACAAGCCTAT

>JTB_1496130_1

GCAGCTACAGCTGGGTGATTTTCGCCTAAGGTT

>JTC_4475505_1

CGTGCTTGCATCTCATCTACAAAATATCGAGGC

>JTC_766255_1

TCCGGACGTCGCAACTATGTTGAACATCCTGGC

>JTC_4030189_1

GCGCCCTACGCAGCAAAAAAGAGGCGCTGGATT

>JTB_5132609_1

CACCGCTGGTTCTCTCCAAATCCTCCAGAGCTT

>JTB_3020099_2

GCGAGATGAACTAGCAAACACACAACAGAAGCT

>JTC_1471189_1

GCAAACCACGCCAATGCAGGCTATGAGATCCCA

>JTB_3173798_1

TTTGCCTTGTTTCAGATAGCAGCTTGCAAGATT

>JTD_1888891_1

TTCCTTGTCGTCTGTAGAGGGCGCCTAGATTCT

>JTB_4924213_1

CGAAAATCCTGGGAGTGTCCGACGAAAACAGAC

>JTB_3040443_2

CTTTGGTAATACATCTCCACTTCTTCATACTTT

>JTB_1031760_1

GTCTGTTTTCGTCGGACACTCCCAGGATTTTCG

>JTB_3575071_1

TAGAGGATTGCGCCCTACGCAGCAAAAAAGAGG

>JTC_456604_1

TTGGGATCTCATAGCCTGCATTGGCGTGGTTTG

>JTA_128653_3

CACAAAGCAGCTAAAGTTGACTCTCCTACTGTG

>JTB_361837_2

CCAGCGGTCACGATCATCCGGACGTCGCAACTA

>JTB_2756549_1

AAAAAAGAGGCGCTGGATTTAGTAAAGCAGTCC

>JTD_958213_1

CTCCACTTCTTCATACTTTGTCTGGTTCTGACA

>JTC_1611639_1

CTTTAGTATACAGAGATCAGAATAAATATAAGG

>JTB_2837100_4

GTTCAACATAGTTGCGACGTCCGGATGATCGTG

>JTC_4236926_1

GAAAAATATTGAAAACATCGAATTGGGCTTAGG

>JTC_281418_2

GAAAAATCGTGAAAACACACCGTATGGAGAATA

>JTB_1669592_1

CTTGTATTTGCCACGTTTACCATAAAGCACAGC

>JTB_3152769_1

TTTTCTTGGTTATCGTCATATTTCTTCACGGAT

>JTB_437108_1

TTTGCTAGTTCATCTCGCAGCCAGGCATTTTCC

>JTB_1728477_1

CTGTTGTGTGTTTGCTAGTTCATCTCGCAGCCA

>JTB_5233905_1

ATTCGTCATAGCTATCACTGTCCTTCATCCTTT

>JTD_2704184_1

CCTGCATTGGCGTGGTTTGCAAACTGATTTGGT

>JTB_2470005_1

ATTTTTCCTGTTGCTCTTCATTTTCTTGGTTAT

>JTC_1480461_1

GATTCTTCAATGTTGTCGTCACAGTAGGAGAGT

>JTB_5130843_1

CACCTGTGTACGTAACTTTTGCTTTTCGGCCTC

>JTB_2201833_1

CCTCTCGTGAGCACGAGTTAGAACCTGCTTGTA

>JTC_5313598_1

AAAAGAGGCGCTGGATTTAGTAAAGCAGTCCAA

>JTB_3739010_1

GTGGTGTTGGGGACATGGTATGTCTCTCCTCGG

>JTB_5189212_1

CAAACTGATTTGGTGGTGTTGGGGACATGGTAT

>JTB_151572_3

CTGCATTGGCGTGGTTTGCAAACTGATTTGGTG

>JTB_241363_2

GTCGTCTGTAGAGGGCGCCTAGATTCTTCAATG

>JTB_3999994_1

GGATCTCATAGCCTGCATTGGCGTGGTTTGCAT

>JTC_1581939_1

GAAAACAGACGTCAAAGGATGAAGGACAGTGAT

>JTB_1008771_1

GTGGAACTCTTCCCGGAAGATGAGTCCGAGGCG

>JTB_360011_2

CCCGGACGTTGCAAAACAACTGAACAATTTAGC

>JTD_3453823_1

TATCAGCAGCCTCGTACTTTCCTTGTCGTCTGT

>JTB_3076630_2

CGCCTGCATTGGCGTGGTTTGCAAACTGATTTG

>JTB_1753382_1

CTGGAGGATTTGGAGAGAACCAGCGGTCACGAT

>JTB_4449041_1

CTTCTTCATACTTTGTCTGGTTCTGACACAAAA

>JTB_5400925_1

ACCATGTCCCCAACACCACCAAATCAGTTTGCA

>JTA_2256088_1

GTCCCACTTTGCAAACAAGCTCTGGAGGATTTG

>JTB_238970_2

GTGATAGCTATGACGAAGAGGGAAAGCGATCTC

>JTB_4479723_1

CTTATATTTGCCTTGTTTCAGATAGCAGCTTGC

>JTB_5232533_1

ATTCTGATCTCTGTATACTAAAGCCAGGATGTT

>JTB_130452_3

GTCACGATCATCCGGACGTCGCAACTATGTTGA

>JTB_1522190_1

GCAAACAAGCTCTGGAGGATTTGGAGAGAACCA

>JTB_5261915_1

ATCTCATAGCCTGCATTGGCGTGGTTTGCAAAC

>JTB_3013965_2

GCTTGAGGAAGAAAAGAAACATCTGGAGTTCAT

>JTB_4198638_1

GCGAAAATCACCCAGCTGTAGCTGCTACTCTCC

>JTC_148576_3

CTGGGATCTCATAGCCTGCATTGGCGTGGTTTG

>JTD_2726732_1

CCGGAAGATGAGTCCGAGGAGAGACATACCATG

>JTD_3323244_1

TTGTAGATGAGATGCAAGCACGACAATCACCTG

>JTC_3434437_1

TATCTGGCAGGTTGCCGAAGAAAGAGAAGAGAA

>JTB_4356486_1

GAAGATGAGTCCGAGGAGAGACATACCATGTCC

>JTB_2830554_4

TTGGCACAAAGCAGCTAAAGTTGACTCTCCTAC

>JTC_2558377_1

AGAGGATTGCGCCCTACGCAGCAAAAAAGAGGC

>JTB_2722670_1

AAATCGTGAAAACACACCGTATGGAGAATATGG

>JTB_3002803_2

GGATCTCATAGCCTGCATTGGCGTGGTTTGCAC

>JTB_3697509_1

GTTCGTCATAGCTATCACTGTCCTTCATCCTTT

>JTB_5227216_1

ATTGGCGTGGTTTGCAAACTGATTTGGTGGTGT

>JTD_3211272_2

GATAGCTATGACGAAGAGGGAAAGCGATCTCAA

>JTB_3893017_1

GGGTGGGACCGCTACCTCGTAACGACCTTGAGA

>JTC_5166365_1

AGATGAGATGCAAGCACGACAATCACCTGTGCT

>JTC_3973107_1

GCTTAATGATGCGCTTGCAATTCGTGAAAAAAC

>JTB_4038910_1

GGACCCAGTGGTGGAACTCTTCCCGGAAGATGA

>JTB_2813467_5

TGGAACTCTTCCCGGAAGATGAGTCCGAGGAGA

>JTC_1446783_1

GCAGCCTCGTACTTTCCTTGTCGTCTGTAGAGG

>JTB_2246945_1

CCGGAAGATGAGTCCGAGGAGAGACATACCATG

>JTB_1859625_1

CTAAAGTTGACTCTCCTACTGTGACGACAACAT

>JTB_5248128_1

ATGTCCTCAAGCGCGCTGGGATCTCATAGCCTG

>JTD_4316319_1

CAGGTTGCCGAAGAAAGAGAAGAGAACAAATCG

>JTC_4726532_1

CGACGATTATGTCAGGAAAATGCCTGGCTGCGA

>JTB_2589744_1

AGCAGCTAAAGTTGACTCTCCTACTGTGACGAC

>JTB_4842571_1

CGATTATGTCAGGAAAATGCCTGGCTGCGAGAT

>JTA_278313_2

ATTTGGTGGTGTTGGGGACATGGTATGTCTCTC

>JTC_2100625_1

CGACGAAAACAGACGTCAAAGGATGAAGGACAG

>JTB_1149528_1

GGGTCGTCTGTAGAGGGCGCCTAGATTCTTCAA

>JTC_3888631_1

GGACGTTGCAAAACAACTGAACAATTTAGCACT

>JTB_94365_4

CTTTGGTAATACATCTCCACTTCTTCATACTTT

>JTC_4312755_1

CTTCCCGGAAGATGAGTCCGAGGAGAGACATAC

>JTB_618015_1

TGGGGACATGGTATGTCTCTCCTCGGACTCATC

>JTB_2489702_1

ATTGTTCTTGGTCTTGGCGACGTTTGGATCATC

>JTC_413731_1

TTTGCTTTTCGGCCTCGATATTTTGTAGATGAG

>JTB_1602767_1

GAAGAATCTAGGCGCCCTCTACAGACGACAAGG

>JTB_2936810_2

TTGCAAACTGATTTGGTGGTGTTGGGGACATGG

>JTC_2904700_2

GTCGTGCTTGCATCTCATCTACAAAATATCGAG

>JTB_2779101_11

CGGAAGATGAGTCCGAGGAGAGACATACCATGT

>JTB_304477_2

CTTCCCGGAAGATGAGTCCGAGGAGAGACATAC

>JTB_33161_8

GGAAGATGAGTCCGAGGAGAGACATACCATGTC

>JTB_1091264_1

GTAGGAGAGTCAACTTTAGCTGCTTTGTGCCAA

>JTB_4570878_1

CTCCTTATATTTATTCTGATCTCTGTATACTAA

>JTB_10398_18

GGAACTCTTCCCGGAAGATGAGTCCGAGGAGAG

>JTC_664937_1

TGCAAACTGATTTGGTGGTGTTGGGGACATGGT

>JTB_439963_1

TTTGCAAACTGATTTGGTGGTGTTGGGGACATG

>JTA_1233441_1

CGAAAACAGACGTCAAAGGATGAAGGACAGTGC

>JTC_4967107_1

CACGAATTTCCAAAGCTCGCTTGCAAAGCGGCT

>JTC_581711_1

TGGTAAACGTGGCAAATACAAGGATGCCGAGCC

>JTB_534399_1

TTCCAACCTTACTGAAGGCAGCAAAAAAGACCC

>JTB_2970813_2

GTTTGCAAACTGATTTGGTGGTGTTGGGGACAT

>JTB_34406_8

CTGATTTGGTGGTGTTGGGGACATGGTATGTCT

>JTC_4979635_1

CACAATCTGGTTATCCAGTACGCGTCTCAAGGT

>JTB_2426625_1

CAAGCGCGCTGGGATCTCATAGCCTGCATTGGC

>JTB_2952237_2

TGCGACGTCCGGATGATCGTGACCGCTGGTTCT

>JTB_3130122_2

ACAAAGCAGCTAAAGTTGACTCTCCTACTGTGA

>JTC_872535_1

GTTTCAGATAGCAGCTTGCAAGATTGTTCTTGG

>JTB_1540559_1

GATCTCATAGCCTGCATTGGCGTGGTTTGCAAA

>JTB_3078496_2

CGCAATCCTCTAAGGTATCAGCAGCCTCGTACT

>JTB_1830848_1

CTCCACTTCTTCATACTTTGTCTGGTTCTGACA

>JTA_1068424_1

CGTTGAGAGTAGCAGCTACAGCTGGGTGATTTT

>JTB_4894971_1

CGACAATCACCTGTGCTTCTCCTAAGCCCAATT

>JTB_149973_3

CTTCAATGTTGTCGTCACAGTAGGAGAGTCAAC

>JTC_230581_2

GTGAAAACAAGCCTATCTGGCAGGTTGCCGAAG

>JTC_5155573_1

AGCCTCCTTATATTTATTCTGATCTCTGTATAC

>JTB_2060573_1

CGCGCTGGGATCTCATAGCCTGCATTGGCGTGG

>JTC_4031583_1

GCGCAATCCTCTAAGGTATCAGCAGCCTCGTAC

>JTB_482024_1

TTGGCGTGGTTTGCAAACTGATTTGGTGGTGTT

>JTC_810990_1

TACCAAAGAGCCTTGGAAATATACGAAATGAAT

>JTB_591523_1

TGTAGATGAGATGCAAGCACGACAATCACCTGT

>JTB_2183311_1

CGAAAAGCAAAAGTTACGTACACAGGTGCGACG

>JTB_235110_2

GTTCAGTTGTTTTGCAACGTCCGGGTGATTTTT

>JTB_2705675_1

AAGAAAATGAAGAGCAACAGGAAAAATCACGCT

>JTB_1049963_1

GTCGGACACTCCCAGGATTTTCGCAACCTTGGA

>JTC_511561_1

TTCAATGTTGTCGTCACAGTAGGAGAGTCAACT

>JTC_2871286_2

TGTCCGACGAAAACAGACGTCAAAGGATGAAGG

>JTD_1673700_3

CAAGGATGCCGAGCCGCTTTGCAAGCGAGCTTT

>JTB_102874_4

CGAAAACAGACGTCAAAGGATGAAGGACAGTGA

>JTB_3164839_1

TTTGTGCCAACCGCCATATTCTCCATACGGTGT

>JTB_389451_2

AGATGAACTAGCAAACACACAACAGAAGCTCCA

>JTB_2723470_1

AAATCCAGCGCCTCTTTTTTGCTGCGTAGGGCG

>JTB_2893196_3

GCAAATTTGCTTAATGATGCGCTTGCAATTCGT

>JTB_2886437_3

GGAATTCGGAGCGATCAACGGTGAAAACAAGCC

>JTD_4781390_1

TTGTAGATGAGATGCAAGCACGACAATCACCTG

>JTC_5088652_1

ATGCCGAGCCGCTTTGCAAGCGAGCTTTGGAAA

>JTD_758583_1

GATAGCTATGACGAAGAGGGAAAGCGATCTCAA

>JTB_974361_1

GTTCATCTCGCAGCCAGGCATTTTCCTGACATA

>JTB_1469840_1

GCCCTCTACAGACGACAAGGAAAGTACGAGGCT

>JTC_3898326_1

GGACAGTGATAGCTATGACGAAGAGGGAAAGCG

>JTC_989601_1

GTGAAAACACACCGTATGGAGAATATGGCGGGG

>JTB_270375_2

GCTTGGCAAAAATCACCCGGACGTTGCAAAACA

>JTC_2610137_1

ACGAAAACAGACGTCAAAGGATGAAGGACAGTG

>JTB_3049484_2

CTGGGATCTCATAGCCTGCATTGGCGTGGTTTG

>JTB_170295_3

CAGCCTCGTACTTTCCTTGTCGTCTGTAGAGGG

>JTB_3064676_2

CGGTCACGATCATCCGGACGTCGCAACTATGTT

>JTB_69342_5

CTCATAGCCTGCATTGGCGTGGTTTGCAAACTG

>JTB_1341748_1

GGAAAGCAAAAGTTACGTACACAGGTGCGACGA

>JTB_1391263_1

GCTAGATTGTTGAGAGTAGCAGCTACAGCTGGG

>JTC_4442608_1

CTATGACGAAGAGGGAAAGCGATCTCAAAAGTA

>JTC_2954157_2

CTTCGTCATAGCTATCACTGTCCTTCATCCTTT

>JTD_4577321_1

AAGGACAGTGATAGCTATGACGAAGAGGGAACG

>JTB_835106_1

TAGTAAAGCAGTCCAAGGTTGCGAAAATCCTGG

>JTB_597378_1

TGGTTCTGACACAAAAGTGCTAAATTGTTCAGT

>JTB_66343_5

GCACAAAGCAGCTAAAGTTGACTCTCCTACTGT

>JTB_2467669_1

CAAAAAAGACCCAGATAAGTCGGAAATTGTTAT

>JTB_379545_2

ATTTGGAGAGAACCAGCGGTCACGATCATCCGG

>JTB_4313007_1

GAGCCGCTTTGCAAGCGAGCTTTGGAAATTCGT

>JTC_1218202_1

GGAGTGTCCGACGAAAACAGACGTCAAAGGATG

>JTB_3512012_1

TCGTAACGACCTTGAGACGCGTACTGGATAACC

>JTD_2414879_1

CTTTCTTCGGCAACCTGCCAGATAGGCTTGTTT

>JTB_2182465_1

CGAAAATCCTGGGAGTGTCCGACGAAAACAGAC

>JTA_212391_2

GAACTCTTCCCGGAAGATGAGTCCGAGGAGAGA

>JTC_210641_2

TCATAGCCTGCATTGGCGTGGTTTGCAAACTGA

>JTC_2320028_1

CAGCCAGGCATTTTCCTGACATAATCGTCGCAC

>JTB_5456235_1

AACCAGCGGTCACGATCATCCGGACGTCGCAAC

>JTC_3260812_1

TGGAGGCTGCAAATTTGCTTAATGATGCGCTTG

>JTB_812422_1

TCATAGCCTGCATTGGCGTGGTTTGCAAACTGA

>JTB_2116114_1

CGATAACCAAGAAAATGAAGAGCAACAGGAAAA

>JTD_876114_1

CTTATATTTGCCTTGTTTCAGATAGCAGCTTGC

>JTB_1525576_1

GATTTTCGCAACCTTGGACTGCTTTACTAAATC

>JTB_3054123_2

CTCTAAGGTATCAGCAGCCTCGTACTTTCCTTG

>JTC_233841_2

GTCCGACGAAAACAGACGTCAAAGGATGAAGGA

>JTB_5302435_1

AGGTAGCGGTCCCACTTTGCAAACAAGCTCTGG

>JTB_3211334_1

TTGTCGTGCTTGCATCTCATCTACAAAATATCG

>JTD_5452602_1

CGACGTTTGGATCATCAGGCCCAAGCTTCATTT

>JTB_4104869_1

GCTTTGCAAGCGAGCTTTGGAAATTCGTGAGAA

>JTB_236409_2

GTTACGTACACAGGTGCGACGATTATGTCAGGA

>JTD_2219850_1

GGAAGCTGAAATTCTGTACAAGCAGGTTCTAAC

>JTD_786926_1

GAAGAGAACAAATCGAAAAATCGTGAAAACACA

>JTB_3738276_1

GTGGTTTGCAAACTGATTTGGTGGTGTTGGGGA

>JTD_3220573_2

CTTCGTCATAGCTATCACTGTCCTTCATCCTTT

>JTB_1796883_1

CTCTAAGGTATCAGCAGCCTCGTACTTTCCTTG

>JTB_2404549_1

CACATTCTCACGAATTTCCAAAGCTCGCTTGCA

>JTD_4878164_1

TCATAGCTATCACTGTCCTTCATCCTTTGACGT

>JTD_4608141_1

AAAGCAGCTAAAGTTGACTCTCCTACTGTGACG

>JTB_1701164_1

CTTCGGCAACCTGCCAGATAGGCTTGTTTTCCC

>JTD_2526807_1

CTCCACTTCTTCATACTTTGTCTGGTTCTGACA

>JTB_429251_1

TTTGTGCCAACCGCCATATTCTCCATACGGTGT

>JTD_211805_1

TTGTAGATGAGATGCAAGCACGACAATCACCTG

>JTC_4409122_1

CTCGCAGCCAGGCATTTTCCTGACATAATCGTC

>JTC_2908352_2

GTATCAGCAGCCTCGTACTTTCCTTGTCGTCTG

>JTB_2342024_1

CATAGTTGCGACGTCCGGATGATCGTGACCGCT

>JTC_2624455_1

ACAAAGCAGCTAAAGTTGACTCTCCTACTGTGA

>JTB_173221_3

CAAATACAAGGATGCCGAGCCGCTTTGCAAGCG

>JTB_1260446_1

GGATAACCAGATTGTGCAATGTCCTCAAGCGCG

>JTC_2933531_2

GCGAGATGAACTAGCAAACACACAACAGAAGCT

>JTC_936934_1

GTTCATCTCGCAGCCAGGCATTTTCCTGACATA

>JTD_145335_2

CCGAAGAAAGAGAAGAGAACAAATCGAAAAATC

>JTD_2548763_1

CTAAAGTTGACTCTCCTACTGTGACGACAACAT

>JTB_98511_4

CGGTCACGATCATCCGGACGTCGCAACTATGTT

>JTB_3122336_2

AGGAAGATGAGTCCGAGGAGAGACATACCATGT

>JTB_4024197_1

GGAGAGTCAACTTTAGCTGCTTTGTGCCAACCG

>JTB_4397737_1

CTTTTTTGCTGCCTTCAGTAAGGTTGGAAATGA

>JTB_2141796_1

CGACGATTATGTCAGGAAAATGCCTGGCTGCGG

>JTB_709315_1

TGATCGCTCCGAATTCCCTCTCGTGAGCACGAG

>JTD_3237031_2

CGGCATCCTTGTATTTGCCACGTTTACCATAAA

>JTB_1609981_1

GAACCAGCGGTCACGATCATCCGGACGTCGCAA

>JTC_1674040_1

CTTAGGAGAAGCACAGGTGATTGTCGTGCTTGC

>JTC_4701655_1

CGATAACCAAGAAAATGAAGAGCAACAGGAAAA

>JTB_3424038_1

TGCCAACCGCCATATTCTCCATACGGTGTGTTT

>JTB_4134794_1

GCTCTTCATTTTCTTGGTTATCGTCATATTTCT

>JTB_2346143_1

CAGTGGTGGAACTCTTCCCGGAAGATGAGTCCG

>JTB_3233816_1

TTGACGTCTGTTTTCGTCGGACACTCCCAGGAT

>JTC_3984178_1

GCTGCATTGGCGTGGTTTGCAAACTGATTTGGT

>JTB_1710710_1

CTTCCCGGAAGATGAGTCCGAGGAGAGACATCC

>JTC_3547205_1

GTTGCCCAGCTTGAGGAAGAAAAGAAACATCTG

>JTC_591707_1

TGGGATCTCATAGCCTGCATTGGCGTGGTTTGC

>JTB_698589_1

TGATTTGGTGGTGTTGGGGACATGGTATGTCTC

>JTA_1686571_3

GGAACTCTTCCCGGAAGATGAGTCCGAGGAGAG

>JTB_3245497_1

TTCTCCTAAGCCCAATTCGATGTTTTCAATATT

>JTB_4545907_1

CTCTGGAGGATTTGGAGAGAACCAGCGGTCACG

>JTB_5137232_1

CACATTCTCACGAATTTCCAAAGCTCGCTTGCA

>JTC_2350907_1

CACGAATTGCAAGCGCATCATTAAGCAAATTTG

>JTB_285137_2

GCAAACTGATTTGGTGGTGTTGGGGACATGGTA

>JTB_4886880_1

CGACCTTGAGACGCGTACTGGATAACCAGATTG

>JTB_3554310_1

TCAACTTTAGCTGCTTTGTGCCAACCGCCATAT

>JTB_4543275_1

CTCTTCTCTTTCTTCGGCAACCTGCCAGATAGG

>JTB_5448879_1

AAGAAAAGAAACATCTGGAGTTCATGGCATCCG

>JTC_699180_1

TGACACAAAAGTGCTAAATTGTTCAGTTGTTTT

>JTB_2093756_1

CGCAAATGAGTCAAGAAGAAATAGTCTCGAACA

>JTB_445327_1

TTTCTTCCTCAAGCTGGGCAACCGTCTGCTCTG

>JTB_1749784_1

CTGGCAGGTTGCCGAAGAAAGAGAAGAGAACAA

>JTB_3353872_1

TGGGGACATGGTATGTCTCTCCTCGGACTCATC

>JTC_1313066_1

GCTTGCATCTCATCTACAAAATATCGAGGCCCA

>JTC_2394978_1

CAACCGTCTGCTCTGACGATTGGAGCTTCTGTT

>JTB_2309923_1

CCACTTTGCAAACAAGCTCTGGAGGATTTGGAG

>JTC_2552671_1

AGATGAACTAGCAAACACACAACAGAAGCTCCA

>JTB_1008772_1

GTGGAACTCTTCCCGGAAGATGAGTCCGAGGAG

>JTD_5476124_1

CCTTGTATTTGCCACGTTTACCATAAAGCACCG

>JTB_4383774_1

GAAACATCTGGAGTTCATGGCATCCGTGAAGAA

>JTB_4548938_1

CTCTCCAAATCCTCCAGAGCTTGTTTGCAAAGT

>JTB_5040980_1

CCAGCTTGAGGAAGAAAAGAAACATCTGGAGTT

>JTB_1907117_1

CGGTGTGTTTTCACGATTTTTCGATTTGTTCTC

>JTC_3012885_2

ATTTTCTTGGTTATCGTCATATTTCTTCACGGA

>JTB_965358_1

GTTCCTTGTCGTCTGTAGAGGGCGCCTAGATTC

>JTC_4118278_1

GCAACCGTCTGCTCTGACGATTGGAGCTTCTGT

>JTB_2089759_1

CGCACCTGTGTACGTAACTTTTGCTTTTCGGCC

>JTB_507684_1

TTCTGATCTCTGTATACTAAAGCCAGGATGTTC

>JTC_1458403_1

GCAATGTCCTCAAGCGCGCTGGGATCTCATAGC

>JTB_2511847_1

ATGTCCTCAAGCGCGCTGGGATCTCATAGCCTG

>JTB_2137601_1

CGACGTCAAAGGATGAAGGACAGTGATAGCTAT

>JTC_1135775_1

GGGATCTCATAGCCTGCATTGGCGTGGTTTGCA

>JTB_3844477_1

GTAATACATCTCCACTTCTTCATACTTTGTCTG

>JTC_2526021_1

AGGATGCCGAGCCGCTTTGCAAGCGAGCTTTGG

>JTB_4559728_1

CTCGGCATCCTTGTATTTGCCACGTTTACCATA

>JTB_399671_2

AAATTTGCTTAATGATGCGCTTGCAATTCGTGA

>JTD_974417_1

CTAGATTCTTCAATGTTGTCGTCACAGTAGGAG

>JTB_287326_2

GATCAACGGTGAAAACAAGCCTATCTGGCAGGT

>JTB_5325887_1

AGCCAGGCATTTTCCTGACATAATCGTCGCACC

>JTB_1744783_1

CTGGGATCTCATAGCCTGCATTGGCGTGGTTTT

>JTB_1369065_1

GCTGGTTCTCTCCAAATCCTCCAGAGCTTGTTT

>JTB_878254_1

GTTTTCACCGTTGATCGCTCCGAATTCCCTCTC

>JTA_1389046_1

CAAGACCAAGAACAATCTTGCAAGCTGCTACCC

>JTC_1803235_1

CTACGGTGAAAACAAGCCTATCTGGCAGGTTGC

>JTB_5195407_1

CAAAAGTTACGTACACAGGTGCGACGATTATGT

>JTC_2983260_2

CGCAATCCTCTAAGGTATCAGCAGCCTCGTACT

>JTD_1772547_2

CGGCATCCTTGTATTTGCCACGTTTACCATAAA

>JTD_148634_2

CATCCTTGTATTTGCCACGTTTACCATAAAGCA

>JTC_1256095_1

GGAATTCGGAGCGATCAACGGTGAAAACAAGCC

>JTC_2297438_1

CATACTTTGTCTGGTTCTGACACAAAAGTGCTA

>JTB_371358_2

CACAGGTGATTGTCGTGCTTGCATCTCATCTAC

>JTB_5018730_1

CCCGGTGTGTTTTCACGATTTTTCGATTTGTTC

>JTC_4542455_1

CGGGCCTCGATATTTTGTAGATGAGATGCAAGC

>JTB_230026_2

GTTGGGGACATGGTATGTCTCTCCTCGGACTCA

>JTB_3135269_2

AAACTGATTTGGTGGTGTTGGGGACATGGTATG

>JTB_2907692_3

CGGACCCAGTGGTGGAACTCTTCCCGGAAGATG

>JTB_3714785_1

GTTCATGGCATCCGTGAAGAAATATGACGATAA

>JTB_4864537_1

CGAGGCCGAAAAGCAAAAGTTACGTACACAGGT

>JTD_5761404_1

AATCGTCAGAGCAGACGGTTGCCCAGCTTGAGG

>JTB_4009305_1

GGAGTTCATGGCATCCGTGAAGAAATATGACGA

>JTB_3114289_2

ATTTGCTTAATGATGCGCTTGCAATTCGTGAAA

>JTB_2887354_3

GGAACTCTTCCCGGAAGATGAGTCCGAGGAGCG

>JTB_74384_5

CAACGGTGAAAACAAGCCTATCTGGCAGGTTGC

>JTB_4629453_1

CGTGCTTGCATCTCATCTACAAAATATCGAGGC

>JTC_3842746_1

GGCAAATACAAGGATGCCGAGCCGCTTTGCAAG

>JTB_513616_1

TTCGTCATAGCTATCACTGTCCTTCATCCTTTG

>JTC_2754654_6

GGATCTCATAGCCTGCATTGGCGTGGTTTGCAA

>JTB_4235908_1

GCCAAGCACATTCTCACGAATTTCCAAAGCTCG

>JTC_1023165_1

GTCCTCAAGCGCGCTGGGATCTCATAGCCTGCA

>JTB_936530_1

GTTGCCGAAGAAAGAGAAGAGAACAAATCGAAA

>JTC_1467945_1

GCAAATTTGCTTAATGATGCGCTTGCAATTCGT

>JTB_2673167_1

AATTTGCTTAATGATGCGCTTGCAATTCGTGAA

>JTD_3516190_1

GTTGGCACAAAGCAGCTAAAGTTGACTCTCCTA

>JTB_2390252_1

CACGTTTACCATAAAGCACAGCTAGATTGTTGA

>JTB_1736020_1

CTGGTTCTCTCCAAATCCTCCAGAGCTTGTTTG

>JTC_1031610_1

GTCCAAGGTTGCGAAAATCCTGGGAGTGTCCGA

>JTB_5260439_1

ATCTGGAGTTCATGGCATCCGTGAAGAAATATG

>JTC_2676783_1

AAATCAGTTTGCAAACCACGCCAATGCAGGCTA

>JTB_2940286_2

TTCATCTCGCAGCCAGGCATTTTCCTGACATAA

>JTC_2766904_5

GGAACTCTTCCCGGAAGATGAGTCCGAGGAGAG

>JTB_4896046_1

CGACAACATTGAAGAATCTAGGCGCCCTCTACA

>JTD_47081_3

GGAGAGTCAACTTTAGCTGCTTTGTGCCAACAG

>JTB_152686_3

CTCGTACTTTCCTTGTCGTCTGTAGAGGGCGCC

>JTC_1425206_1

GCCATGAACTCCAGATGTTTCTTTTCTTCCTCA

>JTB_2927151_3

AACAAATCGAAAAATCGTGAAAACACACCGTAT

>JTC_3540326_1

GTTGGCACAAAGCAGCTAAAGTTGACTCTCCTA

>JTB_773503_1

TCGTGCTTGCATCTCATCTACAAAATATCGAAG

>JTC_5056823_1

ATTTCGTATATTTCCAAGGCTCTTTGGTAATAC

>JTB_2807119_6

CTGCATTGGCGTGGTTTGCAAACTGATTTGGTG

>JTB_1087802_1

GTATGTCTCTCCTCGGACTCATCTTCCGGGAAG

>JTC_1038998_1

GTCACGATCATCCGGACGTCGCAACTATGTTGA

>JTB_1062053_1

GTCCTTCATCCTTTGACGTCTGTTTTCGTCGGA

>JTD_5180822_1

GAGGAAGAAAAGAAACATCTGGAGTTCATGGCA

>JTD_230130_1

TTCCTTGTCGTCTGTAGAGGGCGCCTAGATTCT

>JTB_4515844_1

CTGCTGGGATCTCATAGCCTGCATTGGCGTGGT

>JTB_94035_4

GAAAAATCACGCTCGGACCCAGTGGTGGAACTC

>JTB_2949072_2

TGGAGTTCATGGCATCCGTGAAGAAATATGACG

>JTD_4016022_1

CTCCACTTCTTCATACTTTGTCTGGTTCTGACA

>JTB_1339466_1

GGAAAGTACGAGGCTGCTGATACCTTAGAGGAT

>JTB_2974567_2

GTTGGGGACATGGTATGTCTCTCCTCGGACTCA

>JTD_4460013_1

ATCCTTGTATTTGCCACGTTTACCATAAAGCAC

>JTB_663966_1

TGCTGTGCTTTATGGTAAACGTGGCAAATACAA

>JTB_3827156_1

GTCAACTTTAGCTGCTTTGTGCCAACCGCCATA

>JTB_756263_1

TCTTCATTTTCTTGGTTATCGTCATATTTCTTC

>JTC_5021083_1

CAAATCGAAAAATCGTGAAAACACACCGTATGG

>JTB_808654_1

TCCACTTCTTCATACTTTGTCTGGTTCTGACAC

>JTB_269249_2

GGAAAATGCCTGGCTGCGAGATGAACTAGCAAA

>JTB_3077618_2

CGCCAATGCAGGCTATGAGATCCCAGCGCGCTT

>JTB_3306693_1

TGTGTACGTAACTTTTGCTTTTCGGCCTCGATA

>JTB_288828_2

GAGACATACCATGTCCCCAACACCACCAAATCA

>JTB_1752430_1

CTGGATAACCAGATTGTGCAATGTCCTCAAGCG

>JTB_3629701_1

GTTTGCTAGTTCATCTCGCAGCCAGGCATTTTC

>JTB_1789973_1

CTCTTCCCGGAAGATGAGTCCGAGGAGAGACAT

>JTD_1666965_3

CGCTTTCCCTCTTCGTCATAGCTATCACTGTCC

>JTB_706423_1

TGATGATCCAAACGTCGCCAAGACCAAGAACAA

>JTB_1849379_1

CTAGATTCTTCAATGTTGTCGTCACAGTAGGAG

>JTD_3752449_1

GCAAATTTGCTTAATGATGCGCTTGCAATTCGT

>JTA_1660415_4

ATTTGGTGGTGTTGGGGACATGGTATGTCTCTC

>JTB_159141_3

CGGACCCAGTGGTGGAACTCTTCCCGGAAGATG

>JTB_1400412_1

GCGGTTGGCACAAAGCAGCTAAAGTTGACTCTC

>JTB_4403044_1

CTTTTCTTCCTCAAGCTGGGCAACCGTCTGCTC

>JTB_3933896_1

GGGACCGCTACCTCGTAACGACCTTGAGACGCG

>JTC_4178467_1

GACGAAAACAGACGTCAAAGGATGAAGGACAGT

>JTB_3551407_1

TCACGATCATCCGGACGTCGCAACTATGTTGAA

>JTB_3728360_1

GTTACGTACACAGGTGCGACGATTATGTCAGGG

>JTB_2736830_1

AAACATCTGGAGTTCATGGCATCCGTGAAGAAA

>JTB_2607217_1

AGAGAACCAGCGGTCACGATCATCCGGACGTCG

>JTB_1575102_1

GACGATTATGTCAGGAAAATGCCTGGCTGCGCG

>JTB_259314_2

GGATCTCATAGCCTGCATTGGCGTGGTTTGCCA

>JTC_2669952_1

AACAAATCGAAAAATCGTGAAAACACACCGTAT

>JTC_4206511_1

GAACTCTTCCCGGAAGATGAGTCCGAGGAGAGA

>JTB_3216790_1

TTGGGCTTAGGAGAAGCACAGGTGATTGTCGTG

>JTD_54559_3

CTTCGTCATAGCTATCACTGTCCTTCATCCTTT

>JTB_698051_1

TGATTTTTGCCAAGCACATTCTCACGAATTTCC

>JTB_2821839_5

CTTCAATGTTGTCGTCACAGTAGGAGAGTCAAC

>JTC_4220458_1

GAAAGTACGAGGCTGCTGATACCTTAGAGGATT

>JTB_3372533_1

TGGATTTAGTAAAGCAGTCCAAGGTTGCGAAAC

>JTB_3399718_1

TGCTGGGTGATTTTCGCCTAAGGTTTTTTCACG

>JTC_1561401_1

GAAATTGTTATGAAAAATATTGAAAACATCGAA

>JTB_2619839_1

AGAAGCTCCAATCGTCAGAGCAGACGGTTGCCC

>JTB_5396891_1

ACGAAAACAGACGTCAAAGGATGAAGGACAGTG

>JTB_5013179_1

CCGAAAAGCAAAAGTTACGTACACAGGTGCGAC

>JTB_3849085_1

GTAAAGCAGTCCAAGGTTGCGAAAATCCTGGGA

>JTC_4437841_1

CTCAAGCGCGCTGGGATCTCATAGCCTGCATTG

>JTC_4478541_1

CGTGACCGCTGGTTCTCTCCAAATCCTCCAGAG

>JTC_5060227_1

ATTTATTCTGATCTCTGTATACTAAAGCCAGCA

>JTD_914617_1

CTGATTTGGTGGTGTTGGGGACATGGTATGTCT

>JTB_2655804_1

ACGAAAACAGACGTCAAAGGATGAAGGACAGTG

>JTB_1840712_1

CTCAAGCTGGGCAACCGTCTGCTCTGACGATTG

>JTB_5491211_1

AAAAGCAAAAGTTACGTACACAGGTGCGACGAT

>JTC_2407922_1

CAAATACAAGGATGCCGAGCCGCTTTGCAAGCG

>JTB_5448561_1

AAGAAACATCTGGAGTTCATGGCATCCGTGAAG

>JTB_3413440_1

TGCGCCCTACGCAGCAAAAAAGAGGCGCTGGAT

>JTB_2266747_1

CCGAGCCGCTTTGCAAGCGAGCTTTGGAAATTC

>JTD_5404486_1

CGGACACTCCCAGGATTTTCGCAACCTTGGACT

>JTB_1691479_1

CTTCTTCATACTTTGTCTGGTTCTGACACAAAA

>JTB_1353021_1

GCTTTGCAAGCGAGCTTTGGAAATTCGTGAGAT

>JTB_2913547_3

CCTGCATTGGCGTGGTTTGCAAACTGATTTGGT

>JTA_2737468_1

CGGTCCCACTTTGCAAACAAGCTCTGGAGGCTT

>JTB_2467657_1

CAAAAAAGAGGCGCTGGATTTAGTAAAGCAGTC

>JTC_4937597_1

CAGCCAGGCATTTTCCTGACATAATCGTCGCAC

>JTD_3590919_1

GTCAGGAAAATGCCTGGCTGCGAGATGAACTAG

>JTB_5336804_1

AGATGAACTAGCAAACACACAACAGAAGCTCCA

>JTA_252873_2

CGAAAACAGACGTCAAAGGATGAAGGACAGTGA

>JTC_4320785_1

CTTCAATGTTGTCGTCACAGTAGGAGAGTCAAC

>JTD_1408548_1

ATCCTTGTATTTGCCACGTTTACCATAAAGCAC

>JTB_3715984_1

GTTCAGTTGTTTTGCAACGTCCGGGTGATTTTT

>JTB_4866542_1

CGAGCTTTGGAAATTCGTGAGAATGTGCTTGGC

>JTB_5342852_1

AGAGGATTGCGCCCTACGCAGCAAAAAAGAGGC

>JTC_135898_3

GGAACTCTTCCCGGAAGATGAGTCCGAGGAGAG

>JTA_1143199_1

CGGACGTTGCAAAACAACTGAACAATTTAGCCC

>JTC_2672339_1

AAATTCTGTACAAGCAGGTTCTAACTCGTGCTC

>JTB_4942459_1

CCTCTTCGTCATAGCTATCACTGTCCTTCATCC

>JTB_380391_2

ATTTATTCTGATCTCTGTATACTAAAGCCAGGA

>JTD_4944097_1

GTTGACTCTCCTACTGTGACGACAACATTGAAG

>JTC_901266_1

GTTGCCGAAGAAAGAGAAGAGAACAAATCGAAA

>JTB_2125510_1

CGAGCGTGATTTTTCCTGTTGCTCTTCATTTTC

>JTB_4265996_1

GCAAGCTGCTATCTGAAACAAGGCAAATATAAG

>JTB_271690_2

GCTGGGATCTCATAGCCTGCATTGGCGTGGTTT

>JTC_3064727_1

TTTGCTTTTCGGCCTCGATATTTTGTAGATGAG

>JTB_3152768_1

TTTTCTTGGTTATCGTCATATTTCTTCACGGCT

>JTC_1991367_1

CGCTTTGCAAGCGAGCTTTGGAAATTCGTGAGA

>JTB_2881323_3

GGTTTGCAAACTGATTTGGTGGTGTTGGGGACA

>JTB_1262326_1

GGAGTGTCCGACGAAAACAGACGTCAAAGGATG

>JTD_3443809_1

TCATAGCTATCACTGTCCTTCATCCTTTGACGT

>JTB_313803_2

CTCGTAACGACCTTGAGACGCGTACTGGATAAC

>JTC_1668666_1

CTTCAATGTTGTCGTCACAGTAGGAGAGTCAAC

>JTB_3014546_2

GCTTAATGATGCGCTTGCAATTCGTGAAAAAAC

>JTB_5129532_1

CACGACAATCACCTGTGCTTCTCCTAAGCCCAA

>JTB_201395_2

TGGGATCTCATAGCCTGCATTGGCGTGGTTTGC

>JTB_5232973_1

ATTCTCCATACGGTGTGTTTTCACGATTTTTCG

>JTC_417260_1

TTTGCAAACTGATTTGGTGGTGTTGGGGACATG

>JTC_505902_1

TTCCAAGGCTCTTTGGTAATACATCTCCACTTC

>JTC_675501_1

TGATCTCTGTATACTAAAGCCAGGATGTTCAAC

>JTC_3137636_1

TTCGTCATAGCTATCACTGTCCTTCATCCTTTG

>JTD_2012333_1

TAGCAAACACACAACAGAAGCTCCAATCGTCCG

>JTD_2739303_1

CCGAAGAAAGAGAAGAGAACAAATCGAAAAATC

>JTC_147416_3

CTTCCCGGAAGATGAGTCCGAGGAGAGACATAC

>JTC_3375092_1

TCTGATCTCTGTATACTAAAGCCAGGATGTTCA

>JTB_3333370_1

TGGTTTGCAAACTGATTTGGTGGTGTTGGGGAC

>JTB_1358606_1

GCTTGAGGAAGAAAAGAAACATCTGGAGTTCAT

>JTB_3454969_1

TGAGATGCAAGCACGACAATCACCTGTGCTTCT

>JTD_4213572_1

CCTTGTATTTGCCACGTTTACCATAAAGCACAG

>JTC_4166863_1

GAGAAGCACAGGTGATTGTCGTGCTTGCATCTC

>JTB_4427670_1

CTTGTATTTGCCACGTTTACCATAAAGCACAGC

>JTB_3936472_1

GGGAATTCGGAGCGATCAACGGTGAAAACAAGC

>JTB_3432408_1

TGCAAAACAACTGAACAATTTAGCACTTTTGTG

>JTD_3639822_1

GGGAATTCGGAGCGATCAACGGTGAAAACAAGC

>JTC_2403580_1

CAAATTTGCAGCCTCCTTATATTTATTCTGATC

>JTB_185289_2

TTTGCAAACAAGCTCTGGAGGATTTGGAGAGAA

>JTB_728056_1

TGACGACAACATTGAAGAATCTAGGCGCCCTCT

>JTB_812421_1

TCATAGCCTGCATTGGCGTGGTTTGCAAACTGG

>JTB_1384769_1

GCTCGGCATCCTTGTATTTGCCACGTTTACCAT

>JTB_2887119_3

GGAAGATGAGTCCGAGGAGAGACATACCATGTC

>JTC_2702104_1

AAAACCTTAGGCGAAAATCACCCAGCTGTAGCT

>JTB_3430823_1

TGCAAGCACGACAATCACCTGTGCTTCTCCTAA

>JTB_4246936_1

GCAGGCTATGAGATCCCAGCGCGCTTGAGGACC

>JTB_3559412_1

TATTACCAAAGAGCCTTGGAAATATACGAAATG

>JTB_3432530_1

TGATTTTTCCTGTTGCTCTTCATTTTCTTGGTT

>JTB_986560_1

GTTACGTACACAGGTGCGACGATTATGTCAGGG

>JTC_626310_1

TGGAACTCTTCCCGGAAGATGAGTCCGAGGAGA

>JTD_3054051_1

AAAGCAGCTAAAGTTGACTCTCCTACTGTGACG

>JTB_3748108_1

GTGGAGATGTATTACCAAAGAGCCTTGGAAATA

>JTB_591846_1

TGTAGAGGGCGCCTAGATTCTTCAATGTTGTCG

>JTB_859254_1

TAAACGTGGCAAATACAAGGATGCCGAGCCGCT

>JTD_2699643_1

CCTTCATCCTTTGACGTCTGTTTTCGTCGGACA

>JTB_1378812_1

GCTGATACCTTAGAGGATTGCGCCCTACGCAGC

>JTB_420332_1

TTTTCGCCTAAGGTTTTTTCACGAATTGCAAGC

>JTC_1465651_1

GCAACCTTGGACTGCTTTACTAAATCCAGCGCC

>JTC_5271583_1

AAGAAATATGACGATAACCAAGAAAATGAAGAG

>JTB_5003196_1

CCGAGGAGAGACATACCATGTCCCCAACACCAC

>JTB_5366215_1

AGAACCAGCGGTCACGATCATCCGGACGTCGCA

>JTC_2066335_1

CGATCATCCGGACGTCGCAACTATGTTGAACAT

>JTC_2395449_1

CAACCGCCATATTCTCCATACGGTGTGTTTTCA

>JTC_5063626_1

ATTGTTCTTGGTCTTGGCGACGTTTGGATCATC

>JTC_5175780_1

AGAGAAGAGAACAAATCGAAAAATCGTGAAAAC

>JTB_5491777_1

AAAAGAGGCGCTGGATTTAGTAAAGCAGTCCAA

>JTC_650266_1

TGCCTCCTTATATTTATTCTGATCTCTGTATAC

>JTD_939572_1

CTCGTACTTTCCTTGTCGTCTGTAGAGGGCGCT

>JTB_2347031_1

CAGTGATAGCTATGACGAAGAGGGAAAGCGATC

>JTB_4279292_1

GATTTTCGCAACCTTGGACTGCTTTACTAAATC

>JTB_4647995_1

CGTACTTTCCTTGTCGTCTGTAGAGGGCGCCTA

>JTC_2416430_1

CAAACAAGCTCTGGAGGATTTGGAGAGAACCAG

>JTB_2997486_2

GGGACATGGTATGTCTCTCCTCGGACTCATCTT

>JTB_5146514_1

CAATTTAGCACTTTTGTGTCAGAACCAGACAAA

>JTB_2112611_1

CGATCAACGGTGAAAACAAGCCTATCTGGCAGG

>JTD_285707_1

TGCTAGTTCATCTCGCAGCCAGGCATTTTCCTG

>JTB_3988018_1

GGATTTAGTAAAGCAGTCCAAGGTTGCGAAAAT

>JTB_3381796_1

TGGAGAGAACCAGCGGTCACGATCATCCGGACG

>JTB_2921698_3

ATTTAGTAAAGCAGTCCAAGGTTGCGAAAATCC

>JTB_1606384_1

GAACTCTTCCCGGAAGATGAGTCCGAGGAGAGA

>JTD_2929225_1

ATACCATGTCCCCAACACCACCAAATCAGTTTG

>JTC_5296762_1

AAAGAGCCTTGGAAATATACGAAATGAAGCTTG

>JTB_2753712_1

AAAAAGAGGCGCTGGATTTAGTAAAGCAGTCCA

>JTC_2581143_1

AGAACCAGCGGTCACGATCATCCGGACGTCGCA

>JTC_207222_2

TCTGATCTCTGTATACTAAAGCCAGGATGTTCA

>JTC_5092654_1

ATGAAGGACAGTGATAGCTATGACGAAGAGGGG

>JTB_1694140_1

CTTCTCTGTATACTAAAGCCAGGATGTTCAACA

>JTB_2305086_1

CCAGGATGTTCAACATAGTTGCGACGTCCGGAT

>JTC_2850704_3

CACAAAGCAGCTAAAGTTGACTCTCCTACTGTG

>JTB_2895497_3

GAACTCTTCCCGGAAGATGAGTCCGAGGAGAGA

>JTC_4480256_1

CGTCTGTAGAGGGCGCCTAGATTCTTCAATGTA

>JTC_92304_4

GCGCAATCCTCTAAGGTATCAGCAGCCTCGTAC

>JTB_2742633_1

AAAATATTGAAAACATCGAATTGGGCTTAGGAG

>JTB_2771268_15

GGATCTCATAGCCTGCATTGGCGTGGTTTGCAA

>JTC_3202409_1

TGTCGTGCTTGCATCTCATCTACAAAATATCGA

>JTB_2728843_1

AAAGCCAGGATGTTCAACATAGTTGCGACGTCC

>JTB_2314524_1

CCACCACTGGGTCCGAGCGTGATTTTTCCTGTT

>JTD_293805_1

TGATTTTCGCCTAAGGTTTTTTCACGAATTGCA

>JTB_3433908_1

TGATTGTCGTGCTTGCATCTCATCTACAAAATA

>JTB_5003575_1

CCGAGCGTGATTTTTCCTGTTGCTCTTCATTTT

>JTA_2713738_1

CGTGCTTCTCCTAAGCCCAATTCGATGTTTTCA

>JTC_1223082_1

GGAGGCTGCAAATTTGCTTAATGATGCGCTTGC

>JTB_3104029_2

CAGAATTTCAGCTTCCTTATATTTGCCTTGTTT

>JTD_2672742_1

CGACGTTTGGATCATCAGGCCCAAGCTTCATTT

>JTC_100932_4

CGGAAGATGAGTCCGAGGAGAGACATACCATGT

>JTB_3671909_1

GTTGGAAATGAGGGTTAAGTGTTCAACCCTTAA

>JTB_2371915_1

CAGATGTTTCTTTTCTTCCTCAAGCTGGGCAAC

>JTC_1713733_1

CTGCGTAGGGCGCAATCCTCTAAGGTATCAGCA

>JTB_2454643_1

CAAAGACCAAGAACAATCTTGCAAGCTGCTATC

>JTB_4317524_1

GAGAGACATACCATGTCCCCAACACCACCAAAT

>JTA_1378373_1

CACAAAGCAGCTAAAGTTGACTCTCCTACTGTT

>JTB_5468468_1

AAATATCGAGGCCGAAAAGCAAAAGTTACGTAC

>JTB_247825_2

GGTTTGCAAACTGATTTGGTGGTGTTGGGGACA

>JTB_4605179_1

CTACCTCGTAACGACCTTGAGACGCGTACTGGC

>JTB_5496148_1

AAAACAAGCCTATCTGGCAGGTTGCCGAAGAAA

>JTB_1524391_1

GCAAAAAAGAGGCGCTGGATTTAGTAAAGCAGT

>JTD_2441921_1

CTTCTCTTTCTTCGGCAACCTGCCAGATAGGCT

>JTB_2899514_3

CTTCACGGATGCCATGAACTCCAGATGTTTCTT

>JTD_3888533_1

CTTTCCTTGTCGTCTGTAGAGGGCGCCTAGATT

>JTB_4601262_1

CTAGATTCTTCAATGTTGTCGTCACAGTAGGAG

>JTB_2441328_1

CAACATTGAAGAATCTAGGCGCCCTCTACAGAC

>JTC_2834809_3

CTGGGATCTCATAGCCTGCATTGGCGTGGTTTG

>JTB_1528317_1

GATTTAGTAAAGCAGTCCAAGGTTGCGAAAATC

>JTB_4569563_1

CTCGAACACTAAAACGGTTCTTCAAGGGCTGGA

>JTB_4371000_1

GAAATTCTGTACAAGCAGGTTCTAACTCGTGCT

>JTD_2270783_1

GCCATGAACTCCAGATGTTTCTTTTCTTCCTCA

>JTB_5438245_1

AAGCAGTCCAAGGTTGCGAAAATCCTGGGAGTG

>JTC_151341_3

CTCATAGCCTGCATTGGCGTGGTTTGCAAACTG

>JTB_5449374_1

AACTTTAGCTGCTTTGTGCCAACCGCCATATTC

>JTC_2313918_1

CAGGAAAATGCCTGGCTGCGAGATGAACTAGCA

>JTB_3560072_1

TATGTCTCTCCTCGGACTCATCTTCCGGGAAGA

>JTB_1524091_1

GCAAAAATCACCCGGACGTTGCAAAACAACTGA

>JTB_3127437_2

AGAAAATGAAGAGCAACAGGAAAAATCACGCTC

>JTD_2393890_1

GAAACATCTGGAGTTCATGGCATCCGTGAAGAG

>JTC_2500299_1

ATACGGTGTGTTTTCACGATTTTTCGATTTGTT

>JTD_4891601_1

TAGCAAACACACAACAGAAGCTCCAATCGTCCG

>JTB_3311239_1

TGTGCTTGGCAAAAATCACCCGGACGTTGCAAA

>JTB_596121_1

TGGTTTGCAAACTGATTTGGTGGTGTTGGGGAC

>JTB_135377_3

GGAGGCTGCAAATTTGCTTAATGATGCGCTTGC

>JTB_2394707_1

CACGATTTTTCGATTTGTTCTCTTCTCTTTCTT

>JTB_2130785_1

CGAGAATGTGCTTGGCAAAAATCACCCGGACGT

>JTB_5049342_1

CCACCACTGGGTCCGAGCGTGATTTTTCCTGTT

>JTC_2297226_1

CATAGCCTGCATTGGCGTGGTTTGCAAACTGAT

>JTC_5268035_1

AAGAATCTAGGCGCCCTCTACAGACGACAAGGA

>JTB_913364_1

GTTTACCATAAAGCACAGCTAGATTGTTGAGAG

>JTB_5455255_1

AACCGTTTTAGTGTTCGAGACTATTTCTTCTTG

>JTB_2700337_1

AAGACCCAGATAAGTCGGAAATTGTTATGAAAA

>JTA_3028485_1

CAATCTTGCAAGCTGCTATCTGAAACAAGGCAA

>JTB_3346841_1

TGGTAATACATCTCCACTTCTTCATACTTTGTC

>JTB_238603_2

GTGATTTTTGCCAAGCACATTCTCACGAATTTC

>JTB_4916427_1

CGAACACTAAAACGGTTCTTCAAGGGCTGGAAG

>JTB_376766_2

CAAACTGATTTGGTGGTGTTGGGGACATGGTAT

>JTB_1076334_1

GTCATAGCTATCACTGTCCTTCATCCTTTGACG

>JTC_2924551_2

GGAAGATGAGTCCGAGGAGAGACATACCATGTC

>JTB_4108755_1

GCTTGGCAAAAATCACCCGGACGTTGCAAAACA

>JTB_2476760_1

ATTTGTTCTCTTCTCTTTCTTCGGCAACCTGCC

>JTB_3106924_2

CACAAAGCAGCTAAAGTTGACTCTCCTACTGTG

>JTB_4932855_1

CCTTCATCCTTTGACGTCTGTTTTCGTCGGACA

>JTB_68520_5

CTGGGATCTCATAGCCTGCATTGGCGTGGTTTG

>JTB_720670_1

TGAGAATGTGCTTGGCAAAAATCACCCGGACGT

>JTB_1639429_1

CTTTTTTGCTGCCTTCAGTAAGGTTGGAAATGA

>JTB_381185_2

ATTCTGATCTCTGTATACTAAAGCCAGGATGTT

>JTB_1506444_1

GCACATTCTCACGAATTTCCAAAGCTCGCTTGC

>JTB_385971_2

AGTGATAGCTATGACGAAGAGGGAAAGCGATCT

>JTB_3304928_1

TGTGTGTTTGCTAGTTCATCTCGCAGCCAGGCA

>JTD_4148171_1

CGCCTAAGGTTTTTTCACGAATTGCAAGCGCCT

>JTB_4358636_1

GAAGAATCTAGGCGCCCTCTACAGACGACAAGG

>JTB_952756_1

GTTCTCTCCAAATCCTCCAGAGCTTGTTTGCAA

>JTC_2158347_1

CCTCTGTATACTAAAGCCAGGATGTTCAACATA

>JTB_1639428_1

CTTTTTTGCTGCGTAGGGCGCAATCCTCTAAGG

>JTB_1252963_1

GGATCTCATAGCCTGCATTGGCGTGGTTTGCAC

>JTB_2294845_1

CCCAGCTTGAGGAAGAAAAGAAACATCTGGAGT

>JTD_5546139_1

CATCCGTGAAGAAATATGACGATAACCAAGAAA

>JTC_1511394_1

GAGACGCGTACTGGATAACCAGATTGTGCAATG

>JTB_3143422_1

TTTTTCACGAATTGCAAGCGCATCATTAAGCAA

>JTB_5438416_1

AAGCAGCTAAAGTTGACTCTCCTACTGTGACGA

>JTB_4273074_1

GCAAATACAAGGATGCCGAGCCGCTTTGCAAGC

>JTC_856000_1

GTTTGCTAGTTCATCTCGCAGCCAGGCATTTTC

>JTB_4318834_1

GAGACATACCATGTCCCCAACACCACCAAATCA

>JTA_2224416_1

GTGCAATGTCCTCAAGCGCGCTGGGATCTCATA

>JTC_2547308_1

AGCAAACACACAACAGAAGCTCCAATCGTCAGA

>JTB_5296915_1

AGTCAACTTTAGCTGCTTTGTGCCAACCGCCAT

>JTA_631208_1

GTCCCACTTTGCAAACAAGCTCTGGAGGATTTG

>JTB_4114895_1

GCTTCATTTCGTATATTTCCAAGGCTCTTTGGT

>JTB_3369399_1

TGGCAGCCTCCTTATATTTATTCTGATCTCTGT

>JTB_143412_3

GCAAATTTGCTTAATGATGCGCTTGCAATTCGT

>JTB_4502986_1

CTGGCGTGGTTTGCAAACTGATTTGGTGGTGTT

>JTC_2309968_1

CAGGATGTTCAACATAGTTGCGACGTCCGGATG

>JTC_3795353_1

GGGACATGGTATGTCTCTCCTCGGACTCATCTT

>JTB_5205880_1

ATTTTGTAGATGAGATGCAAGCACGACAATCAC

>JTB_3715110_1

GTTCATCTCGCAGCCAGGCATTTTCCTGACATA

>JTB_2933591_2

TTTCCTTGTCGTCTGTAGAGGGCGCCTAGATTC

>JTB_2141797_1

CGACGATTATGTCAGGAAAATGCCTGGCTGCGA

>JTB_3857450_1

GGTTGGCACAAAGCAGCTAAAGTTGACTCTCCT

>JTD_1346393_1

ATTTTTGCCAAGCACATTCTCACGAATTTCCAA

>JTB_310504_2

CTGCAAATTTGCTTAATGATGCGCTTGCAATTC

>JTB_4119500_1

GCTGTGCTTTATGGTAAACGTGGCAAATACAAG
