# Supplementary material for: Evolutionary analysis of the kinesin light chain genes in the yellow fever mosquito Aedes aegypti: gene duplication as a source for novel early zygotic genes
Source: BMC Evol Biol. 2010 Jul 8;10:206. doi: 10.1186/1471-2148-10-206 (PMC2927918; doi:10.1186/1471-2148-10-206)
Supplement: Additional file 3 — FASTA file of amino acid sequences used in this study. [file 1471-2148-10-206-S3.DOC]

>Aaeg_AAEL011410

MFPVPHSTLLMTDESLLSKMKTVKQSLEALRTELQSIRYGISSTDPISNPEKEEVINRNA

QNIELGLGEAQLLVALLSHLKSIDVERQTLRIQVKGLCQETSWLRDEVKDSQQQLLEAQL

KIVQLEEEKRQMEFAASIKKYDDFLEGVEFDEKFGDDPAFDLFPEDEIAERRLSKSTPTP

SQHYPDYEVSPRLKTIQNLALSYASKGRYEVAVPLCKQALEDLERENGREHPDVATMLSI

LTMVYRDQNNLPEAIKHMNEALGIWVRCLGECHPSVAAALNNLAVLYGKNGNYKEAESLC

KRALANRENVLGRYHPDVAKQLNNLALLCQNQGKHGEVELYIRRALEIFESQLGANDPNA

IKTKCNLAACCLKLRKYKEADQLLRDVLVKTQDKEFDETQNGDSESINVDNTSYGKSGAW

HETAVVHSPTVKSILKHMASLHQRLGNFDVASKLKHGQFRSKQEILDIVDGI

>Aaeg_AAEL014967

MFPVPHSTLLMTDESLLSKMKTVKQSLEALRTELQSIRYGISSTDPISNPEKEEVINRNA

QNIELGLGEAQLLVALLSHLKSIDVERQTLRIQVKGLCQETSWLRDEVKDSQQQLLEAQL

KIVQLEEEKRQMEFTASIKKYDDFLEGVEFDEKFGDDPAFDLFPEDEIAERRLSKSTPTP

SQHYPDYEVSPRLKTIQNLALNYASKGRYEVAVPLCKQALEDLERENGREHPDVATMLSI

LTMVYRDQNNLPEAIKHMNEALGIWVRCLGECHPSVAAALNNLAVLYGKNGNYKEAESLC

KRALANRENVLGRYHPDVAKQLNNLALLCQNQGKHGEVELYIRRALEIFESQLGVIDPNA

IKTKCNLAACCLKLRKYKEADQLLRDVLVKTQDKEFDEVQNGDSESINVDNTSYGKSGAW

HETAVVHSPTVKSILKHMASLHQRLGNFDVSSKLKHGQFRSKQEILDIVDGI

>Aaeg_AAEL012472

MTQMSQEEIVSNTKTVLQGLEALRVEHLTLISNLTEGSKKDPDKSEIVMKNIENIELGLG

EAQVIVVLASHLQNIEAEKQKLRTQVRRLCQENAWLRDELANTQQKLQSSEQTVAQLEEE

KKHLEFMASVKKYDDNQENEEQQEKSRSDPVVELFPEDESEERHTMSPTPPNQFANHANA

GYEIPARLRTLHNLVIQYASQGRYEVAVPLCKQALEDLERTSGHDHPDVATMLNILALVY

RDQNKYKEAANLLNDALAIREKTLGENHPAVAATLNNLAVLYGKRGKYKDAEPLCKRALE

IRENVLGKNHPDVAKQLNNLALLCQNQTKYEEVEMYYQRALEIYEMKLGPDDPNVAKTKN

NLASCYLKQGKYKEAEILYKQVLTRAHEREFGAINGENKPIWQVAEEREENKSKNRENTP

YGEYGGWHKAAKVDSPTVTTTLKNLGALYRRQGKYEAADTLEDCALRSKKEALDLVKQSK

VAKILGVSDENRRQRMKDSDSYDEEGKRSQK

>Aaeg_AAEL005502

MTQMSQEEIVSNTKTVLQGLEALRVEHLTLISNLTEGSKKDPDKSEIVMKNIENIELGLG

EAQVIVVLASHLQNIEAEKQKLRTQVRRLCQENAWLRDELANTQQKLQSSEQTVAQLEEE

KKHLEFMASVKKYDDNQENEEQQEKSRSDPVVELFPEDESEERHTMSPTPPNQFANHANA

GYEIPARLRTLHNLVIQYASQGRYEVAVPLCKQALEDLERTSGHDHPDVATMLNILALVY

RDQVCV

>Cpip_CPIJ002971

MATLTRTQIISNTKISMHVLDALKSQYTTLASHQASKVNPFKLEILSKNLANIEHGLDEA

HLITTLATMLQSLEIEKERLRLQVKRLHQESAWLREELSSAQKRLQTSEEEVIQLQEDKK

HLEFTLSLKKYEDNEDHPLEKSHVDLIEELFTEDCSDGRSPTPPSQYSGYETPPRLRTLY

NLVIEYASQGRYEVAVPLCQQALDDLEKTSGHNHPDVATMLNILALVYRDQEKYREAVKL

LIDALAIREKVLGENHPAVAATLNNLAVLYGKCGKYKDAEPLCRRALIIRRNVLGEDHPD

VAKQLTNLALICENLQKYTEVEKFYRKALEIYEAKLGPEDPNVNKTKHNLGNCYVKLGEY

QKAEALYNQILTNPITPTKDNQESPKPGKSSEAPANSQQQKEQSPAYREYGDWHKAAPAD

SPTVAKTLKKLRALRKKQAVMVDSTDSLKDCALRSKKKALELVARAKAMRHSTGSFL

>Cpip_CPIJ015771

MTQEEIVSNTKTVLQGLEALRVEHLTLVSNLSEGTEGSKREIVRQNIENIELGLGEAQVI

VVLASHLQNIEAEKQKLRTQVRRLCQENAWLRDELANTQQKLQASEQSVAQLEEEKKHLE

FMASVKKYDDNQEHEENLEKSRSDPVVELFPEDESEERQNMSPTPANQFANHANAGYEIP

ARLRTLHNLVIQYASQGRYEVAVPLCKQALEDLEKTSGHDHPDVATMLNILALVYRDQNK

YKEAANLLNDALAIREKTLGENHPAVAATLNNLAVLYGKRGKYKDAEPLCKRALEIRENV

LGKNHPDVAKQLNNLALLCQNQTKYEEVEIYYQRALEIYEMKLGPDDPNVAKTKNNLASC

YLKQGKYKEAEVLYKQVLTRAHEREFGTINGENKPIWQVAEEREENKLKNRENTPYGEYG

GWHKAAKVDSPTVTVTLKNLGALYRRQGKYEAADTLEDCALRSKKEALDLAKQSKVAKIL

GVPEGIGGGGGGRQKMKDSDKDDDESVRRTPKQ

>Agam_AGAP010258

MTQMAQEEIVSNTKTVMQGLEALRVEHVTLMNNLAEGSKTDPDKMEIVKKNMENIELGLS

EAQVIVMLFAHLQNIEAEKQKLRTQVKRLCQENVWLRDELAITQQKLQASEQSVAQLEEE

KKHLEFMASVKKYDEIQENEDNLEKSRTDPVVELFPEDEAEERNNMSPTPPNQFANHANA

GYEIPARLRTLHNLVIQYASQGRYEVAVPLCKQALEDLEKTSGHDHPDVATMLNILALVY

RDQNKYKEAANLLNDALTIREKTLGENHPAVAATLNNLAVLYGKRGKYKDAEPLCKRALE

IRENVLGKSHPDVAKQLNNLALLCQNQAKYEEVEMYYKRALEIYEMKLGGDDPNVAKTLN

NLASCYLKQGKYKEAEMLYKEVLTRAHEREFGAINGENKPIWQVAEEREENKLKNRENTP

YGEYGGWHKAAKVDSPTVTTTLKNLGALYRRQGKYEAADTLEDCALRSKKEALDLVKQSK

VLGVSDDNKRQKSGNSKDDNDDHNKRVHK

>Dmel_NP_524049

MTQMSQDEIITNTKTVLQGLEALRVEHVSIMNGIAEVQKDNEKSDMLRKNIENIELGLSEAQVMMALTSH

LQNIEAEKHKLKTQVRRLHQENAWLRDELANTQQKFQASEQLVAQLEEEKKHLEFMASVKKYDENQEQDD

ACDKSRTDPVVELFPDEENEDRHNMSPTPPSQFANQTSGYEIPARLRTLHNLVIQYASQGRYEVAVPLCK

QALEDLERTSGHDHPDVATMLNILALVYRDQNKYKEAANLLNDALSIRGKTLGENHPAVAATLNNLAVLY

GKRGKYKDAEPLCKRALEIREKVLGKDHPDVAKQLNNLALLCQNQGKYDEVEKYYQRALDIYESKLGPDD

PNVAKTKNNLAGCYLKQGRYTEAEILYKQVLTRAHEREFGAIDSKNKPIWQVAEEREEHKFDNRENTPYG

EYGGWHKAAKVDSPTVTTTLKNLGALYRRQGMFEAAETLEDCAMRSKKEAYDLAKQTKLSQLLTSNEKRR

SKAIKEDLDFSEEKNAKP

>Amel_GB13592-PA

MTAMTQEEIMAGARIVAQGLEALRVEHGGLLQALQTQDAPVARDKASLLSKNIEMIELGL

GEAQVMMALANHLQMVEAEKQKLRTQVRRLCQENAWLRDELAGTQQKLQASEQALVQLEE

QKKHLDFMESMKQYDPDPSADDENAKDRPPDDPVVDLFPDDDADDRNTISPTPPSQFAQQ

VNAGYEIPARLRTLHNLVIQYASQGRYEVAVPLCKQALEDLEKTSGHDHPDVATMLNILA

LVYRDQNKYKEAANLLNDALAIREKTLGENHPAVAATLNNLAVLYGKRGKYKEAEPLCKR

ALDIREKVLGRDHPDVAKQLNNLALLCQNQGKYEEVERYYLRALEIYEGKLGPDDPNVAK

TKNNLASCYLKQGKYKDAEVLYKQVLTRAHEKEFGAIAGDNKPIWQVAEEREENKHRNKE

NTPYGEYGGWHKAAKVDSPTVTTTLKNLGALYRRQGKYEAAETLEDCALRSRKETLEFVK

QGKVAQLLG

>Tcas_XP_966804

MDDSNTKKIESLGKMTAMSQEEIVSAVRTVAQGLEALRSEHAGILHGLHEAPDPVANERAGLVQQSAEMI

ELGLGEAQVIMALANHLQLIEAEKQKLRTQVRRLCQENAWLRDELASTQQRLQASEQTVAQLEEEKRHLE

FMSSVSKYDQDVNDDNTSEHSRSEKPDPVVDLFPDDDNEDRNNMSPTPPNQLQLSQQVNAGYEIPARLRT

LHNLVIQYASQGRYEVAVPLCKQALEDLEKTSGHDHPDVATMLNILALVYRDQNKYKEAANLLNDALAIR

EKTLGENHPAVAATLNNLAVLYGKRGKYKEAEPLCKRALDIREKVLGRDHPDVAKQLNNLALLCQNQGKY

EEVEKYYQRALEIYEKRLGPDDPNVSKTMNNLASCYLKQGKYKEAEVLYKQILNRAHEREFGTIDGDNKP

IWQVAEEREENKAKNRENAPYGEYGGWHKAAKVDSPTVTTTLKNLGALYRRQGKYEAAETLEDCALRSRK

EALDIVKHSKVANILGDMDRRKAGSVKDKTSRRGSRESLDMSYDGEEPNTARTPDMEKGGLKTKLFNALG

INSPVNKPPSS

>Cbri_XP_001674197

MSLAGSTIQAYRQRKIESLAKMSNMSQDDVTTGLRTVQQGLEALREEHSTISNTLETSVKGVKDDEAPLP

KQKLTQINDNLDKLLCGVDETSMMLMVFQLTQGMDAQHQKYQAQRRRLCQENAWLRDELSSTQIKLQASE

QMVAQLEEENKHLKYMASIKQFDDGAVPDTKTVVDSGPQPVTSETLQELGFGPEDEEDLNTTQFAQPTPA

HSMAASAAVGYEIPARLRTLHNLVIQYASQGRYEVAVPLCKQALEDLEKTSGHDHPDVATMLNILALVYR

DQNKYKEAANLLNEALSIREKCLGESHPAVAATLNNLAVLFGKRGKFKDAEPLCKRALEIREKVFGDDHP

DVAKQLNNLALLCQNQGKYEEVEKYYKRALEIYESKLGPDDLNVAKTKNNLSSAYLKQGKYKEAEDLYKQ

ILTRAHEKEYGQVSGDNKTIWQIAEDREENKHKGEGATANEQAGWAKAAKVDNPTVTTTLKNLGALYRRQ

GKYEAAETLEDVALRAKKQFAESRKMDRSGMY

>Spur_Q05090

MSGSKLSTPNNSGGGQGNLSQEQIITGTREVIKGLEQLKNEHNDILNSLYQSLKMLKKDTPGDSNLVEEK

TDIIEKSLESLELGLGEAKVMMALGHHLNMVEAEKQKLRAQVRRLVQENTWLRDELAATQQKLQTSEQNL

ADLEVKYKHLEYMNSIKKYDEDRTPDEEASSSDPLDLGFPEDDDGGQADESYPQPQTGSGSVSAAAGGYE

IPARLRTLHNLVIQYASQSRYEVAVPLCKQALEDLEKTSGHDHPDVATMLNILALVYRDQNKYKEAGNLL

HDALAIREKTLGPDHPAVAATLNNLAVLYGKRGKYKEAEPLCKRALEIREKVLGKDHPDVAKQLNNLALL

CQNQGKYEEVEWYYQRALEIYEKKLGPDDPNVAKTKNNLAAAYLKQGKYKAAETLYKQVLTRAHEREFGL

SADDKDNKPIWMQAEEREEKGKFKDNAPYGDYGGWHKAAKVDSRSRSSPTVTTTLKNLGALYRRQGKYDA

AEILEECAMKSRRNALDMVRETKVRELLGQDLSTDVPRSEAMAKERHHRRSSGTPRHGSTESVSYEKTDG

SEEVSIGVAWKAKRKAKDRSRSIPAGYVEIPRSPPHVLVENGDGKLRRSGSLSKLRASVRRSSTKLLNKL

KGRESDDDGGMKRASSMSVLPSRGNDESTPAPIQLSQRGRVGSHDNLSSRRQSGNF

>Cele_a_P46822.2

MSNMSQDDVTTGLRTVQQGLEALREEHSTISNTLETSVKGVKEDEAPLPKQKLSQINDNLDKLVCGVDET

SLMLMVFQLTQGMDAQHQKYQAQRRRLCQENAWLRDELSSTQIKLQQSEQMVAQLEEENKHLKYMASIKQ

FDDGTQSDTKTSVDVGPQPVTNETLQELGFGPEDEEDMNASQFNQPTPANQMAASANVGYEIPARLRTLH

NLVIQYASQGRYEVAVPLCKQALEDLEKTSGHDHPDVATMLNILALVYRDQNKYKEAANLLNEALSIREK

CLGESHPAVAATLNNLAVLFGKRGKFKDAEPLCKRALEIREKVLGDDHPDVAKQLNNLALLCQNQGKYEE

VEKYYKRALEIYESKLGPDDPNVAKTKNNLSSAYLKQGKYKEAEELYKQILTRAHEREFGQISGENKPIW

QIAEEREENKHKGEGATANEQAGWAKAAKVDSPTVTTTLKNLGALYRRQGKYEAAETLEDVALRAKKQHE

PLRSGAMGGIDEMSQSMMASTIGGSRNSMTTSTSQTGLKNKLMNALGFNS

>Cele_b_NP_502066

MVISLGDDITTVLKTVQQTLFALRDEHEAATRILEANLINSDSSEPSLPSEKMGLIDESLGKVMDGGDEA SLLIMMDKLMQSYDVQLSKNHESIRLLRQENTWLLDELTTTQRKLQESERTVAHLEEERDHYKFQDSMNY LNSDFQHTTSVDATPMMVDTLQELGFGPEEEDQNNNQADQGCRSSSFSNPISNDYQLPTRLQTLQNLVIQ YMEQGRFEVAIPLCKQALEDVVKVHGNVHLDVATMLNVLAIVYRNQENFKDAAIYLEKALSIRVQCCGEN HHSVAATLNNLAIAYGKRGKYKESEPLCKRALEIRKNLLGPNHPDVAKQLTNLGIVTQQLEKYEETENYF KQALSIYNRAFPENHQNVIKTKNQLASVFLKQGKYQEAEEMYKNILSKVAITGNKPIWRIAEDREERQRN GIPKVDDESFNVNPTTVMDSNVMSTIKNLAAVYRKQGKEEAAGTLEEALGAKKQINGGADHTNSTASSVE

TSSAVAINPAQSGIKKRIMHFVGLNF

>Lpea_P46825

MEVTQTVKSYRIKKIEEIGKMTALSQEEIISNTKTVIQGLDTLKNEHNQILNSLLTSMKTIRKENGDTNL

VEEKANILKKSVDSIELGLGEAQVMMALANHLQHTEAEKQKLRAQVRRLCQENAWLRDELANTQQKLQMS

EQKVATIEEEKKHLEFMNEMKKYDTNEAQVNEEKESEQSSLDLGFPDDDDDGGQPEVLSPTQPSAMAQAA

SGGCEIPARLRTLHNLVIQYASQGRYEVAVPLCKQALEDLEKTSGHDHPDVATMLNILALVYRDQGKYKE

AANLLNDALGIREKTLGPDHPAVAATLNNLAVLYGKRGKYKDAEPLCKRALVIREKVLGKDHPDVAKQLN

NLALLCQNQGKYEEVERYYQRALEIYQKELGPDDPNVAKTKNNLASAYLKQGKYKQAEILYKEVLTRAHE

KEFGKVDDDNKPIWMQAEEREENKAKYKDGAPQPDYGSWLKAVKVDSPTVTTTLKNLGALYRRQGKYEAA

ETLEECALRSRKSALEVVRQTKISDVLGSDFSKGQSPKDRKRSNSRDRNRRDSMDSVSYEKSGDGDEHEK

SKLHVGTSHKQ

>Pbor_AAB87735

MTASLGVAAAFELSWHALDEIAQQVAAVFSLFSLVEIPWKLVQQCLPKFDAEELEDILDQMLLGANLLGA

NLLKRVDQGMYQLHQLLREFFVVKREQRADDSDLQQRFYQVVIAEAKRVRDEPEKSLIRESTMMIAHLQE

AMERLARPEQALDLATCLNWLAELYYVAQGRYEEAEPLYVRSLSIHEQQLGADHLDVANSFNNLALLYKE

QGRYEEAEPLYVRSLSIREQQLGTDHLDVATSLNNLAVLYRSQGRYEEAEPLYVRSLSIREQQLGTDHLD

VATSLSNLAVLYQSQGCHHKAEPLLVRALPIWEQQLGTDHPDVATSLNNLAFLYHLQGRYEEAEPLLVRA

LSIREQQLGTDHPDVATSLNNLAVLYHLQGRYEDAEPLLLYSVRIRQEQLPADHPLSAKNLSNLAYLYDL

QGRSGEAEALYLQAIPILSAKLEESHQWRQEASQRFRSLLQKALQENRTDELSDDPMTQSILQELRSTLD

>CKstut_CAJ73558

MSKLINSIKSFRRHGTKIILTLSLVVSFILPAYAQENLWKELNDKTTTLLQEKRYADAIKSGEDALRIAK

ETFPPGHISIAASMNLLGILYRTYTMYDEAEPLFNQALDIYRETNGTDHPTVAYVLQELAEMFLLQDNYA

KAEPLYKQSLGIYENVSGQDNPGIVNILNRLGEIYQHQEKYADAILFYKRALAIEVEIFGNDHPDVASSM

NNLATLYYHNGENTKAESLYKQALEIYENEYGADHPLVATILENMAVFYEGTGKKETAKQLHDRAKKIYS

NYRK

>Ptro_XP_524303

MGKALLPLDPVSWRLPAPRAKFCHSRGAGAPRLNFPRAVGVAGSPAGAWLPAAPPLGGAARGPRRPAQQE

RRGVPGLRDATDRSGAKRGRSRPRSGAAMSVQVAAPGSAGLGPERLSPEELVRQTRQVVQGLEALRAEHH

GLSGHLAEALAGQGPATGLEMLEEKQQVVSHSLEAIELGLGEAQVLLALSAHVGALEAEKQRLRSQARRL

AQENVWLREELEETQRRLRASEESVAQLEEEKRHLEFLGQLRQYDPPAESQQSESPPRRDSLASLFPSEE

EERKGPEAAGAAAAQQGGYEIPARLRTLHNLVIQYAGQGRYEVAVPLCRQALEDLERSSGHCHPDVATML

NILALVYRDQNKYKEAIDLLHDALQIREQTLGPEHPAVAATLNNLAVLYGKRGRYREAEPLCQRALEIRE

KVLGADHPDVAKQLNNLALLCQNQGKFEDVERHYARALSIYEALGGPHDPNVAKTKNNLASAYLKQNKYQ

QAEELYKEILHKEDLPAPLGAPNTGTAGDAEQALRRSSSLSKIRESIRRGSEKLVSRLRGEAAAGAAGMK

RAMSLNTLNVDAPRAPGTQFPSWHLDKAPRTLSASTQDLSPH

>Btau_a_AAI46245

MYDNMSTMVYMKEDKLEKLTQDEIISKTKQVIQGLEALKNEHNSILQSLLETLKCLKKDDESNLVEEKSN

MIRKSLEMLELGLSEAQVMMALSNHLNAVESEKQKLRAQVRRLCQENQWLRDELANTQQKLQKSEQSVAQ

LEEEKKHLEFMNQLKKYDDDISPSEDKDADSTKEPLDDLFPNDDDDPGQGIQQQHSSAAAAAQQGGYEIP

ARLRTLHNLVIQYASQGRYEVAVPLCKQALEDLEKTSGHDHPDVATMLNILALVYRDQNKYKDAANLLND

ALAIREKTLGKDHPAVAATLNNLAVLYGKRGKYKEAEPLCKRALEIREKVLGKDHPDVAKQLNNLALLCQ

NQGKYEEVEYYYQRALEIYQTKLGPDDPNVAKTKNNLASCYLKQGKFKQAETLYKEILTRAHEREFGSVD

DENKPIWMHAEEREECKGKQKDGTSFGEYGGWYKACKVDSPTVTTTLKNLGALYRRQGKFEAAETLEEAA

MRSRKQGLDNVHKQRVAEVLNDPENTEKRRSRESLSVDVVKYESGPDGGEEVSMSVEWNGDGTGSLKRSG

SFSKLRASIRRSSEKLVRKLKGGSARDSEPRNPGMKRASSLNVLNVGGKAAEDHFQARSPTPL

>Btau_b_NP_001069236

MATMVLPREEKLSQDEIVLGTKAVIQGLETLRGEHRALLAPLVAHEASEAEPGSQERCVLLRRSLEAIEL

GLGEAQVILALSSHLGAVESEKQKLRAQVRRLVQENQWLREELAGTQQKLQRSEQAVAQLEEEKQHLLFM

SQIRKLDEDTPPHEDKGDVPKDSLDDLFPSEEEQNPAPSPGGGDVAAQHGGYEIPARLRTLHNLVIQYAS

QGRYEVAVPLCKQALEDLEKTSGHDHPDVATMLNILALVYRDQNKYKEAAHLLNDALAIREKTLGKDHPA

VAATLNNLAVLYGKRGKYKEAEPLCKRALEIREKVLGKFHPDVAKQLSNLALLCQNQGKAEEVEYYYRRA

LEIYATRLGPDDPNVAKTKNNLASCYLKQGKYQDAEALYKEILTRAHEKEFGSVSGDNKPIWMHAEEREE

SKDKRRDSTPYGEYGSWYKACKVDSPTVNTTLRSLGALYRRQGKLEAAHTLEDCASRSRKQGLDPASQTK

VVELLKDGGSGRGDRRGSREVPGGVGPRSEADLEEAGPAAEWSGDGSGSLRRSGSFGKLRDALRRSSEML

VKKLQGGGPQEPPNPRMKRASSLNFLNKSVEEPVQSGGTGLSDSRTLSSSSMDLSRRNSLVG

>Btau_c_Q2TBQ9

MSVQVAAPGGLGLGLERPSPEELVRQTRQVVKGLEALRAEHRGLAGHLAEALAAQGPAAGLELLEEKQQV

VSHSLEAIELGLGEAQVLLALSAHVGALEAEKQRLRAQARRLAQENAWLREELEETQRRLRASEEAVAQL

EEEKSHLEFLGQLRQYDPPAESQQPESPPRRDSLASLFPSEEEERRGPEAVGAAAAQQGGYEIPARLRTL

HNLVIQYAGQGRYEVAVPLCRQALEDLERSSGHCHPDVATMLNILALVYRDQNKYKEATDLLHDALQIRE

QTLGPEHPAVAATLNNLAVLYGKRGRYREAEPLCQRALEIREKVLGADHPDVAKQLNNLALLCQNQGKFE

EVERHYARALSIYEALGGPHDPNVAKTKNNLASAYLKQNKYQQAEELYKEILHREALPAPLGAPNTGTTS

DTQQQTLSRSSSFSKLRESIRRGSEKLVSRLRGEGAAGAAGMKRAMSLSMLNTDGSRAPENQFPRQHLSE

ASRTLSTSTQDLGPR

>Btau_d_AAI05321

EFLPGSCLNATTKGSIQPRQGPPARMSGLVLGQRDEPAGHRLSQEEILGSTRLVSQGLEALHSEHQAVLQ

SLSQTIECLQQGGHEEGLVHEKARQLRRSMENIELGLSEAQVMLALANHLSTVESEKQKLRAQVRRLCQE

NQWLRDELAGTQQRLQRSEQAVAQLEEEKKHLEFLGQLRQYDEDGHAAEEKEGDASKDSLDDLFPNEEEE

DPSNGLSRGQGAQHSGYEIPARLRTLHNLVIQYAAQGRYEVAVPLCKQALEDLERTSGRGHPVVATMLNI

LALVYRGQNKYKEAALLLNDALSIRESTLGRDHPAVAATLNNLAVLYGKRGKYKEAEPLCQRALEIREKV

LGTNHPDVAKQLNNLALLCQNQGKYEAVERYYRRALAIYEGQLGPDNPNVARTKNNLASCYLKQGKYAEA

ETLYKEILTRAHVQEFGSVDDDHKPIWMHAEEREEMSKIRHREGSTPYAEYGGWYKACKVSSPTVNTTLR

NLGALYRRQGKLEAAETLEECALRSRKQGTDPISQTKVAELLGEGDSGRTSQEGLGGSVKFEGGEDASVA

VEWSGDGSGALQRSGSLGKIRDVLRRSSELLVRKLQGSEPRPSSSNMKRAASLNYLNQPSAAPLQVSRGL

SASSMDLSSSS

>Rnor_a_P37285

MHDNMSTMVYMKEEKLEKLTQDEIISKTKQVIQGLEALKNEHNSILQSLLETLKCLKKDDESNLVEEKSS

MIRKSLEMLELGLSEAQVMMALSNHLNAVESEKQKLRAQVRRLCQENQWLRDELANTQQKLQKSEQSVAQ

LEEEKKHLEFMNQLKKYDDDISPSEDKDSDSSKEPLDDLFPNDEDDPGQGIQQQHSSAAAAAQQGGYEIP

ARLRTLHNLVIQYASQGRYEVAVPLCKQALEDLEKTSGHDHPDVATMLNILALVYRDQNKYKDAANLLND

ALAIREKTLGRDHPAVAATLNNLAVLYGKRGKYKEAEPLCKRALEIREKVLGKDHPDVAKQLNNLALLCQ

NQGKYEEVEYYYQRALEIYQTKLGPDDPNVAKTKNNLASCYLKQGKFKQAETLYKEILTRAHEREFGSVD

DENKPIWMHAEEREECKGKQKDGSSFGEYGGWYKACKVDSPTVTTTLKNLGALYRRQGKFEAAETLEEAA

LRSRKQGLDNVHKQRVAEVLNDPENVEKRRSRESLNVDVVKYESGPDGGEEVSMSVEWNGMRKMKLGLVK

>Rnor_b_Q68G30

MSVQVAAPGSTGLGPERLNPEELVRQTRQVVQGLEALRAEHHSLAGHLAEALAGPGPVAGVELLEEKQQV

VNHSLEAIELGLGEAQVLLALSAHVGVLEAEKQRLRAQARRLAQENTWLREELEETQRRLRASEEAVAQL

EEEKSHLQFLGQLRQYDPPEESQRPDSPPRRDSLASLFPSEEEEKKGPEAAGAAAAQQGGYEIPARLRTL

HNLVIQYASQGRYEVAVPLCRQALEDLERSSGHCHPDVATMLNILALVYRDQNKYKEATELLHDALQIRE

QTLGPEHPAVAATLNNLAVLYGKRGRYREAEPLCQRALEIREKVLGADHPDVAKQLNNLALLCQNQGKFQ

DVERHYARALSIYEALGGPQDPNVAKTKNNLASAYLKQNKYQQAEELYKEILSQEALPAPLGAPQGGTAG

EAQQQVLRRSSSFSKLRESIRRGSEKLVSRLRGESMAGAAGMKRAMSLNMLNVDGPRAARMQLSTQHLNE

ASRTLSASTQDLSPR

>Rnor_c_NP_001009601

MSGLVLGQRDEPAGHRLSQEEILGSTRLVSQGLESLHSEHQAVLQSLSHTIECLQQGGHEEGLVHEKARQ

LRRSMENIELGLSEAQVMLALASHLSTVESEKQKLRAQVRRLCQENQWLRDELAGTQQRLQRSEQAVAQL

EEEKKHLEFLRQLRQYDEDGHSMEEKEGDASKDSLDDLFPNEEEEDSSNDLSRGQGAAAAQQGGYEIPAR

LRTLHNLVIQYAAQGRYEVAVPLCKQALEDLERTSGRGHPDVATMLNILALVYRDQNKYKEAAHLLNDAL

SIRESTLGRDHPAVAATLNNLAVLYGKRGKYKEAEPLCQRALEIREKVLGTDHPDVAKQLNNLALLCQNQ

GKYEAVERYYQRALAIYERQLGPDNPNVARTKNNLASCYLKQGKYSEAETLYKEILTRAHVQEFGSVDDD

HKPIWMHAEEREEMSRSRSRESGTPYAEYGGWYKACRVSSPTVNTTLRNLGALYRRQGKLEAAETLEECA

LRSRKQGTDPISQTKVAELLGEGDGRKTMQEGPGDSVKFEGGEDASVAVEWSGDGSGTLQRSGSLGKIRD

VLRRSSELLVRKLQGTEPRPSSSNMKRAASLNYLNQPNAAPLQTSRGLSASTVDLSSSS

>Mmus_a_O88447

MYDNMSTMVYIKEEKLENVTQDEIISKTKQVIQGLEALKNEHNSILQSLLETLKCLKKDDESNLVEEKSS

MIRKSLEMLELGLSEAQVMMALSNHLNAVESEKQNVRAQVRRLCQENQWLRDELANTQQKLQKSEQSVAQ

LEEEKKHLEFMNQLKKYDDDISPSEDKDSDSSKEPLDDLFPNDEDEPGQGIQHSDSSAAAARQGYEIPAR

LRTLHNLVIQYASQGRYEVAVPSCKQALEDLEKTSGHDHPDVATMLNILALVYRDQNKYKDAANLLNDAL

AIREKTLGRDHPAVAATLNNLAVLYGKRGKYKEAEPLCKRALEIREKVLGKDHPDVAKQLNNLALLCQNQ

GKYEEVEYYYQRALGIYQTKLGPDRTPNVAKTKNNLASCYLKQGKFKQAETLYKEILTRAHEAEFGSVDD

ENKPIWMHAEEREECKGKQKDGSAFGEYGGWYKACKVDSPTVTTTLKNLGALYRRQGKFEAAETLEEAAM

RSRKQGLDNVHKQRVAEVLNDPESMEKRRSRESLNMDVVKYESGPDGGEEA

>Mmus_b_O88448

MATMVLPREEKLSQDEIVLGTKAVIQGLETLRGEHRALLAPLASHEAGEAEPGSQERCLLLRRSLEAIEL

GLGEAQVILALSSHLGAVESEKQKLRAQVRRLVQENQWLREELAGTQQKLQRSEQAVAQLEEEKQHLLFM

SQIRKLDEMLPQEEKGDVPKDSLDDLFPNEDEQSPAPSPGGGDVAAQHGGYEIPARLRTLHNLVIQYASQ

GRYEVAVPLCKQALEDLEKTSGHDHPDVATMLNILALVYRDQNKYKDAAHLLNDALAIREKTLGKDHPAV

AATLNNLAVLYGKRGKYKEAEPLCKRALEIREKVLGKFHPDVAKQLSNLALLCQNQGKAEEVEYYYRRAL

EIYATRLGPDDPNVAKTKNNLASCYLKQGKYQDAETLYKEILTRAHEKEFGSVNGENKPIWMHAEEREES

KDKRRDRRPMEYGSWYKACKVDSPTVNTTLRTLGALYRPEGKLEAAHTLEDCASRSRKQGLDPASQTKVV

ELLKDGSGRGHRRGSRDVAGPQSESDLEESGPAAEWSGDGSGSLRRSGSFGKLRDALRRSSEMLVRKLQG

GGPQEPNSRMKRASSLNFLNKSVEEPVQPGGRVFLTAAL

>Mmus_c_NP_666294

MSVQVAAPGSTGLGPERLNPEELVRQTRQVVQGLEALRAEHHSLAGHLAEALAGPGPVAGVELLEEKQQV

VNHSLEAIELGLGEAQVLLALSAHVSVLEAEKQRLRAQARRLAQENTWLREELEETQRRLRASEEAVAQL

EEEKSHLQFLGQLRQYDPPEESQRPESPPRRDSLASLFPSEEEEKKGPEAAGAAAAQQGGYEIPARLRTL

HNLVIQYAGQGRYEVAVPLCRQALEDLERSSGHCHPDVATMLNILALVYRDQNKYKEATELLHDALQIRE

QTLGPEHPAVAATLNNLAVLYGKRGRYREAEPLCQRALEIREKVLGADHPDVAKQLNNLALLCQNQGKFQ

DVERHYARALSIYEALGGPQDPNVAKTKNNLASAYLKQNKYQQAEELYKEILSQEALPAPLGAPQGGTAG

DTQQQVLRRSSSFSKLRESIRRGSEKLVSRLRGEGMAGAAGMKRAMSLNMLNVDGPRAARTQLSQLSTRH

LSEAPRTLSISTQDLSPR

>Mmus_d_Q9DBS5

MSGLVLGQRDEPAGHRLSQEEILGSTKVVSQGLEALHSEHQAVLQSLSHTIECLQQGGHEEGLVHEKARQ

LRRSMENIELGLSEAQVMLALASHLSTVESEKQKLRAQVRRLCQENQWLRDELAGTQQRLQRSEQAVAQL

EEEKKHLEFLRQLRQYDEDGHGMEEKEGEATKDSLDDLFPNEEEEDSGNDLSRGQGAAAAQQGGYEIPAR

LRTLHNLVIQYAAQGRYEVAVPLCKQALEDLERTSGRGHPDVATMLNILALVYRDQNKYKEAAHLLNDAL

SIRESTLGRDHPAVAATLNNLAVLYGKRGKYKEAEPLCQRALEIREKVLGTDHPDVAKQLNNLALLCQNQ

GKYEAVERYYQRALAIYESQLGPDNPNVARTKNNLASCYLKQGKYSEAEALYKEILTCAHVQEFGSVDDD

HKPIWMHAEEREEMSRSRPRDSSAPYAEYGGWYKACRVSSPTVNTTLKNLGALYRRQGKLEAAETLEECA

LRSRKQGTDPISQTKVAELLGEGDGRKAIQEGPGDSVKFEGGEDASVAVEWSGDGSGTLQRSGSLGKIRD

VLRRSSELLVRKLQGTEPRPSSSSMKRAASLNYLNQPNAAPLQVSRGLSASTVDLSSSS

>Pabe_b_NP_001125516

MAMMVLPREEKLGQDEIVLGTKAVIQGLETLRGEHRALLAPLVAPEAGEAEPGSQERCILLRRSLEAIEL

GLGEAQVILALSSHLGAVESEKQKLRAQVRRLVQENQWLREELAGTQQKLQRSEQAVAQLEEEKQHLLFM

SQIRKLDEDASPNEEKGDVPKDTLDDLFPNEDEQSPAPSPGGGDVSGQHGGYEIPARLRTLHNLVIQYAS

QGRYEVAVPLCKQALEDLEKTSGHDHPDVATMLNILALVYRDQNKYKEAAHLLNDALAIREKTLGKDHPA

VAATLNNLAVLYGKRGKYKEAEPLCKRALEIREKVLGKFHPDVAKQLSNLALLCQNQGKAEEVEYYYRRA

LEIYATRLGPDDPNVA

>Pabe_c_Q5R8E2

MSVQVAAPGSAGLGPERLSPEELVRQTRQVVQGLEALRAEHHGLVGHLAEALAGQGPVTGLEMLEEKQQV

VSHSLEAIELGLGEAQVLLALSAHVGALEAEKQRLRSQARRLAQENVWLREELEETQRRLRASEEAVAQL

EEEKRHLEFLGQLRQYDPPAESQQSESPPRRDSLASLFPSEEEERKGPEAAGAAAAQQGGYEIPARLRTL

HNLVIQYAGQGRYEVAVPLCRQALEDLERSSGHCHPDVATMLNILALVYRDQNKYKEATDLLHDALQIRE

QTLGPEHPAVAATLNNLAVLYGKRGRYREAEPLCQRALEIREKVLGADHPDVAKQLNNLALLCQNQGKFE

DVERHYARALSIYEALGGPHDPNVAKTKNNLASAYLKQNKYQQAEELYKEILHKEDLPAPLGAPNTGTAG

DAEQALRRSSSLSKIRESIRRGSEKLVSRLRGEGAAGAAGMKRAMSLNTLNVDAPRALGTQFPSWHLDKA

PRTLSASTHDLSPH

>Pabe_a_Q5R581

MYDNMSTMVYIKEDKLEKLTQDEIISKTKQVIQGLEALKNEHNSILQSLLETLKCLKKDDESNLVEEKSN

MIRKSLEMLELGLSEAQVMMALSNHLNAVESEKQKLRAQVRRLCQENQWLRDELANTQQKLQKSEQSVAQ

LEEEKKHLEFMNQLKKYDDDISPSEDKDTDSTKEPLDDLFPNDEDDPGQGIQQQHSSAAAAAQQGDYEIP

ARLRTLHNLVIQYASQGRYEVAVPLCKQALEDLEKTSGHDHPDVATMLNILALVYRDQNKYKDAANLLND

ALAIREKTLGKDHPAVAATLNNLAVLYGKRGKYKEAEPLCKRALEIREKVLGKDHPDVAKQLNNLALLCQ

NQGKYEEVEYYYQRALEIYQTKLGPDDPNVAKTKNNLASCYLKQGKFKQAETLYKEILTRAHEREFGSVD

DENKPIWMHAEEREECKGKQKDGTSFGEYGGWYKACKVDSPTVTTTLKNLGALYRRQGKFEAAETLEEAA

MRSRKQGLDNVHKQRVAEVLNDPENMEKRRSRESLNVDVVKYESGPDGGEEVSMSVEWNGMRKMKLGLVK

>Hsap_d_Q9NSK0

MSGLVLGQRDEPAGHRLSQEEILGSTRLVSQGLEALRSEHQAVLQSLSQTIECLQQGGHEEGLVHEKARQ

LRRSMENIELGLSEAQVMLALASHLSTVESEKQKLRAQVRRLCQENQWLRDELAGTQQRLQRSEQAVAQL

EEEKKHLEFLGQLRQYDEDGHTSEEKEGDATKDSLDDLFPNEEEEDPSNGLSRGQGATAAQQGGYEIPAR

LRTLHNLVIQYAAQGRYEVAVPLCKQALEDLERTSGRGHPDVATMLNILALVYRDQNKYKEAAHLLNDAL

SIRESTLGPDHPAVAATLNNLAVLYGKRGKYKEAEPLCQRALEIREKVLGTNHPDVAKQLNNLALLCQNQ

GKYEAVERYYQRALAIYEGQLGPDNPNVARTKNNLASCYLKQGKYAEAETLYKEILTRAHVQEFGSVDDD

HKPIWMHAEEREEMSKSRHHEGGTPYAEYGGWYKACKVSSPTVNTTLRNLGALYRRQGKLEAAETLEECA

LRSRRQGTDPISQTKVAELLGESDGRRTSQEGPGDSVKFEGGEDASVAVEWSGDGSGTLQRSGSLGKIRD

VLRRSSELLVRKLQGTEPRPSSSNMKRAASLNYLNQPSAAPLQVSRGLSASTMDLSSSS

>Hsap_b_Q9H0B6

MAMMVFPREEKLSQDEIVLGTKAVIQGLETLRGEHRALLAPLVAPEAGEAEPGSQERCILLRRSLEAIEL

GLGEAQVILALSSHLGAVESEKQKLRAQVRRLVQENQWLREELAGTQQKLQRSEQAVAQLEEEKQHLLFM

SQIRKLDEDASPNEEKGDVPKDTLDDLFPNEDEQSPAPSPGGGDVSGQHGGYEIPARLRTLHNLVIQYAS

QGRYEVAVPLCKQALEDLEKTSGHDHPDVATMLNILALVYRDQNKYKEAAHLLNDALAIREKTLGKDHPA

VAATLNNLAVLYGKRGKYKEAEPLCKRALEIREKVLGKFHPDVAKQLSNLALLCQNQGKAEEVEYYYRRA

LEIYATRLGPDDPNVAKTKNNLASCYLKQGKYQDAETLYKEILTRAHEKEFGSVNGDNKPIWMHAEEREE

SKDKRRDSAPYGEYGSWYKACKVDSPTVNTTLRSLGALYRRQGKLEAAHTLEDCASRNRKQGLDPASQTK

VVELLKDGSGRRGDRRSSRDMAGGAGPRSESDLEDVGPTAEWNGDGSGSLRRSGSFGKLRDALRRSSEML

VKKLQGGTPQEPPNPRMKRASSLNFLNKSVEEPTQPGGTGLSDSRTLSSSSMDLSRRSSLVG

>Hsap_a_Q07866

MSTMVYIKEDKLEKLTQDEIISKTKQVIQGLEALKNEHNSILQSLLETLKCLKKDDESNLVEEKSNMIRK

SLEMLELGLSEAQVMMALSNHLNAVESEKQKLRAQVRRLCQENQWLRDELANTQQKLQKSEQSVAQLEEE

KKHLEFMNQLKKYDDDISPSEDKDTDSTKEPLDDLFPNDEDDPGQGIQQQHSSAAAAAQQGGYEIPARLR

TLHNLVIQYASQGRYEVAVPLCKQALEDLEKTSGHDHPDVATMLNILALVYRDQNKYKDAANLLNDALAI

REKTLGKDHPAVAATLNNLAVLYGKRGKYKEAEPLCKRALEIREKVLGKDHPDVAKQLNNLALLCQNQGK

YEEVEYYYQRALEIYQTKLGPDDPNVAKTKNNLASCYLKQGKFKQAETLYKEILTRAHEREFGSVDDENK

PIWMHAEEREECKGKQKDGTSFGEYGGWYKACKVDSPTVTTTLKNLGALYRRQGKFEAAETLEEAAMRSR

KQGLDNVHKQRVAEVLNDPENMEKRRSRESLNVDVVKYESGPDGGEEVSMSVEWNGGVSGRASFCGKRQQ

QQWPGRRHR

>Hsap_c_AAH73841

MSVQVAAPGSAGLGPERLSPEELVRQTRQVVQGLEALRAEHHGLAGHLAEALAGQGPAAGLEMLEEKQQV

VSHSLEAIELGLGEAQVLLALSAHVGALEAEKQRLRSQARRLAQENVWLREELEETQRRLRASEESVAQL

EEEKRHLEFLGQLRQYDPPAESQSESPPRRDSLASLFPSEEEERKGPEAAGAAAAQQGGYEIPARLRTLH

NLVIQYAGQGRYEVAVPLCRQALEDLERSSGHCHPDVATMLNILALVYRDQNKYKEATDLLHDALQIREQ

TLGPEHPAVAATLNNLAVLYGKRGRYREAEPLCQRALEIREKVLGADHPDVAKQLNNLALLCQNQGKFED

VERHYARALSIYEALGGPHDPNVAKTKNNLASAYLKQNKYQQAEELYKEILHKEDLPAPLGAPNTGTAGD

AEQALRRSSSLSKIRESIRRGSEKLVSRLRGEAAAGAAGMKRAMSLNTLNVDAPRAPGTQFPSWHLDKAP

RTLSASTQDLSPH

>Xlav_AAH43636

RTRGLPQEGKPLHLTPSESMSVMMYPREEKLDKLSQEEIISNTKLVMQGLEALRNEHNSILHSLLETIKC

LKKDEEANLVHEKSSLLRKSVEMIELGLGEAQLMMALSNHLTAVESEKQKLRAQVRRLCQENQWLRDELA

NTQQKLQHSEQNVAQLEEEKKHLEFMNQLKKYDEDVSPTEEKEGDSAKDNLDELFPNEEDDSGQGIPHQH

GSAAAAAAQQGGYEIPARLRTLHNLVIQYASQGRYEVAVPLCKQALEDLEKTSGHDHPDVATMLNILALV

YRDQNKYKEAAHLLNDALSIREKTLGKDHPAVAATLNNLAVLYGKRGKYREAEPLCKRALEIREKVLGKD

HPDVAKQLNNLALLCQNQGKYDEVEYYYCRALEIYQARLGPDDPNVAKTKNNLASCYLKQGKYKAAEQLY

KDILTQAHVKEFGSVDDDHKPIWMHAEEREEMSKTPHNDSTPYSEYGGWYKACRVNSPTVNTTLRNLGAL

YRRQGKLEAAETLEECAQRSRRQGLDPANQTKVAQLLKEGDTPEQRRKGRETPRSSVKYESGTEGGEEVS

MGGVEWSGDGSGMLRRSGSLGKIRDVLRRSSEMLVKKLQGAAPAEPRSTNMKRAASLNYLNKSSDETFQV

SQNLLSETRGLSASNIDLSSHS

>Ggal_b_XP_001233279

MSTMVYPREEKLDKLSQEEIISNTKLVMQGLEALKNEHNSILHSLLETIKCLKKDEEANLVHEKSNLLRK

SVEMIELGLGEAQVMMALSNHLNAVESEKQKLRAQVRRLCQENQWLRDELANTQQKLQRSEQTVAQLEEE

KKHLEFMNQLKKYDEDVSPSEEKEGDSTKDSLDDLFPNEEEEHGPGLPHQHSSAVAAAQQGGYEIPARLR

TLHNLVIQYASQGRYEVAVPLCKQALEDLEKTSGHDHPDVATMLNILALVYRDQNKYKEAAHLLNDALCF

TEKLISIQVAATLNNLAVLYGKRGKYKEAEPLCKRALEIREKVLGKDHPDVAKQLNNLALLCQNQGKYEE

VEYYYCRALEIYESCLGPDDPNVAKTKNNLASCYLKQGKYKDAEVLYKEILTRAHVKEFGSVDGEHKPIW

MHAEEREEMSKSKHRDSAPYAEYGGWYKACKVSSPTVNTTLRNLGALYRRQGKLEAAETLEECAVRSRRQ

GIDPINQTKVVEILKEGDGTERRRSLGGSVKYENATDGSEEVSMGVEWSGDGSGTLQRSSSLGKIREVIR

RSSEMLVKKLQGNGPLEPRNTSMKRAASLNYLHKSSDASFEGTQGLRAESRGLSASSMDLSSHSSLLASN

>Ggal_a_AAA90972

MYENMSTMVYLKEEKLEKLTQDEIIAKTKQVINGLEALKNEHNSILQSLLETLKCLKKDDETNLVEEKSN

MIRKSLEMLELGLSEAQVMMALSNHLNAVESEKQKLRAQVRRLCQENQWLRDELANTQQKLQKSEQSVAQ

LEEEKKHLEFNSSIAVQQLLQQGGYEIPARLRTLHNLVIQYASQGRYEVAVPLCKQALEDLEKTSGHDHP

DVATMLNILALVYRDQNKYKDAANLLNDALAIREKTLGKDHPAVAATLNNLAVLYGKRGKYKEAEPLCKR

ALEIREKVLGKDHPDVAKQLNNLALLCQNQGKYEEVEYYYQRALEIYQTKLGPDDPNVAKTKNNLASCYL

KQGKFKQAETLYKEILTRAHEREFGSVDDENKPIWMHAEEREECKGKQKDGTSFGEYGGWYKACKVDSPT

VTTTLKNLGALYRRQGKFEAAETLEEAAMRSRKQGLDNVHKQRVAEVLNDPESIEKSEAEKVSMLMW

>Drer_AAI55319

MSTMVCVREEKLEKLSQEEIISNTRLVVQGLEALKSEHTSILQSLTETLRCLKKEEESSLVQEKTSLLRR

SVEMIELGLGEAQVMMALSNHLNAVESEKQKLRAQVRRLCQENQWLRDELANTQQKLQRSEQSVAQLEED

KKQLEFMNMLKKYDEDASPAEEKDGEPPKDSLDDLFPNDEEEHTQGMTQQHNSSAVAAAAQGGYEIPARL

RTLHNLVIQYASQGRYEVAVPLCKQALEDLEKTSGHDHPDVATMLNILALVYRDQNKYKEAAHLLNDALS

IREKTLGKDHPAVAATLNNLAVLYGKRGKYKEAEPLCKRALEIREKVLGKDHPDVAKQLNNLALLCQNQG

KYEEVEYYYCRALEIYECRLGPDDPNVAKTKNNLASCFLKQGKYKEAEVLYKEILTRAHEKEFGSVDAEN

KPIWMHAEEREEMSKGKHRDNTPYGEYGGWYKACKVNSPTVNTTLRNLGALYRRQGKMEAAETLEECAMR

SRKQGLDPIHQTRVVELLKESDASERRSSRECVSSVRFESGSEGAQEVSMAIEWSGDGSGALQRSGSIGR

LRDVLRRSSEMLVKKLQGNTPADTHSNNMKRAASLNFLNKAGDESYQNVSSRRLRDSRGLSSSNVDLYNG

S

>Nvit_XP_001600608

MTAMTQEEIVAGARTVAQGLEALRVEHTGLLNGLQSQEAPAARDKASIISKNIDMIELGLGEAQVMLALA

SHLQMVEAEKQKLRTQVRRLCQENAWLRDELAGTQQKLQASEQAVAQLEEEKRHLEFMASMRQYDPDPQP

DDENAKDRPKDDPVVDLFPDDENEDRNTMSPTPPSQFAQQVTAGYEIPARLRTLHNLVIQYASQGRYEVA

VPLCKQALEDLEKTSGHDHPDVATMLNILALVYRDQNKYKEAANLLNDALAIREKTLGENHPAVAATLNN

LAVLYGKRGKYKEAEPLCKRALEIREKVLGRDHPDVAKQLNNLALLCQNQGKYEEVEQYYQRALEIYEEK

LGPDDPNVAKTKNNLASCYLKQGKYKDAEVLYKQVLTRAHEREFGAIDGDNKPIWQVAEEREENKHRKRE

NAPYGEYGGWHKAAKVDSPTFNTTLRNLGALYRRQGKYEAAETLDDCAIRSRREALDLVKQPKVAQILGD

EKGSNRRGSRTSLNNSEHEQHDECLDQNGEFAAQLSKKKVIVDIILLITAIAADAVTALES
