# Supplementary material for: Evolutionary analysis of the kinesin light chain genes in the yellow fever mosquito Aedes aegypti: gene duplication as a source for novel early zygotic genes
Source: BMC Evol Biol. 2010 Jul 8;10:206. doi: 10.1186/1471-2148-10-206 (PMC2927918; doi:10.1186/1471-2148-10-206)
Supplement: Additional file 4 — FASTA file of amino acid sequence alignment used for phylogenetic inference. [file 1471-2148-10-206-S4.DOC]

>Aaeg_AAEL011410

LLMTDESLLSKMKTVKQSLEALRTELQSIRYGISSTDPISNPEK----------EEVINR

NAQNIELGLGEAQLLVALLSHLKSIDVERQTLRIQVKGLCQETSWLRDEVKDSQQQLLEA

QLKIVQLEEEKRQMEFAASIKKYDDFLEGVEFDEKFGD------DPAFDL-FPEDEIAER

R------LSKSTPTPSQHYPDYEVSPRLKTIQNLALSYASKGRYEVAVPLCKQALEDLER

ENGREHPDVATMLSILTMVYRDQNNLPEAIKHMNEALGIWVRCLGECHPSVAAALNNLAV

LYGKNGNYKEAESLCKRALANRENVLGRYHPDVAKQLNNLALLCQNQGKHGEVELYIRRA

LEIFESQLGAN-DPNAIKTKCNLAACCLKLRKYKEADQLLRDVLV

>Aaeg_AAEL014967

LLMTDESLLSKMKTVKQSLEALRTELQSIRYGISSTDPISNPEK----------EEVINR

NAQNIELGLGEAQLLVALLSHLKSIDVERQTLRIQVKGLCQETSWLRDEVKDSQQQLLEA

QLKIVQLEEEKRQMEFTASIKKYDDFLEGVEFDEKFGD------DPAFDL-FPEDEIAER

R------LSKSTPTPSQHYPDYEVSPRLKTIQNLALNYASKGRYEVAVPLCKQALEDLER

ENGREHPDVATMLSILTMVYRDQNNLPEAIKHMNEALGIWVRCLGECHPSVAAALNNLAV

LYGKNGNYKEAESLCKRALANRENVLGRYHPDVAKQLNNLALLCQNQGKHGEVELYIRRA

LEIFESQLGVI-DPNAIKTKCNLAACCLKLRKYKEADQLLRDVLV

>Hsap_d_Q9NSK0

HRLSQEEILGSTRLVSQGLEALRSEHQAVLQSLSQTIECLQQGG-HEEGLVHEKARQLRR

SMENIELGLSEAQVMLALASHLSTVESEKQKLRAQVRRLCQENQWLRDELAGTQQRLQRS

EQAVAQLEEEKKHLEFLGQLRQYDEDGHTSEEKEGDAT-----KDSLDDL-FPNEEEEDP

SNG----LSRGQGATAAQQGGYEIPARLRTLHNLVIQYAAQGRYEVAVPLCKQALEDLER

TSGRGHPDVATMLNILALVYRDQNKYKEAAHLLNDALSIRESTLGPDHPAVAATLNNLAV

LYGKRGKYKEAEPLCQRALEIREKVLGTNHPDVAKQLNNLALLCQNQGKYEAVERYYQRA

LAIYEGQLGPD-NPNVARTKNNLASCYLKQGKYAEAETLYKEILT

>Btau_b_NP_001069236

EKLSQDEIVLGTKAVIQGLETLRGEHRALLAPLVAHEASEAEPG-SQE-----RCVLLRR

SLEAIELGLGEAQVILALSSHLGAVESEKQKLRAQVRRLVQENQWLREELAGTQQKLQRS

EQAVAQLEEEKQHLLFMSQIRKLDEDTPPHEDKGDVP------KDSLDDL-FPSEEEQNP

AP------SPGGGDVAAQHGGYEIPARLRTLHNLVIQYASQGRYEVAVPLCKQALEDLEK

TSGHDHPDVATMLNILALVYRDQNKYKEAAHLLNDALAIREKTLGKDHPAVAATLNNLAV

LYGKRGKYKEAEPLCKRALEIREKVLGKFHPDVAKQLSNLALLCQNQGKAEEVEYYYRRA

LEIYATRLGPD-DPNVAKTKNNLASCYLKQGKYQDAEALYKEILT

>Hsap_b_Q9H0B6

EKLSQDEIVLGTKAVIQGLETLRGEHRALLAPLVAPEAGEAEPG-SQE-----RCILLRR

SLEAIELGLGEAQVILALSSHLGAVESEKQKLRAQVRRLVQENQWLREELAGTQQKLQRS

EQAVAQLEEEKQHLLFMSQIRKLDEDASPNEEKGDVP------KDTLDDL-FPNEDEQSP

AP------SPGGGDVSGQHGGYEIPARLRTLHNLVIQYASQGRYEVAVPLCKQALEDLEK

TSGHDHPDVATMLNILALVYRDQNKYKEAAHLLNDALAIREKTLGKDHPAVAATLNNLAV

LYGKRGKYKEAEPLCKRALEIREKVLGKFHPDVAKQLSNLALLCQNQGKAEEVEYYYRRA

LEIYATRLGPD-DPNVAKTKNNLASCYLKQGKYQDAETLYKEILT

>Cbri_XP_001674197

SNMSQDDVTTGLRTVQQGLEALREEHSTISNTLETSVKGVKDDE---APLPKQKLTQIND

NLDKLLCGVDETSMMLMVFQLTQGMDAQHQKYQAQRRRLCQENAWLRDELSSTQIKLQAS

EQMVAQLEEENKHLKYMASIKQFDDGAVPDTKTVVDSGPQPVTSETLQELGFGPEDEEDL

NTTQFAQPTPAHSMAASAAVGYEIPARLRTLHNLVIQYASQGRYEVAVPLCKQALEDLEK

TSGHDHPDVATMLNILALVYRDQNKYKEAANLLNEALSIREKCLGESHPAVAATLNNLAV

LFGKRGKFKDAEPLCKRALEIREKVFGDDHPDVAKQLNNLALLCQNQGKYEEVEKYYKRA

LEIYESKLGPD-DLNVAKTKNNLSSAYLKQGKYKEAEDLYKQILT

>Agam_AGAP010258

TQMAQEEIVSNTKTVMQGLEALRVEHVTLMNNLAEG---SKTDP--------DKMEIVKK

NMENIELGLSEAQVIVMLFAHLQNIEAEKQKLRTQVKRLCQENVWLRDELAITQQKLQAS

EQSVAQLEEEKKHLEFMASVKKYDEIQENEDNLEKSRT------DPVVEL-FPEDEAEER

NNMSP-TPPN--QFANHANAGYEIPARLRTLHNLVIQYASQGRYEVAVPLCKQALEDLEK

TSGHDHPDVATMLNILALVYRDQNKYKEAANLLNDALTIREKTLGENHPAVAATLNNLAV

LYGKRGKYKDAEPLCKRALEIRENVLGKSHPDVAKQLNNLALLCQNQAKYEEVEMYYKRA

LEIYEMKLGGD-DPNVAKTLNNLASCYLKQGKYKEAEMLYKEVLT

>Btau_a_AAI46245

EKLTQDEIISKTKQVIQGLEALKNEHNSILQSLLETLKCLKKDD-ESN-LVEEKSNMIRK

SLEMLELGLSEAQVMMALSNHLNAVESEKQKLRAQVRRLCQENQWLRDELANTQQKLQKS

EQSVAQLEEEKKHLEFMNQLKKYDDDISPSEDKDADST-----KEPLDDL-FPNDDDDPG

QGI-Q--QQHSSAAAAAQQGGYEIPARLRTLHNLVIQYASQGRYEVAVPLCKQALEDLEK

TSGHDHPDVATMLNILALVYRDQNKYKDAANLLNDALAIREKTLGKDHPAVAATLNNLAV

LYGKRGKYKEAEPLCKRALEIREKVLGKDHPDVAKQLNNLALLCQNQGKYEEVEYYYQRA

LEIYQTKLGPD-DPNVAKTKNNLASCYLKQGKFKQAETLYKEILT

>Ggal_a_AAA90972

EKLTQDEIIAKTKQVINGLEALKNEHNSILQSLLETLKCLKKDD-ETN-LVEEKSNMIRK

SLEMLELGLSEAQVMMALSNHLNAVESEKQKLRAQVRRLCQENQWLRDELANTQQKLQKS

EQSVAQLEEEKKHLEFN-------------------------------------------

--------SSIAVQQLLQQGGYEIPARLRTLHNLVIQYASQGRYEVAVPLCKQALEDLEK

TSGHDHPDVATMLNILALVYRDQNKYKDAANLLNDALAIREKTLGKDHPAVAATLNNLAV

LYGKRGKYKEAEPLCKRALEIREKVLGKDHPDVAKQLNNLALLCQNQGKYEEVEYYYQRA

LEIYQTKLGPD-DPNVAKTKNNLASCYLKQGKFKQAETLYKEILT

>Aaeg_AAEL012472

TQMSQEEIVSNTKTVLQGLEALRVEHLTLISNLTEG---SKKDP--------DKSEIVMK

NIENIELGLGEAQVIVVLASHLQNIEAEKQKLRTQVRRLCQENAWLRDELANTQQKLQSS

EQTVAQLEEEKKHLEFMASVKKYDDNQENEEQQEKSRS------DPVVEL-FPEDESEER

HTMSP-TPPN--QFANHANAGYEIPARLRTLHNLVIQYASQGRYEVAVPLCKQALEDLER

TSGHDHPDVATMLNILALVYRDQNKYKEAANLLNDALAIREKTLGENHPAVAATLNNLAV

LYGKRGKYKDAEPLCKRALEIRENVLGKNHPDVAKQLNNLALLCQNQTKYEEVEMYYQRA

LEIYEMKLGPD-DPNVAKTKNNLASCYLKQGKYKEAEILYKQVLT

>Hsap_a_Q07866

EKLTQDEIISKTKQVIQGLEALKNEHNSILQSLLETLKCLKKDD-ESN-LVEEKSNMIRK

SLEMLELGLSEAQVMMALSNHLNAVESEKQKLRAQVRRLCQENQWLRDELANTQQKLQKS

EQSVAQLEEEKKHLEFMNQLKKYDDDISPSEDKDTDST-----KEPLDDL-FPNDEDDPG

QGI-Q--QQHSSAAAAAQQGGYEIPARLRTLHNLVIQYASQGRYEVAVPLCKQALEDLEK

TSGHDHPDVATMLNILALVYRDQNKYKDAANLLNDALAIREKTLGKDHPAVAATLNNLAV

LYGKRGKYKEAEPLCKRALEIREKVLGKDHPDVAKQLNNLALLCQNQGKYEEVEYYYQRA

LEIYQTKLGPD-DPNVAKTKNNLASCYLKQGKFKQAETLYKEILT

>Amel_GB13592-PA

TAMTQEEIMAGARIVAQGLEALRVEHGGLLQALQTQD--APVAR--------DKASLLSK

NIEMIELGLGEAQVMMALANHLQMVEAEKQKLRTQVRRLCQENAWLRDELAGTQQKLQAS

EQALVQLEEQKKHLDFMESMKQYDPDPSADDENAKDRP----PDDPVVDL-FPDDDADDR

NTISP-TPPS--QFAQQVNAGYEIPARLRTLHNLVIQYASQGRYEVAVPLCKQALEDLEK

TSGHDHPDVATMLNILALVYRDQNKYKEAANLLNDALAIREKTLGENHPAVAATLNNLAV

LYGKRGKYKEAEPLCKRALDIREKVLGRDHPDVAKQLNNLALLCQNQGKYEEVERYYLRA

LEIYEGKLGPD-DPNVAKTKNNLASCYLKQGKYKDAEVLYKQVLT

>Xlav_AAH43636

DKLSQEEIISNTKLVMQGLEALRNEHNSILHSLLETIKCLKKDE-EAN-LVHEKSSLLRK

SVEMIELGLGEAQLMMALSNHLTAVESEKQKLRAQVRRLCQENQWLRDELANTQQKLQHS

EQNVAQLEEEKKHLEFMNQLKKYDEDVSPTEEKEGDSA-----KDNLDEL-FPNEEDDSG

QGIPH--QHGSAAAAAAQQGGYEIPARLRTLHNLVIQYASQGRYEVAVPLCKQALEDLEK

TSGHDHPDVATMLNILALVYRDQNKYKEAAHLLNDALSIREKTLGKDHPAVAATLNNLAV

LYGKRGKYREAEPLCKRALEIREKVLGKDHPDVAKQLNNLALLCQNQGKYDEVEYYYCRA

LEIYQARLGPD-DPNVAKTKNNLASCYLKQGKYKAAEQLYKDILT

>Aaeg_AAEL005502

TQMSQEEIVSNTKTVLQGLEALRVEHLTLISNLTEG---SKKDP--------DKSEIVMK

NIENIELGLGEAQVIVVLASHLQNIEAEKQKLRTQVRRLCQENAWLRDELANTQQKLQSS

EQTVAQLEEEKKHLEFMASVKKYDDNQENEEQQEKSRS------DPVVEL-FPEDESEER

HTMSP-TPPN--QFANHANAGYEIPARLRTLHNLVIQYASQGRYEVAVPLCKQALEDLER

TSGHDHPDVATMLNILALVYRDQVCV----------------------------------

------------------------------------------------------------

---------------------------------------------

>Pabe_a_Q5R581

EKLTQDEIISKTKQVIQGLEALKNEHNSILQSLLETLKCLKKDD-ESN-LVEEKSNMIRK

SLEMLELGLSEAQVMMALSNHLNAVESEKQKLRAQVRRLCQENQWLRDELANTQQKLQKS

EQSVAQLEEEKKHLEFMNQLKKYDDDISPSEDKDTDST-----KEPLDDL-FPNDEDDPG

QGI-Q--QQHSSAAAAAQQGDYEIPARLRTLHNLVIQYASQGRYEVAVPLCKQALEDLEK

TSGHDHPDVATMLNILALVYRDQNKYKDAANLLNDALAIREKTLGKDHPAVAATLNNLAV

LYGKRGKYKEAEPLCKRALEIREKVLGKDHPDVAKQLNNLALLCQNQGKYEEVEYYYQRA

LEIYQTKLGPD-DPNVAKTKNNLASCYLKQGKFKQAETLYKEILT

>Drer_AAI55319

EKLSQEEIISNTRLVVQGLEALKSEHTSILQSLTETLRCLKKEE-ESS-LVQEKTSLLRR

SVEMIELGLGEAQVMMALSNHLNAVESEKQKLRAQVRRLCQENQWLRDELANTQQKLQRS

EQSVAQLEEDKKQLEFMNMLKKYDEDASPAEEKDGEPP-----KDSLDDL-FPNDEEEHT

QGMTQ--QHNSSAVAAAAQGGYEIPARLRTLHNLVIQYASQGRYEVAVPLCKQALEDLEK

TSGHDHPDVATMLNILALVYRDQNKYKEAAHLLNDALSIREKTLGKDHPAVAATLNNLAV

LYGKRGKYKEAEPLCKRALEIREKVLGKDHPDVAKQLNNLALLCQNQGKYEEVEYYYCRA

LEIYECRLGPD-DPNVAKTKNNLASCFLKQGKYKEAEVLYKEILT

>Cpip_CPIJ015771

--MTQEEIVSNTKTVLQGLEALRVEHLTLVSNLSEG---TEGS----------KREIVRQ

NIENIELGLGEAQVIVVLASHLQNIEAEKQKLRTQVRRLCQENAWLRDELANTQQKLQAS

EQSVAQLEEEKKHLEFMASVKKYDDNQEHEENLEKSRS------DPVVEL-FPEDESEER

QNMSP-TPAN--QFANHANAGYEIPARLRTLHNLVIQYASQGRYEVAVPLCKQALEDLEK

TSGHDHPDVATMLNILALVYRDQNKYKEAANLLNDALAIREKTLGENHPAVAATLNNLAV

LYGKRGKYKDAEPLCKRALEIRENVLGKNHPDVAKQLNNLALLCQNQTKYEEVEIYYQRA

LEIYEMKLGPD-DPNVAKTKNNLASCYLKQGKYKEAEVLYKQVLT

>Nvit_XP_001600608

TAMTQEEIVAGARTVAQGLEALRVEHTGLLNGLQSQE--APAAR--------DKASIISK

NIDMIELGLGEAQVMLALASHLQMVEAEKQKLRTQVRRLCQENAWLRDELAGTQQKLQAS

EQAVAQLEEEKRHLEFMASMRQYDPDPQPDDENAKDRP----KDDPVVDL-FPDDENEDR

NTMSP-TPPS--QFAQQVTAGYEIPARLRTLHNLVIQYASQGRYEVAVPLCKQALEDLEK

TSGHDHPDVATMLNILALVYRDQNKYKEAANLLNDALAIREKTLGENHPAVAATLNNLAV

LYGKRGKYKEAEPLCKRALEIREKVLGRDHPDVAKQLNNLALLCQNQGKYEEVEQYYQRA

LEIYEEKLGPD-DPNVAKTKNNLASCYLKQGKYKDAEVLYKQVLT

>Mmus_a_O88447

ENVTQDEIISKTKQVIQGLEALKNEHNSILQSLLETLKCLKKDD-ESN-LVEEKSSMIRK

SLEMLELGLSEAQVMMALSNHLNAVESEKQNVRAQVRRLCQENQWLRDELANTQQKLQKS

EQSVAQLEEEKKHLEFMNQLKKYDDDISPSEDKDSDSS-----KEPLDDL-FPNDEDEPG

QGI-Q--HSDSSAAAARQ--GYEIPARLRTLHNLVIQYASQGRYEVAVPSCKQALEDLEK

TSGHDHPDVATMLNILALVYRDQNKYKDAANLLNDALAIREKTLGRDHPAVAATLNNLAV

LYGKRGKYKEAEPLCKRALEIREKVLGKDHPDVAKQLNNLALLCQNQGKYEEVEYYYQRA

LGIYQTKLGPDRTPNVAKTKNNLASCYLKQGKFKQAETLYKEILT

>Dmel_NP_524049

TQMSQDEIITNTKTVLQGLEALRVEHVSIMNGIAEV---QKDN---------EKSDMLRK

NIENIELGLSEAQVMMALTSHLQNIEAEKHKLKTQVRRLHQENAWLRDELANTQQKFQAS

EQLVAQLEEEKKHLEFMASVKKYDENQEQDDACDKSRT------DPVVEL-FPDEENEDR

HNMSP-TPPS--QFANQT-SGYEIPARLRTLHNLVIQYASQGRYEVAVPLCKQALEDLER

TSGHDHPDVATMLNILALVYRDQNKYKEAANLLNDALSIRGKTLGENHPAVAATLNNLAV

LYGKRGKYKDAEPLCKRALEIREKVLGKDHPDVAKQLNNLALLCQNQGKYDEVEKYYQRA

LDIYESKLGPD-DPNVAKTKNNLAGCYLKQGRYTEAEILYKQVLT

>Rnor_a_P37285

EKLTQDEIISKTKQVIQGLEALKNEHNSILQSLLETLKCLKKDD-ESN-LVEEKSSMIRK

SLEMLELGLSEAQVMMALSNHLNAVESEKQKLRAQVRRLCQENQWLRDELANTQQKLQKS

EQSVAQLEEEKKHLEFMNQLKKYDDDISPSEDKDSDSS-----KEPLDDL-FPNDEDDPG

QGI-Q--QQHSSAAAAAQQGGYEIPARLRTLHNLVIQYASQGRYEVAVPLCKQALEDLEK

TSGHDHPDVATMLNILALVYRDQNKYKDAANLLNDALAIREKTLGRDHPAVAATLNNLAV

LYGKRGKYKEAEPLCKRALEIREKVLGKDHPDVAKQLNNLALLCQNQGKYEEVEYYYQRA

LEIYQTKLGPD-DPNVAKTKNNLASCYLKQGKFKQAETLYKEILT

>Tcas_XP_966804

TAMSQEEIVSAVRTVAQGLEALRSEHAGILHGLHEAP--DPVAN--------ERAGLVQQ

SAEMIELGLGEAQVIMALANHLQLIEAEKQKLRTQVRRLCQENAWLRDELASTQQRLQAS

EQTVAQLEEEKRHLEFMSSVSKYDQDVNDDNTSEHSRSE---KPDPVVDL-FPDDDNEDR

NNMSP-TPPNQLQLSQQVNAGYEIPARLRTLHNLVIQYASQGRYEVAVPLCKQALEDLEK

TSGHDHPDVATMLNILALVYRDQNKYKEAANLLNDALAIREKTLGENHPAVAATLNNLAV

LYGKRGKYKEAEPLCKRALDIREKVLGRDHPDVAKQLNNLALLCQNQGKYEEVEKYYQRA

LEIYEKRLGPD-DPNVSKTMNNLASCYLKQGKYKEAEVLYKQILN

>Ggal_b_XP_001233279

DKLSQEEIISNTKLVMQGLEALKNEHNSILHSLLETIKCLKKDE-EAN-LVHEKSNLLRK

SVEMIELGLGEAQVMMALSNHLNAVESEKQKLRAQVRRLCQENQWLRDELANTQQKLQRS

EQTVAQLEEEKKHLEFMNQLKKYDEDVSPSEEKEGDST-----KDSLDDL-FPNEEEEHG

PGLPH--QH-SSAVAAAQQGGYEIPARLRTLHNLVIQYASQGRYEVAVPLCKQALEDLEK

TSGHDHPDVATMLNILALVYRDQNKYKEAAHLLNDALCFTEKLIS---IQVAATLNNLAV

LYGKRGKYKEAEPLCKRALEIREKVLGKDHPDVAKQLNNLALLCQNQGKYEEVEYYYCRA

LEIYESCLGPD-DPNVAKTKNNLASCYLKQGKYKDAEVLYKEILT

>Cele_b_NP_502066

VISLGDDITTVLKTVQQTLFALRDEHEAATRILEANLINSDSSE---PSLPSEKMGLIDE

SLGKVMDGGDEASLLIMMDKLMQSYDVQLSKNHESIRLLRQENTWLLDELTTTQRKLQES

ERTVAHLEEERDHYKFQDSMNYLNSDFQHTTSVDATPMM----VDTLQELGFGPEEEDQN

NNQAD-QGCRSSSFSNPISNDYQLPTRLQTLQNLVIQYMEQGRFEVAIPLCKQALEDVVK

VHGNVHLDVATMLNVLAIVYRNQENFKDAAIYLEKALSIRVQCCGENHHSVAATLNNLAI

AYGKRGKYKESEPLCKRALEIRKNLLGPNHPDVAKQLTNLGIVTQQLEKYEETENYFKQA

LSIYNRAFPEN-HQNVIKTKNQLASVFLKQGKYQEAEEMYKNILS

>Cele_a_P46822.2

SNMSQDDVTTGLRTVQQGLEALREEHSTISNTLETSVKGVKEDE---APLPKQKLSQIND

NLDKLVCGVDETSLMLMVFQLTQGMDAQHQKYQAQRRRLCQENAWLRDELSSTQIKLQQS

EQMVAQLEEENKHLKYMASIKQFDDGTQSDTKTSVDVGPQPVTNETLQELGFGPEDEEDM

NASQFNQPTPANQMAASANVGYEIPARLRTLHNLVIQYASQGRYEVAVPLCKQALEDLEK

TSGHDHPDVATMLNILALVYRDQNKYKEAANLLNEALSIREKCLGESHPAVAATLNNLAV

LFGKRGKFKDAEPLCKRALEIREKVLGDDHPDVAKQLNNLALLCQNQGKYEEVEKYYKRA

LEIYESKLGPD-DPNVAKTKNNLSSAYLKQGKYKEAEELYKQILT

>Cpip_CPIJ002971

ATLTRTQIISNTKISMHVLDALKSQYTTLASHQASKVN-------------PFKLEILSK

NLANIEHGLDEAHLITTLATMLQSLEIEKERLRLQVKRLHQESAWLREELSSAQKRLQTS

EEEVIQLQEDKKHLEFTLSLKKYEDNEDHPLEKSHV--------DLIEEL-FTEDCSDGR

SP-----------TPPSQYSGYETPPRLRTLYNLVIEYASQGRYEVAVPLCQQALDDLEK

TSGHNHPDVATMLNILALVYRDQEKYREAVKLLIDALAIREKVLGENHPAVAATLNNLAV

LYGKCGKYKDAEPLCRRALIIRRNVLGEDHPDVAKQLTNLALICENLQKYTEVEKFYRKA

LEIYEAKLGPE-DPNVNKTKHNLGNCYVKLGEYQKAEALYNQILT

>Pbor_AAB87735

-MTASLGVAAAFELSWHALDEIAQQVAAVFSLFSLVEIPWKLVQ---QCLPKFDAEELED

ILDQMLLGAN-----LLGANLLKRVDQGMYQLHQLLREFFVVKREQRADDSDLQQRFYQV

VIAEAKRVRDEPEKSLIRESTMMIAHLQEAMERLARPEQALDLATCLNWL-AELYYVAQG

RYEEAEPLYVRSLSIHEQQLGADHLDVANSFNNLALLYKEQGRYEEAEPLYVRSLSIREQ

QLGTDHLDVATSLNNLAVLYRSQGRYEEAEPLYVRSLSIREQQLGTDHLDVATSLSNLAV

LYQSQGCHHKAEPLLVRALPIWEQQLGTDHPDVATSLNNLAFLYHLQGRYEEAEPLLVRA

LSIREQQLGTD-HPDVATSLNNLAVLYHLQGRYEDAEPLLLYSVR

>Spur_Q05090

GNLSQEQIITGTREVIKGLEQLKNEHNDILNSLYQSLKMLKKDTPGDSNLVEEKTDIIEK

SLESLELGLGEAKVMMALGHHLNMVEAEKQKLRAQVRRLVQENTWLRDELAATQQKLQTS

EQNLADLEVKYKHLEYMNSIKKYD---EDRTPDEEASS-----SDPLDLG-FPEDDDGGQ

ADESYPQPQTGSGSVSAAAGGYEIPARLRTLHNLVIQYASQSRYEVAVPLCKQALEDLEK

TSGHDHPDVATMLNILALVYRDQNKYKEAGNLLHDALAIREKTLGPDHPAVAATLNNLAV

LYGKRGKYKEAEPLCKRALEIREKVLGKDHPDVAKQLNNLALLCQNQGKYEEVEWYYQRA

LEIYEKKLGPD-DPNVAKTKNNLAAAYLKQGKYKAAETLYKQVLT

>Lpea_P46825

TALSQEEIISNTKTVIQGLDTLKNEHNQILNSLLTSMKTIRKEN-GDTNLVEEKANILKK

SVDSIELGLGEAQVMMALANHLQHTEAEKQKLRAQVRRLCQENAWLRDELANTQQKLQMS

EQKVATIEEEKKHLEFMNEMKKYDTN-EAQVNEEKESE-----QSSLDLG-FPDDDDDGG

QPEVLSPTQP-SAMAQAASGGCEIPARLRTLHNLVIQYASQGRYEVAVPLCKQALEDLEK

TSGHDHPDVATMLNILALVYRDQGKYKEAANLLNDALGIREKTLGPDHPAVAATLNNLAV

LYGKRGKYKDAEPLCKRALVIREKVLGKDHPDVAKQLNNLALLCQNQGKYEEVERYYQRA

LEIYQKELGPD-DPNVAKTKNNLASAYLKQGKYKQAEILYKEVLT

>Hsap_c_AAH73841

ERLSPEELVRQTRQVVQGLEALRAEHHGLAGHLAEALAGQGPAA-GLE-MLEEKQQVVSH

SLEAIELGLGEAQVLLALSAHVGALEAEKQRLRSQARRLAQENVWLREELEETQRRLRAS

EESVAQLEEEKRHLEFLGQLRQYDPP-AESQ-SESPPR-----RDSLASL-FPSEEEERK

G-------PEAAGAAAAQQGGYEIPARLRTLHNLVIQYAGQGRYEVAVPLCRQALEDLER

SSGHCHPDVATMLNILALVYRDQNKYKEATDLLHDALQIREQTLGPEHPAVAATLNNLAV

LYGKRGRYREAEPLCQRALEIREKVLGADHPDVAKQLNNLALLCQNQGKFEDVERHYARA

LSIYEALGGPH-DPNVAKTKNNLASAYLKQNKYQQAEELYKEILH

>Pabe_c_Q5R8E2

ERLSPEELVRQTRQVVQGLEALRAEHHGLVGHLAEALAGQGPVT-GLE-MLEEKQQVVSH

SLEAIELGLGEAQVLLALSAHVGALEAEKQRLRSQARRLAQENVWLREELEETQRRLRAS

EEAVAQLEEEKRHLEFLGQLRQYDPP-AESQQSESPPR-----RDSLASL-FPSEEEERK

G-------PEAAGAAAAQQGGYEIPARLRTLHNLVIQYAGQGRYEVAVPLCRQALEDLER

SSGHCHPDVATMLNILALVYRDQNKYKEATDLLHDALQIREQTLGPEHPAVAATLNNLAV

LYGKRGRYREAEPLCQRALEIREKVLGADHPDVAKQLNNLALLCQNQGKFEDVERHYARA

LSIYEALGGPH-DPNVAKTKNNLASAYLKQNKYQQAEELYKEILH

>Btau_c_Q2TBQ9

ERPSPEELVRQTRQVVKGLEALRAEHRGLAGHLAEALAAQGPAA-GLE-LLEEKQQVVSH

SLEAIELGLGEAQVLLALSAHVGALEAEKQRLRAQARRLAQENAWLREELEETQRRLRAS

EEAVAQLEEEKSHLEFLGQLRQYDPP-AESQQPESPPR-----RDSLASL-FPSEEEERR

G-------PEAVGAAAAQQGGYEIPARLRTLHNLVIQYAGQGRYEVAVPLCRQALEDLER

SSGHCHPDVATMLNILALVYRDQNKYKEATDLLHDALQIREQTLGPEHPAVAATLNNLAV

LYGKRGRYREAEPLCQRALEIREKVLGADHPDVAKQLNNLALLCQNQGKFEEVERHYARA

LSIYEALGGPH-DPNVAKTKNNLASAYLKQNKYQQAEELYKEILH

>Rnor_b_Q68G30

ERLNPEELVRQTRQVVQGLEALRAEHHSLAGHLAEALAGPGPVA-GVE-LLEEKQQVVNH

SLEAIELGLGEAQVLLALSAHVGVLEAEKQRLRAQARRLAQENTWLREELEETQRRLRAS

EEAVAQLEEEKSHLQFLGQLRQYDPP-EESQRPDSPPR-----RDSLASL-FPSEEEEKK

G-------PEAAGAAAAQQGGYEIPARLRTLHNLVIQYASQGRYEVAVPLCRQALEDLER

SSGHCHPDVATMLNILALVYRDQNKYKEATELLHDALQIREQTLGPEHPAVAATLNNLAV

LYGKRGRYREAEPLCQRALEIREKVLGADHPDVAKQLNNLALLCQNQGKFQDVERHYARA

LSIYEALGGPQ-DPNVAKTKNNLASAYLKQNKYQQAEELYKEILS

>CKstut_CAJ73558

------------------------------------------------------------

------------------------------------------------------------

-----------------------MSKLINSIKSFRRHG----------------------

----TKIILTLSLVVSFILPAYAQENLWKELNDKTTTLLQEKRYADAIKSGEDALRIAKE

TFPPGHISIAASMNLLGILYRTYTMYDEAEPLFNQALDIYRETNGTDHPTVAYVLQELAE

MFLLQDNYAKAEPLYKQSLGIYENVSGQDNPGIVNILNRLGEIYQHQEKYADAILFYKRA

LAIEVEIFGND-HPDVASSMNNLATLYYHNGENTKAESLYKQALE

>Mmus_c_NP_666294

ERLNPEELVRQTRQVVQGLEALRAEHHSLAGHLAEALAGPGPVA-GVE-LLEEKQQVVNH

SLEAIELGLGEAQVLLALSAHVSVLEAEKQRLRAQARRLAQENTWLREELEETQRRLRAS

EEAVAQLEEEKSHLQFLGQLRQYDPP-EESQRPESPPR-----RDSLASL-FPSEEEEKK

G-------PEAAGAAAAQQGGYEIPARLRTLHNLVIQYAGQGRYEVAVPLCRQALEDLER

SSGHCHPDVATMLNILALVYRDQNKYKEATELLHDALQIREQTLGPEHPAVAATLNNLAV

LYGKRGRYREAEPLCQRALEIREKVLGADHPDVAKQLNNLALLCQNQGKFQDVERHYARA

LSIYEALGGPQ-DPNVAKTKNNLASAYLKQNKYQQAEELYKEILS

>Btau_d_AAI05321

HRLSQEEILGSTRLVSQGLEALHSEHQAVLQSLSQTIECLQQGG-HEEGLVHEKARQLRR

SMENIELGLSEAQVMLALANHLSTVESEKQKLRAQVRRLCQENQWLRDELAGTQQRLQRS

EQAVAQLEEEKKHLEFLGQLRQYDEDGHAAEEKEGDAS-----KDSLDDL-FPNEEEEDP

SNG----LSRGQG---AQHSGYEIPARLRTLHNLVIQYAAQGRYEVAVPLCKQALEDLER

TSGRGHPVVATMLNILALVYRGQNKYKEAALLLNDALSIRESTLGRDHPAVAATLNNLAV

LYGKRGKYKEAEPLCQRALEIREKVLGTNHPDVAKQLNNLALLCQNQGKYEAVERYYRRA

LAIYEGQLGPD-NPNVARTKNNLASCYLKQGKYAEAETLYKEILT

>Rnor_c_NP_001009601

HRLSQEEILGSTRLVSQGLESLHSEHQAVLQSLSHTIECLQQGG-HEEGLVHEKARQLRR

SMENIELGLSEAQVMLALASHLSTVESEKQKLRAQVRRLCQENQWLRDELAGTQQRLQRS

EQAVAQLEEEKKHLEFLRQLRQYDEDGHSMEEKEGDAS-----KDSLDDL-FPNEEEEDS

SND----LSRGQGAAAAQQGGYEIPARLRTLHNLVIQYAAQGRYEVAVPLCKQALEDLER

TSGRGHPDVATMLNILALVYRDQNKYKEAAHLLNDALSIRESTLGRDHPAVAATLNNLAV

LYGKRGKYKEAEPLCQRALEIREKVLGTDHPDVAKQLNNLALLCQNQGKYEAVERYYQRA

LAIYERQLGPD-NPNVARTKNNLASCYLKQGKYSEAETLYKEILT

>Ptro_XP_524303

ERLSPEELVRQTRQVVQGLEALRAEHHGLSGHLAEALAGQGPAT-GLE-MLEEKQQVVSH

SLEAIELGLGEAQVLLALSAHVGALEAEKQRLRSQARRLAQENVWLREELEETQRRLRAS

EESVAQLEEEKRHLEFLGQLRQYDPP-AESQQSESPPR-----RDSLASL-FPSEEEERK

G-------PEAAGAAAAQQGGYEIPARLRTLHNLVIQYAGQGRYEVAVPLCRQALEDLER

SSGHCHPDVATMLNILALVYRDQNKYKEAIDLLHDALQIREQTLGPEHPAVAATLNNLAV

LYGKRGRYREAEPLCQRALEIREKVLGADHPDVAKQLNNLALLCQNQGKFEDVERHYARA

LSIYEALGGPH-DPNVAKTKNNLASAYLKQNKYQQAEELYKEILH

>Mmus_d_Q9DBS5

HRLSQEEILGSTKVVSQGLEALHSEHQAVLQSLSHTIECLQQGG-HEEGLVHEKARQLRR

SMENIELGLSEAQVMLALASHLSTVESEKQKLRAQVRRLCQENQWLRDELAGTQQRLQRS

EQAVAQLEEEKKHLEFLRQLRQYDEDGHGMEEKEGEAT-----KDSLDDL-FPNEEEEDS

GND----LSRGQGAAAAQQGGYEIPARLRTLHNLVIQYAAQGRYEVAVPLCKQALEDLER

TSGRGHPDVATMLNILALVYRDQNKYKEAAHLLNDALSIRESTLGRDHPAVAATLNNLAV

LYGKRGKYKEAEPLCQRALEIREKVLGTDHPDVAKQLNNLALLCQNQGKYEAVERYYQRA

LAIYESQLGPD-NPNVARTKNNLASCYLKQGKYSEAEALYKEILT

>Mmus_b_O88448

EKLSQDEIVLGTKAVIQGLETLRGEHRALLAPLASHEAGEAEPG-SQE-----RCLLLRR

SLEAIELGLGEAQVILALSSHLGAVESEKQKLRAQVRRLVQENQWLREELAGTQQKLQRS

EQAVAQLEEEKQHLLFMSQIRKLDEMLP-QEEKGDVP------KDSLDDL-FPNEDEQSP

AP------SPGGGDVAAQHGGYEIPARLRTLHNLVIQYASQGRYEVAVPLCKQALEDLEK

TSGHDHPDVATMLNILALVYRDQNKYKDAAHLLNDALAIREKTLGKDHPAVAATLNNLAV

LYGKRGKYKEAEPLCKRALEIREKVLGKFHPDVAKQLSNLALLCQNQGKAEEVEYYYRRA

LEIYATRLGPD-DPNVAKTKNNLASCYLKQGKYQDAETLYKEILT

>Pabe_b_NP_001125516

EKLGQDEIVLGTKAVIQGLETLRGEHRALLAPLVAPEAGEAEPG-SQE-----RCILLRR

SLEAIELGLGEAQVILALSSHLGAVESEKQKLRAQVRRLVQENQWLREELAGTQQKLQRS

EQAVAQLEEEKQHLLFMSQIRKLDEDASPNEEKGDVP------KDTLDDL-FPNEDEQSP

AP------SPGGGDVSGQHGGYEIPARLRTLHNLVIQYASQGRYEVAVPLCKQALEDLEK

TSGHDHPDVATMLNILALVYRDQNKYKEAAHLLNDALAIREKTLGKDHPAVAATLNNLAV

LYGKRGKYKEAEPLCKRALEIREKVLGKFHPDVAKQLSNLALLCQNQGKAEEVEYYYRRA

LEIYATRLGPD-DPNVA----------------------------
